# In Vivo Cleavage Map Illuminates the Central Role of RNase E in Coding and Non-coding RNA Pathways

**DOI:** 10.1016/j.molcel.2016.11.002

**Published:** 2017-01-05

**Authors:** Yanjie Chao, Lei Li, Dylan Girodat, Konrad U. Förstner, Nelly Said, Colin Corcoran, Michał Śmiga, Kai Papenfort, Richard Reinhardt, Hans-Joachim Wieden, Ben F. Luisi, Jörg Vogel

**Affiliations:** 1 Institute of Molecular Infection Biology, University of Würzburg, 97080 Würzburg, Germany; 2 Core Unit Systems Medicine, University of Würzburg, 97080 Würzburg, Germany; 3 Alberta RNA Research and Training Institute, Department of Chemistry and Biochemistry, University of Lethbridge, Lethbridge, Alberta T1K 3M4, Canada; 4 Department of Biology I, Microbiology, Ludwig-Maximilians-Universität Munich, 82152 Martinsried, Germany; 5 Laboratory of Structural Biochemistry, Freie Universität Berlin, 14195 Berlin, Germany; 6 Max Planck Genome Centre Cologne, Max Planck Institute for Plant Breeding Research, 50829 Cologne, Germany; 7 Department of Biochemistry, University of Cambridge, Cambridge CB2 1GA, UK; 8 Helmholtz Institute for RNA-based Infection Research (HIRI), 97080 Würzburg, Germany

**Keywords:** RNase E, RNA degradome, non-coding RNA, Hfq, 3′ UTR, ArcZ, RprA, sRNA maturation, uridine ruler, TIER-seq

## Abstract

Understanding RNA processing and turnover requires knowledge of cleavages by major endoribonucleases within a living cell. We have employed TIER-seq (transiently inactivating an endoribonuclease followed by RNA-seq) to profile cleavage products of the essential endoribonuclease RNase E in *Salmonella enterica*. A dominating cleavage signature is the location of a uridine two nucleotides downstream in a single-stranded segment, which we rationalize structurally as a key recognition determinant that may favor RNase E catalysis. Our results suggest a prominent biogenesis pathway for bacterial regulatory small RNAs whereby RNase E acts together with the RNA chaperone Hfq to liberate stable 3′ fragments from various precursor RNAs. Recapitulating this process in vitro, Hfq guides RNase E cleavage of a representative small-RNA precursor for interaction with a mRNA target. In vivo, the processing is required for target regulation. Our findings reveal a general maturation mechanism for a major class of post-transcriptional regulators.

## Highlights

• TIER-seq precisely maps ∼22,000 endogenous RNase E cleavage sites in *Salmonella*
• Consensus motif of RNase E reveals a 2-nt uridine ruler-and-cut mechanism
• RNase E is a central component in both maturation and degradation of small RNAs
• There is a general small-RNA biogenesis pathway requiring RNase E and Hfq

## Graphical Abstract

**Figure:**
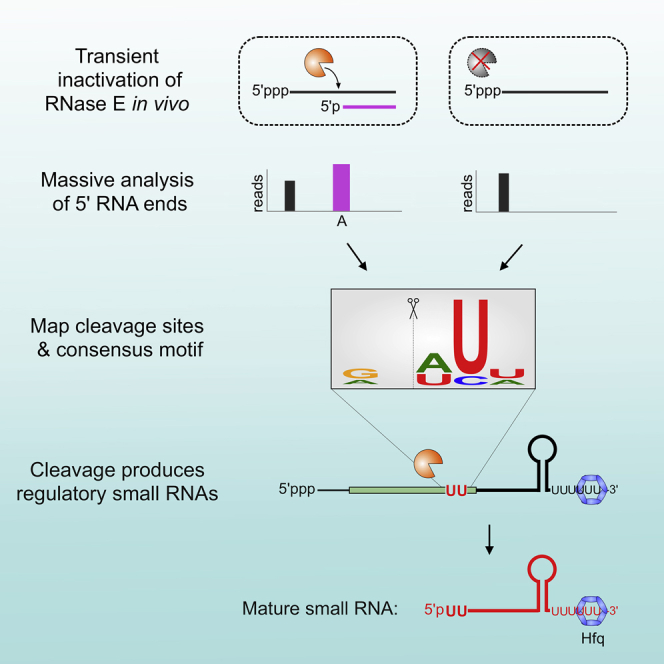

## Teaser

Chao et al. discover that the essential bacterial RNase E cleaves numerous transcripts at preferred sites by sensing uridine as a 2-nt ruler. RNase E processing of various precursor RNAs produces many small regulatory RNAs, constituting a major small-RNA biogenesis pathway in bacteria.

## Introduction

Small, non-coding RNAs (sRNAs) that associate with the RNA chaperone Hfq constitute the largest class of post-transcriptional regulators in Gram-negative bacteria (De Lay et al., 2013, Storz et al., 2011, Vogel and Luisi, 2011, Wagner and Romby, 2015). Initially defined as a class in non-pathogenic *Escherichia coli* (Zhang et al., 2003), Hfq-dependent sRNAs have been globally mapped in numerous important human pathogens (Barquist and Vogel, 2015, Holmqvist et al., 2016, Koo et al., 2011, Melamed et al., 2016, Tree et al., 2014). These sRNAs generally act as multi-target repressors and activators through seed pairing interactions with the 5′ untranslated region (UTR) of mRNAs (Desnoyers et al., 2013, Feng et al., 2015, Papenfort and Vanderpool, 2015). A full understanding of these sRNA-mediated networks requires knowledge of how their RNA constituents are synthesized and turned over.

Many of the bacterial sRNAs characterized to date are transcribed from non-coding intergenic regions and operate as full-length, primary transcripts capped with a 5′ triphosphate (5′ PPP). However, some primary sRNAs such as ArcZ and RprA are converted into shorter stable species that retain the seed region for target mRNA recognition (Mandin and Gottesman, 2010, Papenfort et al., 2009, Papenfort et al., 2015). It is currently unclear whether such processing generates the active sRNAs, as is the case with eukaryotic microRNAs (Kim, 2005). Moreover, several recent studies reported sRNAs that are produced from the 3′ region of mRNA genes (Miyakoshi et al., 2015b), only a subset of which are the result of gene-internal promoters (Chao et al., 2012, Guo et al., 2014), while many others appear to originate from mRNA processing. These 3′-derived sRNAs are likely to be functional, since they abundantly associate with Hfq (Chao et al., 2012), whose cellular concentration is limited (Wagner, 2013). Their physiological importance is further supported by established roles of the 3′-mRNA-derived sRNAs CpxQ and SroC in the envelope stress response or amino acid pathways, respectively (Chao and Vogel, 2016, Miyakoshi et al., 2015a). Furthermore, 3′ fragments of *E. coli* tRNA precursors function as molecular sponges of conserved sRNAs (Lalaouna et al., 2015). Collectively, these findings suggest that sRNA processing is a prevalent event; however, both its functional relevance and the major responsible nuclease(s) remain to be established.

Of several candidate nucleases involved in sRNA processing and turnover, the conserved and essential endoribonuclease E (RNase E) is the likely central player (Mackie, 2013, Massé et al., 2003, Saramago et al., 2014). It can be inferred, from transcript accumulation upon its inactivation, that RNase E drives the decay of most mRNAs in *E. coli* (Bernstein et al., 2004, Clarke et al., 2014), and in *Salmonella* it processes the mRNA 3′ end-derived CpxQ and SroC sRNAs (Chao and Vogel, 2016, Miyakoshi et al., 2015a). RNase E also degrades several sRNAs in the absence of Hfq or upon base pairing with target mRNAs (Bandyra et al., 2012, Massé et al., 2003, Moll et al., 2003). Conversely, some sRNAs activate gene expression by blocking RNase E cleavage sites in target mRNAs (Fröhlich et al., 2013, Papenfort et al., 2013). In addition, RNase E is known to engage in rRNA and tRNA precursor processing (Apirion and Lassar, 1978, Bessarab et al., 1998, Kime et al., 2014, Li and Deutscher, 2002, Ow and Kushner, 2002).

Despite the importance of RNase E in post-transcriptional control, its activity toward most non-coding RNAs is not known. Previous studies have characterized major RNase E cleavage sites in a few abundant model transcripts (e.g., Apirion and Lassar, 1978, Delvillani et al., 2011, Ehretsmann et al., 1992, Mackie, 1991, Ow and Kushner, 2002, Patel and Dunn, 1992, Régnier and Hajnsdorf, 1991, Roy and Apirion, 1983) and concluded that the enzyme preferentially cleaves AU-rich regions in single-stranded RNA (Arraiano et al., 2010, Huang et al., 1998, McDowall et al., 1994, McDowall et al., 1995). Here, to achieve a systems-level understanding of RNase E activity, we have analyzed in depth the in vivo RNase E cleavage events in *Salmonella typhimurium*, a close relative of *E. coli* and a pathogenic model organism to study post-transcriptional regulation (Westermann et al., 2016). Our genome-wide capture of tens of thousands of endogenous cleavage sites reveals a minimal consensus sequence and a 2-nt uridine ruler-and-cut structural mechanism for this major endoribonuclease. Intriguingly, RNase E employs this mechanism to cleave many coding and non-coding transcripts at the 3′ end and releases stable, Hfq-bound RNA fragments, indicating that sRNA biogenesis through endonucleolytic processing is widespread. Searches for these predicted critical uridines in sRNAs enabled us to show that maturation by RNase E is essential for target regulation by the ArcZ sRNA. Moreover, our data reveal a high frequency of RNase-E-mediated cleavages in Hfq-dependent sRNAs, supporting the functional link between RNase E and Hfq for the first time on a global level.

## Results

### A Transcriptome-wide Map of RNase E Cleavage Sites In Vivo

**Figure 1:**
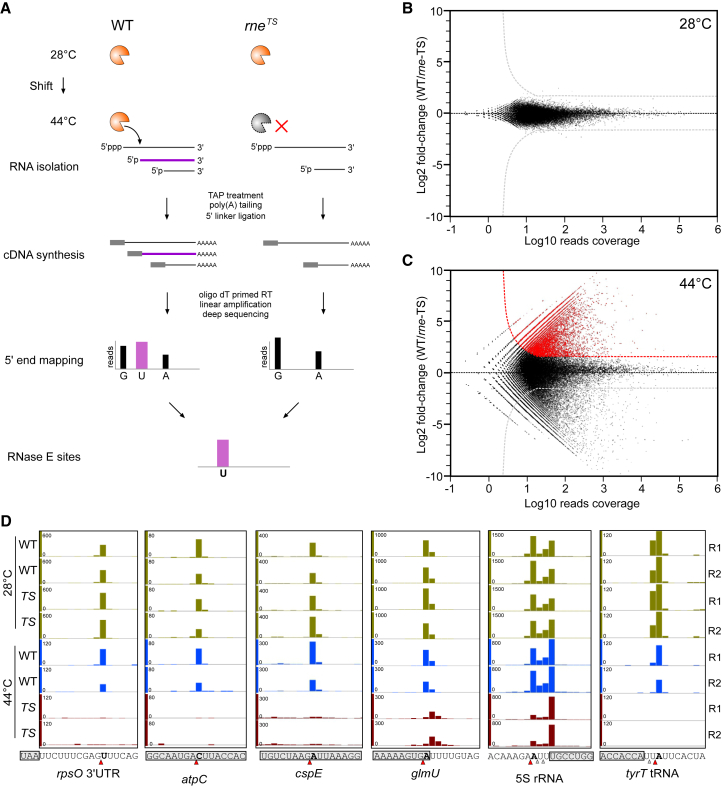
Global Mapping of Endogenous RNase E Cleavage Sites in *Salmonella* using TIER-Seq (A) Schema of the TIER-seq approach. Endogenous cleavage sites were identified by analyzing the 5′ ends of RNase E cleavage products (purple) in the WT and *rne*^TS^ strains at the non-permissive temperature (44°C). Total RNA from WT and *rne*^TS^ was converted to cDNAs and sequenced; the 5′ ends depleted in the *rne*^TS^ libraries at 44°C indicate the RNase E cleavage sites (e.g., purple U). (B and C) Global analysis of 5′ end profile at the permissive temperature 28°C (B) and non-permissive temperature 44°C (C). The plots show the read counts for every 5′ base in WT samples and the relative fold change compared to *rne*^TS^ samples. Candidate RNase E cleavage sites that show >3-fold depletion in *rne*^TS^ samples (p < 0.05, FDR < 0.05) are colored in red. (D) TIER-seq captures known RNase E cleavage sites with single-nucleotide resolution. *TS* indicates the *rne*^TS^ samples. R1 and R2 are two biological replicates. The major RNase E sites are marked by red arrowheads and bold lettering; secondary cleavage sites are indicated by open arrowheads. The ORF or mature RNAs are shadowed by gray boxes. See also Figures S1 and S2.

To globally map RNase E cleavage events in vivo, we profiled 5′ ends of cellular transcripts by comparative RNA-seq before and 30 min after programmed inactivation of the enzyme using a temperature-sensitive *rne*^TS^ mutant (*rne-*3071) (Apirion and Lassar, 1978, Figueroa-Bossi et al., 2009). We refer to this approach, which builds upon work by Clarke and colleagues (Clarke et al., 2014) as transient inactivation of endoribonuclease followed by RNA-seq (TIER-Seq; see Figure 1A). At the permissive temperature (28°C), *Salmonella* wild-type (WT) *rne* and mutant *rne*^TS^ strains both exhibit full RNase E activity, whereas upon shift to 44°C, only WT RNase E retains its activity to process RNA. To achieve a comprehensive RNase-E-specific “degradome” analysis at single-nucleotide resolution (Figure 1A), we analyzed biological duplicates of all four of the above strains and conditions in the early stationary growth phase (OD_600_ of 2) by RNA-seq, obtaining ∼130 million reads (Figure S1A). In agreement with previous work showing that RNase E cleaves AU-rich sequences (McDowall et al., 1994, McDowall et al., 1995), the inactivation of RNase E leads to a ∼5% reduction of cDNA reads with 5′-A/T bases (Figure S1B).

To pinpoint cleavage sites, we aligned all reads to the *Salmonella* genome, mapping a total of ∼500,000 unique 5′ ends (Figures 1B and 1C). WT and *rne*^TS^ samples from growth at 28°C gave nearly identical 5′ end profiles (*R*^2^ = 0.98; Figures 1B and S1C), confirming that the mutant RNase E is fully functional at the permissive temperature, whereas at the non-permissive temperature (44°C), many positions were selectively depleted in the *rne*^TS^ cDNA libraries (Figure 1C). Since *Salmonella* has no 5′→3′ exoribonuclease (Hui et al., 2014), we interpret these depleted positions as RNase E cleavage sites (Figure 1A). This classification is supported by the capture of many previously known *E. coli* RNase E cleavage sites (Figure 1D)—for example, in the *rpsO*, *cspE*, *uncC/atpC*, and *glmUS* mRNAs (Delvillani et al., 2011, Joanny et al., 2007, Patel and Dunn, 1992, Régnier and Hajnsdorf, 1991), in the 9S precursor of 5S rRNA (Roy and Apirion, 1983), and near the 3′ end of tRNAs (Ow and Kushner, 2002). Applying a threshold of >3-fold as significant depletion (p < 0.05, FDR < 0.05) in the *rne*^TS^ samples at 44°C, we assigned 22,033 RNase-E-mediated cleavages in the *Salmonella* transcriptome, expanding by several orders of magnitude the database of in vivo target sites for this ribonuclease. The full list of cleavage sites is available in Table S1.

### A Systems-Level View on RNase E Activity in RNA Metabolism

**Figure 2:**
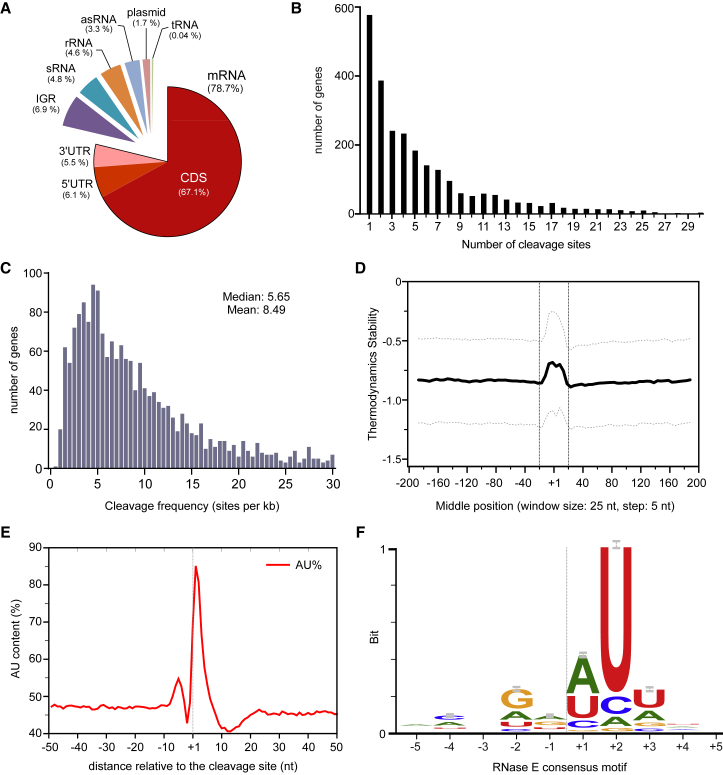
Systems-wide Analysis of Cleavage Sites Reveals a Consensus for RNase E (A) Classification of all RNase E cleavage sites. The proportion (%) of all sites mapped within a category is shown. See also Table S1. (B) The number of cleavage sites mapped per mRNA gene. (C) The distribution of RNase E cleavage frequencies in mRNAs (RPKM > 10). See also Figure S1. (D) Sequences at the RNase E sites are less structured. Minimal folding energy (MFE) was calculated for each 25 nt using a sliding window and was compared to randomly shuffled sequences. Median *Z* score is shown as a bold line; dotted lines indicate the upper and lower quartile. (E) Distribution of AU content at the RNase E cleavage sites. Dashed line indicates the cleavage site (+1 nt). (F) The RNase E consensus motif based on alignment of all mapped cleavage sites. Error bars indicate 95% confidence intervals. See also Figures S1 and S2.

Systematic analysis of the 22,033 RNase E cleavage sites revealed their distribution in coding and non-coding transcripts from the *Salmonella* chromosome and virulence plasmids (Figure 2A): ∼80% occurred in mRNAs, primarily in the coding sequence (CDS), indicating that a major activity of RNase E is to degrade mRNAs in addition to processing housekeeping RNAs. Altogether, we detected a total of 2,557 mRNAs cleaved by RNase E, with a different number of cleavage sites per transcript (Figure 2B); these represent 78% of 3,286 *Salmonella* mRNAs that are well expressed (RPKM > 10, Table S2) in the early stationary phase. Notably, the assay captured many essential genes and virulence genes required for intracellular growth (Table S3), which provide insights into the processing of transcripts from indispensable genes and the roles of RNase E in *Salmonella* pathogenesis (Viegas et al., 2013), respectively. Longer transcripts generally tend to contain a higher number of cleavage sites (Figures S1D and S1E). After normalizing the number of cleavage sites to gene length, RNase E cleavage frequency in these genes (RPKM > 10) ranges from 0 to ≥30 sites per kilobase, with a median value at ∼5.7 cleavages per kilobase, or one site every ∼175 nt of mRNA (Figure 2C). This non-saturating cleavage pattern might suggest that most sites in mRNAs are inaccessible, perhaps due to structural constraints or protein binding.

The position of an RNase E site within a transcript may provide information about the function of the cleavage. For example, RNase E auto-regulates its synthesis by cutting in the 5′ UTR of its own mRNA (Jain and Belasco, 1995); our analysis readily captured this critical site (Figure S2A). As another example, we detect the RNase E site in the 5′ UTR of *cfa* mRNA (Figure S2A) that becomes protected by the *trans*-acting RydC sRNA, with the consequence that the transcript is stabilized (Dimastrogiovanni et al., 2014, Fröhlich et al., 2013). Thus, our candidate list of ∼1,300 RNase E cleavage sites identified in the 5′ UTRs of 548 genes (Table S4) provides a resource to predict sites for post-transcriptional control by sRNAs and/or RNA-binding proteins.

### A Specific Sequence Motif Recognized by RNase E

Even seemingly non-specific nucleases often exhibit a certain degree of sequence or structural preference. To understand the substrate determinants of RNase E activity, we analyzed the primary sequences and putative secondary structures around all of the 22,033 cleavage sites. At the cleavage site we observed an overall increase in the calculated folding energy (Δ*G*), indicating little secondary structure (Figure 2D), and a spike of AU-rich sequences (Figure 2E), both of which agree with previously studied individual RNase E sites (McDowall et al., 1994, McDowall et al., 1995). Importantly, sequence alignment of all 22,033 sites predicts a minimal RNase E consensus sequence (Figure 2F) with a marked preference for uridine at the +2 position in the 5 nt “RN↓WUU” core motif (with R as G/A, W as A/U, and N as any nucleotide). This RNase E motif, based entirely on global in vivo data, fully recapitulates preferences previously documented with model substrates in vitro (Ehretsmann et al., 1992, Kaberdin, 2003, Mackie, 1991) and with cell-derived RNA (Del Campo et al., 2015), while it clearly differs from recognition motifs of other major bacterial endoribonucleases such as tRNA-processing RNase P (McClain et al., 1987) or RNase III, which cleaves double-stranded RNA (Gan et al., 2005).

### RNase E Cleavages Underlie sRNA Biogenesis from 3′ UTRs

**Figure 3:**
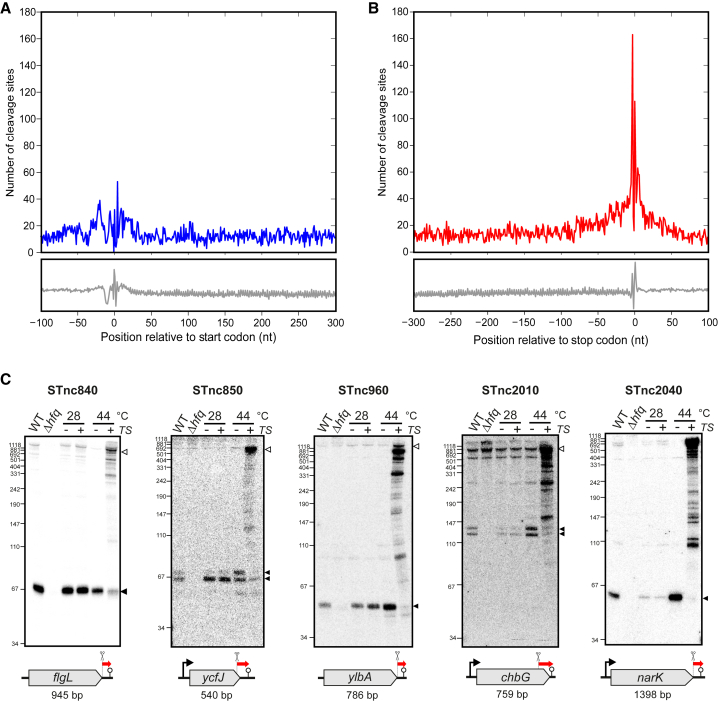
RNase E Cleaves mRNAs to Produce 3′ UTR-Derived sRNAs (A and B) Distribution of RNase E cleavage sites in mRNAs relative to their start codon (A) or stop codon (B). The gray lines in the lower panel indicate the distribution of consensus motif based on genomic sequence. (C) RNase E and Hfq are required for the biogenesis of 3′ UTR-derived sRNAs. WT and Δ*hfq* strains were grown at 37°C to an OD_600_ of 2. The location of sRNAs (red arrows) and host genes are shown in the lower panel. Promoters (where available) and terminators are shown. The 5S rRNA served as loading control (Figure S2C). See also Figures S2, S3, and S4.

In analyzing cleavage-site distributions relative to mRNA start or stop codons (Figures 3A and 3B), we observed that, on average, 5′ UTRs and the coding regions showed similar cleavage frequencies. Translation initiation regions were slightly counter-selected, perhaps because the prominent Shine-Dalgarno sequence (GGAGGA) is devoid of RNase E cleavage motifs. In contrast, RNase E sites were enriched around mRNA stop codons (Figure 3B); the high AU-rich content and/or translation termination may favor this enrichment. Since bacterial 3′ UTRs are generally short (Belasco, 2010), many of these stop codon sites may represent the most downstream sites, leaving 3′ fragments for degradation by 3′→5′ exoribonucleases. Interestingly, approximately one-third of these mRNAs carry protective ρ-independent terminators (Arraiano et al., 2010) that can, in principle, interact with the sRNA chaperone Hfq (Otaka et al., 2011, Sauer and Weichenrieder, 2011). These data point to the possibility that stable 3′ UTR fragments accumulate with functional consequence in the guise of regulatory sRNAs (Table S5; Chao et al., 2012, Miyakoshi et al., 2015b). Indeed, we have detected the mRNA 3′ UTR processing sites that produce the CpxQ and SroC sRNAs (Figure S2B). Northern blot probing of several selected candidates revealed distinct RNA species from mRNA 3′ ends, the generation of which required both active RNase E and the presence of Hfq (Figures 3C and S2C). Most of these 3′-derived sRNAs co-accumulate with their parental mRNA transcripts and possess potential seed regions (Figure S3), suggesting that they are bona fide regulatory sRNAs with conserved targets and functions. In addition, the cleavage sites in these sRNAs resemble the “RNWUU” sequence (Figure S2D), supporting the recognition of this consensus by RNase E (Figure 2F).

### Cleavage by RNase E Produces sRNAs from Non-coding RNA Precursors

The majority of well-characterized, Hfq-dependent sRNAs in *E. coli* and *Salmonella* are primary transcripts of 50–250 nt in length. Although previous work on a few model sRNAs has implicated RNase E in their decay (Göpel et al., 2013, Madhugiri et al., 2010, Miyakoshi et al., 2015a, Viegas et al., 2007), it is unknown whether this sRNA class is generally processed by RNase E. Here, we have mapped ∼600 RNase E cleavage sites in 107 experimentally validated sRNAs (Table S6), corroborating previously proposed sites in model sRNAs such as DsrA and MicA (Figures S4A and S4B; Moll et al., 2003). RNase E seems to preferentially target sRNAs that are bound by Hfq, as there are more cleavage sites in Hfq-dependent sRNAs compared to those that are Hfq independent (Chao et al., 2012; Figure S4C). Additionally, many cleavage sites in these sRNAs mapped to the vicinity of the seed region (Figure S4D), as exemplified by their clustering in the well-characterized seed of SgrS and RybB (Figures S4E and S4F). These data suggest that RNase E may inactivate sRNAs by removing the seed region; this is in agreement with previous results for MicC (Bandyra et al., 2012) and RyhB (Massé et al., 2003, Moll et al., 2003). Both MicC and RyhB are turned over by RNase E through seed cleavage if the target is absent, and this could provide a surveillance mechanism for accurate seed matching (Bandyra et al., 2012).

**Figure 4:**
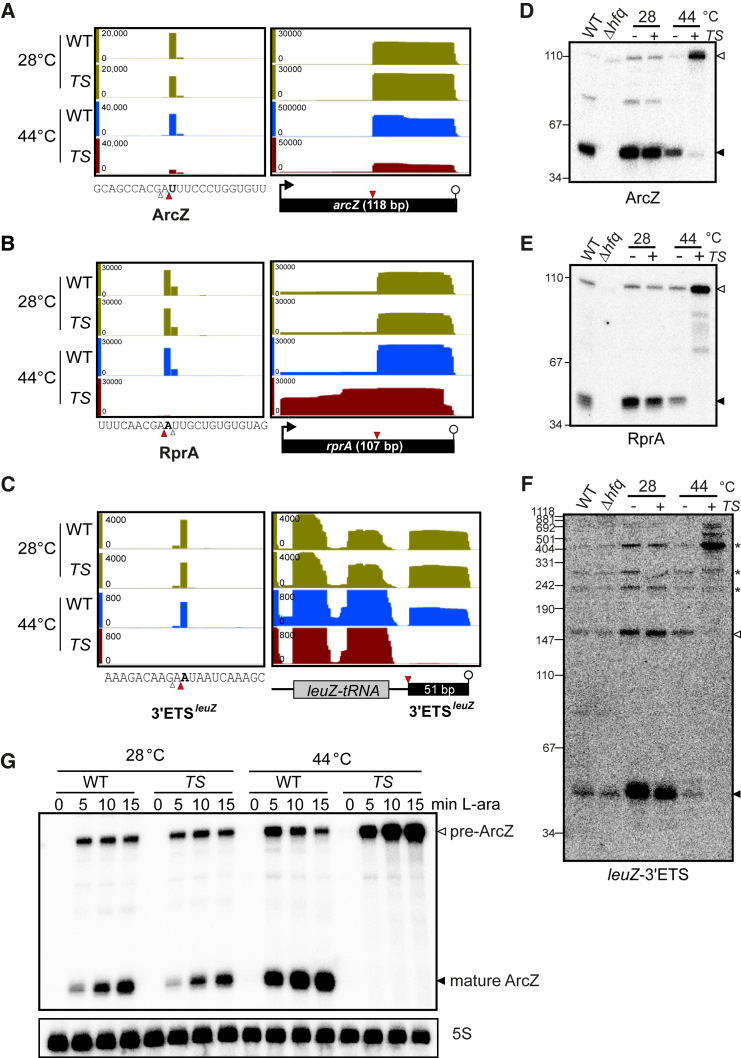
RNase E Cleaves Non-coding RNAs to Release 3′ Mature sRNAs (A–C) RNase E cleavage sites are identified in the ArcZ sRNA (A), RprA (B), and 3′ETS^leuZ^ (C). The major sites are marked by red arrowheads and bold lettering, whereas the minor sites are indicated by open arrowheads. See also Figures S5 and S6. (D–F) RNase E is required for the processing of ArcZ (D), RprA (E), and 3′ETS^leuZ^ (F). Open arrowheads indicate precursor fragments and filled arrowheads indicate processed mature species. ^∗^ indicates longer precursors of polycistronic LeuZ-tRNA fragments; 5S loading controls, see Figure S2C. (G) The maturation of ArcZ is dependent on RNase E activity. Expression of the full-length ArcZ precursor (pre-ArcZ) was induced by L-arabinose.

Another group of sRNAs is spared from immediate degradation following RNase E cleavage; instead, these RNAs appear to be processed by the enzyme. The highly conserved ArcZ and RprA sRNAs, which each regulate a number of targets, including *rpoS* (Majdalani et al., 2001, Mandin and Gottesman, 2010, Papenfort et al., 2009, Papenfort et al., 2015), provide cogent examples in which RNase E converts a precursor into a stable, shorter sRNA form (Figure 4). For both ArcZ and RprA, the detected cleavage sites precisely match the RNase E consensus motif (Figures 4A–4C) and are fully consistent with the size of the previously documented ∼50 nt 3′ species of these sRNAs (Argaman et al., 2001, Mandin and Gottesman, 2010, Papenfort et al., 2009, Papenfort et al., 2015). These 3′ species accumulated to significantly higher levels than the primary sRNAs in an Hfq-dependent manner (Figures 4D and 4E). When RNase E was inactivated for 30 min, these shorter ArcZ and RprA species became undetectable on northern blots (Figures 4D and 4E; lane *rne*^TS^, 44°C), suggesting a primary role for the enzyme in the processing event. To independently evaluate the function of RNase E in processing these sRNAs in vivo, each RNA was expressed from a plasmid-borne promoter subsequent to heat inactivation of the enzyme. While the full-length sRNAs accumulated under this condition, they were not converted into the short 3′ species (Figures 4G and S5B). These findings establish RNase E as a primary nuclease for generating functional short ArcZ and RprA, both of which regulate numerous *trans*-encoded target mRNAs (Papenfort et al., 2009, Papenfort et al., 2015).

Hfq-dependent regulatory RNA can also originate from other types of precursors, such as polycistronic tRNA transcripts. One such precursor is the sRNA sponge *leuZ*-3′ETS (Lalaouna et al., 2015), which was suggested to be processed by RNase E during *leuZ*-tRNA maturation (Li and Deutscher, 2002). Our TIER-seq data confirm that the 5′ end of *leuZ*-3′ETS is generated by RNase E and pinpoints the cleavage site to an adenine 15 nt downstream of the mature *leuZ*-tRNA (Figure 4C). Using the *rne*^TS^ strain, we observe RNase E to be essential for the production of this sRNA sponge (Figure 4F). Together, these results argue for a major role of RNase E in maturing non-coding regulatory RNAs from different types of cellular transcripts.

### Determinants of RNase E in sRNA Processing

**Figure 5:**
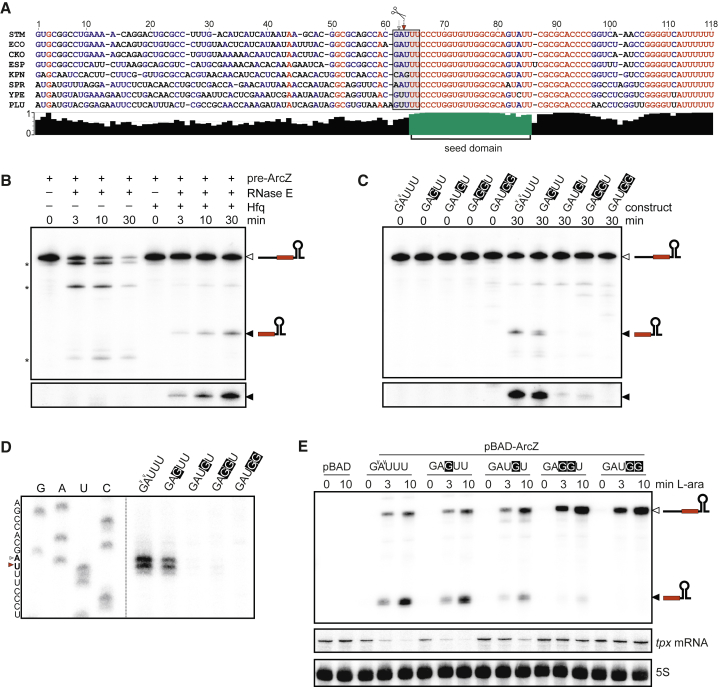
RNase E Mediates the Maturation of ArcZ sRNA In Vitro and In Vivo (A) Alignment of ArcZ sequence. Conservation scores are plotted below the sequences, and the conserved seed is colored in green. (B) Reconstitution of ArcZ maturation in vitro. Full-length pre-ArcZ RNA was incubated with RNase E in the presence or absence of Hfq. RNA was analyzed by northern blotting with an oligo antisense to the mature ArcZ. The lower set shows mature ArcZ signals with longer exposure. (C) Mutation of RNase E cleavage site. Variants of ArcZ precursors were incubated with Hfq, and then subjected to RNase E cleavage. The lower set shows mature ArcZ signals with longer exposure. (D) Primer extension to map the RNase E cleavage sites in ArcZ in vitro. (E) Validation of RNase E motif in ArcZ in vivo. See also Figures S5 and S6.

To understand how RNase E matures Hfq-associated sRNAs, we chose ArcZ for further characterization (Figure 5A). Using the purified catalytic domain (NTD) of RNase E in combination with Hfq, we could readily reconstitute in vitro the release of 3′ ArcZ (56 nt) from its 118-nt-long precursor (pre-ArcZ) prepared with T7 RNA polymerase (Figure 5B). Within 3 min, the reaction produced the mature ArcZ fragment, which accumulated over time; the cleavage occurred precisely at the expected sites identified by TIER-seq in vivo (Figure 5D). However, in the absence of Hfq, RNase E rapidly hydrolyzed pre-ArcZ into fragments without producing 3′ ArcZ (Figure 5B). This suggests that Hfq plays a role in directing the correct processing of ArcZ by RNase E.

The maturation site in ArcZ in *Salmonella* and *E. coli* matches well with our TIER-seq-derived RNase E consensus (GA↓U_+1_U_+2_U_+3_; Figure 5A versus Figure 2F), featuring uridines (U_+2_U_+3_) at the second and third position downstream of the cleavage site that are highly conserved in numerous enterobacterial species (Papenfort et al., 2009). Strikingly, changing U_+2_ to a disfavored G in the RNase E motif strongly diminished ArcZ processing by RNase E in vitro (Figures 5C and 5D), and processing was fully inhibited by further mutating U_+3_. By contrast, the same change at U_+1_ alone had little if any effect (Figures 5C and 5D). To explore if these findings have bearing on the maturation process in vivo, we expressed mutant ArcZ variants from inducible pBAD plasmids and analyzed the status of the ArcZ sRNA. Consistent with the in vitro results, the U_+2_→G_+2_ mutation strongly reduced the levels of 3′ ArcZ in *Salmonella* (Figure 5E), with further reductions upon additional mutation of the upstream (U_+1_U_+2_ → G_+1_G_+2_) and downstream (U_+2_U_+3_ → G_+2_G_+3_) uridines. Of note, the processing of ArcZ seems to be required for the regulation of its target *tpx* mRNA (see below).

The crucial roles of U_+2_ and Hfq in RNase E cleavage were also evident for the RprA sRNA (Figure S5). Full-length RprA precursor (pre-RprA) was processed by RNase E in vitro at its internal seed sequence (GA↓A_+1_U_+2_U_+3_), producing mature RprA only in the presence of Hfq. Mutating U_+2_ alone significantly reduced the maturation of RprA by RNase E, which was fully abolished by changing both U_+2_U_+3_ to non-preferred guanines. The essentiality of U_+2_ in RprA processing could also be demonstrated in vivo (Figure S5C), as well as in directing the cleavage of the *cfa* mRNA (Figure S2E). Together, these mutational studies further validate our TIER-seq-based prediction of U_+2_ as a key nucleotide for specific RNase E cleavage of cellular transcripts.

### RNase-E-Dependent sRNA Maturation Is Essential for Target Regulation

**Figure 6:**
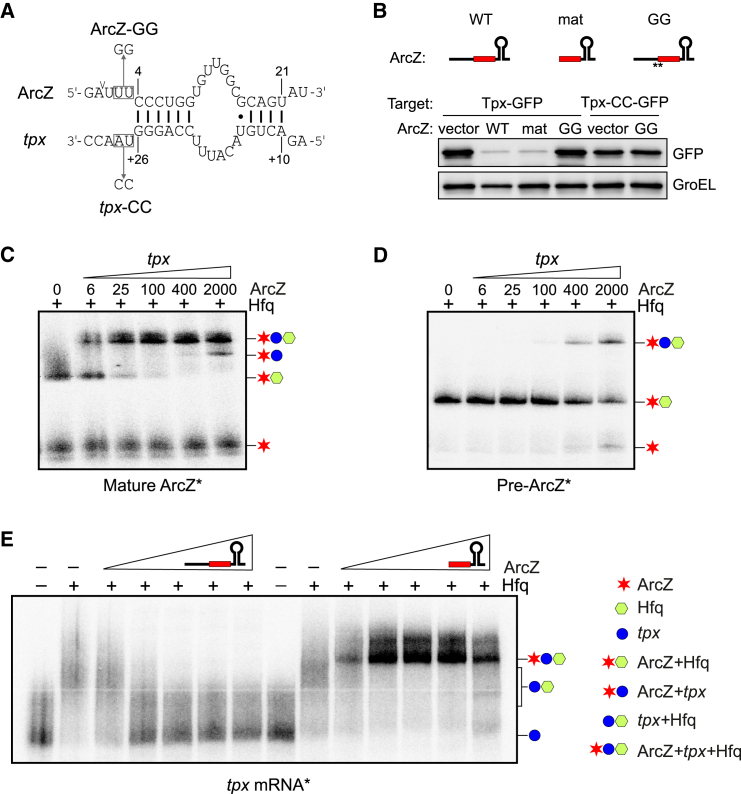
Maturation of ArcZ sRNA Is Essential for Target Regulation (A) Established base pair interactions between ArcZ and *tpx* mRNA (Papenfort et al., 2009). The major cleavage site in ArcZ is indicated. (B) Western blot detection of GFP levels. GFP was fused with *tpx* 5′ UTR; the introduced mutations are shown in (A). “WT” refers to WT full-length ArcZ, “mat” refers to mature ArcZ, and “GG” refers to the GAUGG variant of ArcZ. GroEL served as loading control. (C) Direct interaction of *tpx* with mature ArcZ by EMSA. Radiolabeled mature ArcZ was incubated with increasing concentration of *tpx* mRNA in the presence of Hfq (40 nM). The gel was resized; see Figure S6. (D) Direct interaction of *tpx* with pre-ArcZ by EMSA. (E) Mature ArcZ was co-shifted with *tpx* mRNA. Radiolabeled *tpx* mRNA was incubated with increasing concentration of pre-ArcZ or mature ArcZ (0, 6, 25, 100, 400, and 2,000 nM) in the presence of 40 nM Hfq. See also Figure S6.

To consider RNase E as an sRNA maturation factor with functional consequences requires that its processing activity is essential for sRNA function. Demonstrating such a property requires first the development of a system in which processing of an sRNA precursor can be impeded without changing or losing the seed region. The ArcZ sRNA offers such a system: exploiting our finding that mutation of the crucial U_+2_ in the RNase E motif of ArcZ abolished cleavage enabled us to produce pre-ArcZ with diminished amounts of 3′ ArcZ in vivo (Figure 5). We examined the ability of the pre-ArcZ to repress the synthesis of Tpx (Figure 5E), whose mRNA is targeted by the conserved seed region of ArcZ (Papenfort et al., 2009; Figures 6A and S6). While a 10 min expression of WT ArcZ downregulated the *tpx* mRNA by 7-fold, the U_+2_→G_+2_ mutant (variant GAUGU) achieved only 3-fold repression despite the higher levels of precursor (Figure 5E). Additional mutation of an adjacent uridine (variants GAGGU or GAUGG) fully inhibited 3′ ArcZ production and abrogated *tpx* regulation despite higher levels of the precursor, strongly suggesting that only the mature 3′ ArcZ is the functional regulator.

According to previous work (Papenfort et al., 2009), the U_+2_U_+3_ residues in the RNase E site of ArcZ may not engage in base paring with *tpx* (Figure 6A). If they do at all, they might extend the duplex by two additional A:U pairs; this could be disrupted by the non-functional, locked GAUGG variant of pre-ArcZ. To rule out that the failure of the GAUGG variant (ArcZ-GG) to repress *tpx* was simply due to insufficient base pairing, we introduced a compensatory AU→CC mutation in the *tpx-*GFP fusion (Tpx-CC), but again no regulation by the ArcZ-GG variant was observed (Figure 6B). Likewise, the processing-deficient ArcZ-GG variant also failed to regulate the *sdaC* mRNA target either in its WT form or with a duplex-extending CC mutation (Figure S6B). Thus, RNase E cleavage is essential for the production of functional ArcZ.

A likely explanation for ArcZ maturation to be essential for regulation is that the ArcZ seed may only become available for target pairing upon RNase E cleavage. To test this, we examined sRNA duplex formation with *tpx* mRNA in vitro. Electrophoretic mobility shift assays with radiolabeled sRNA showed that the mature 3′ ArcZ binds to the target region of *tpx* mRNA (a 216 nt fragment containing 5′ UTR and early CDS) with very high affinity (K_D_ ≈15 nM; Figure 6C); by contrast, an ∼ 500-fold excess of pre-ArcZ over target was insufficient for full duplex formation (Figure 6D), similar to the low affinity observed for pre-ArcZ binding to the *rpoS* mRNA (Soper et al., 2010). In addition, Hfq promotes formation of the sRNA target duplex in the case of mature ArcZ, but less so for pre-ArcZ (Figures S6C and S6D). These results were further confirmed by reciprocal experiments with labeled *tpx* mRNA. Again, mature ArcZ readily bound to the target and formed a stable ArcZ-*tpx*-Hfq ternary complex (Figure 6E), whereas excess of the pre-ArcZ RNA only competed with the *tpx*-Hfq complex formation and released free *tpx* mRNA. These combined in vivo and in vitro results show that pre-ArcZ undergoes an RNase-E-dependent maturation to activate ArcZ for repression of *tpx* and perhaps other targets. This demonstrates for the first time that RNase E cleavage is required to activate an Hfq-dependent sRNA.

## Discussion

Bacterial transcripts are generally short lived (Bernstein et al., 2002, Chen et al., 2015) and subject to rapid turnover by cellular ribonucleases (Hui et al., 2014, Mackie, 2013). Gene expression and regulation typically take place at the level of primary transcripts bearing the native 5′ PPP end. This is fundamentally different from higher eukaryotes, where nearly all types of regulatory transcripts undergo processing and maturation as a prerequisite for function. Our identification of numerous conserved regulatory sRNAs that result from RNase E cleavage (Figures 3 and S3) illustrates the complexity of the bacterial “RNA degradome.” These increasing numbers of processing-derived RNA species (Chao and Vogel, 2016, Davis and Waldor, 2007, Deltcheva et al., 2011, Guo et al., 2014, Miyakoshi et al., 2015a) contrast with the general perception that cleaved bacterial transcripts are usually labile species of little biological relevance.

TIER-seq offers a generic approach both for global analysis of processed transcripts and cleavage sites in living cells with single-nucleotide resolution and for mechanistic understanding of ribonuclease activities at a systems level. We have here employed a temperature-sensitive strain to transiently inactivate the endogenous RNase E, which minimizes the potentially confounding effects of “non-native” conditions used in previous degradome studies where the nucleases were genetically deleted (Linder et al., 2014), ectopically overexpressed (Schifano et al., 2014), or supplemented in vitro (Clarke et al., 2014). To circumvent the need for a thermosensitive mutant and temperature-induced transcriptomic changes (Table S7), future TIER-seq studies may benefit from using alternative means of transient nuclease inactivation such as small molecules (Kime et al., 2015), small inhibitory proteins (Kim et al., 2008, Lee et al., 2003), target-specific proteases (Cameron and Collins, 2014), or conditionally spliced inteins (Zeidler et al., 2004).

### RNase-E-Dependent sRNA Biogenesis and Maturation in Bacteria

**Figure 7:**
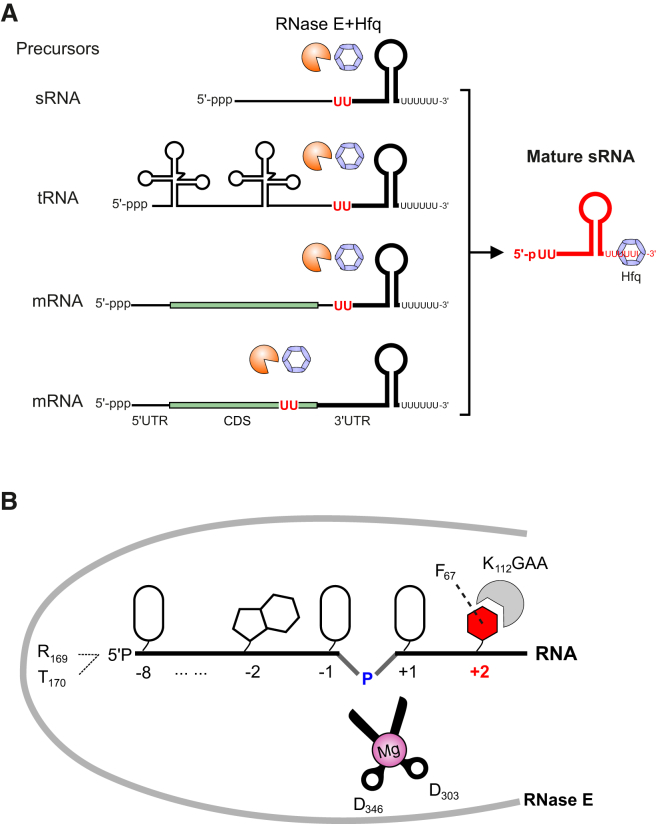
Mechanism of RNase E Cleavage and an Alternative sRNA Biogenesis Pathway (A) RNase E cleavage constitutes a major sRNA biogenesis pathway in bacteria. (B) Proposed model for the +2 uridine ruler-and-cut mechanism of specific RNase E cleavage. The scissile phosphate is attacked hydrolytically by a water molecule (not shown) that is coordinated by the magnesium ion bound by the carboxylates of D_346_ and D_303_. Stacking interactions (between F_67_ and K_112_) and hydrogen bonding (with the K_112_GAA loop) with the base at position +2 favor uridine at this position. The interactions are predicted to help orient the phosphate backbone into a geometry that would facilitate cleavage at the scissile phosphate. See also Figure S7.

We identify RNase E as a key factor both for the biogenesis of many 3′ UTR-derived sRNAs and for the maturation of active sRNAs from their non-coding precursors. This establishes RNase E cleavage as a second major pathway for the biogenesis of Hfq-dependent sRNAs (Figure 7A). As compared to the canonical pathway of de novo transcription, this cleavage-based biogenesis may confer several advantages. RNase E can generate sRNAs from diverse origins, including essentially all existing transcripts (Figure 7A), greatly expanding the sRNA repertoire in the cell. This pathway could reduce regulatory overhead during evolution of new genes (Mattick, 2004), using the existing regulatory elements of the parental transcripts to control the expression of 3′-derived sRNAs. Activating an internal seed sequence by sRNA precursor cleavage, as shown here for ArcZ, offers an additional layer of control in post-transcriptional regulation—for example, via an adaptor protein such as RapZ, which facilitates specific RNase E cleavage in certain sRNAs (Göpel et al., 2013). Lastly, RNase-E-cleavage-derived sRNAs carry a 5′ P end which promotes mRNA target degradation (Bandyra et al., 2012, Chao and Vogel, 2016, Pfeiffer et al., 2009) and, as a consequence, different regulation kinetics than translational control alone (Levine and Hwa, 2008).

The key role of RNase E in sRNA biogenesis mirrors the central role of this enzyme in mRNA target regulation by many Hfq-dependent sRNAs (Massé et al., 2003, Saramago et al., 2014, Vogel and Luisi, 2011). Importantly, target degradation was proposed to involve tripartite RNase-E-based ribonucleoprotein complexes with sRNA and Hfq (Ikeda et al., 2011, Morita et al., 2005, Worrall et al., 2008). Our results indicate that this complex may form in order to mediate the alternative biogenesis of sRNAs prior to their target decay. For example, an ArcZ-Hfq-RNase E complex must form in the course of ArcZ maturation from the Hfq-bound, pre-ArcZ sRNA. In this respect, the Hfq-RNase E complex in bacteria could have a dual function: it processes precursor transcripts to stable, mature sRNA and guides the mature sRNA for target regulation.

### A U_+2_ Ruler-and-Cut Mechanism Mediates Specific RNase E Cleavage

The hallmark of the RNase E consensus motif inferred from our in vivo map (Figure 2) is a predominant uridine at 2 nt downstream of the cleavage sites (U_+2_), and we provide in vivo and in vitro evidence that the U_+2_ is crucial for RNase E cleavage. Analysis of the available crystal structure of an RNase E-RNA complex shows that the enzyme interacts with RNA at +2 nt via a stable stacking interaction of the nucleobase with Phe_67_ and Lys_112_ (Callaghan et al., 2005, Mackie, 2013). However, this structure contains a non-cognate substrate with G_+2_, representing a stable RNA-binding conformation trapped at the pre-cleavage state. Why is a uridine at this position preferred for cleavage? A molecular dynamics simulation analysis in which G_+2_ is substituted for U in silico suggests that the RNase E-RNA complex undergoes a conformational change favored by the presence of U_+2_; this allows us to propose a new model (Figure 7B) whereby RNase E mediates specific cleavage using a U_+2_ ruler-and-cut mechanism. Simulations of the pre-cleavage state show that U_+2_ was tightly bound in a crevice of the protein formed by the backbone of the Lys_112_Gly_113_Ala_114_Ala_115_ loop and the Lys_112_ side chain, resulting in a binding pocket that favors uracil (uracil pocket, Figures S7A–S7C and supplemental discussion). Importantly, the presence of the cognate U_+2_ promotes a distortion of the phosphodiester backbone angles at the cleavage site 2 nt upstream. The new conformation of the scissile phosphate may closely resemble, with slight deviation, the pseudo-trigonal, bipyramidal geometry that facilitates in-line nucleophilic attack of scissile phosphate (Figures 7B and S7D–S7F). While we have shown here that mutating U_+2_ in RNA abolishes cleavage, mutation of the critical Lys_112_ also abrogates RNase E cleavage of cognate substrates (Callaghan et al., 2005). The high conservation of residues forming the uracil pocket (e.g., Phe_67_ and Lys_112_) indicates that this may be a conserved mechanism for the RNase E protein family.

The uridine ruler-and-cut mechanism is also employed by other endoribonucleases, including the unrelated human nuclease RNase L. RNase L recognizes uridine in single-stranded RNAs and cleaves 2 nt downstream (Han et al., 2014), whereas RNase E cuts 2 nt upstream due to different dimeric structure arrangements. Interestingly, a fraction of RNase E sites contain C_+2_ (Figure 2F), indicating that RNase E displays a certain degree of flexibility by accepting a cytosine in the absence of other specificity signals. Indeed, in vitro experiments with poly(A) RNA demonstrate that C_+2_ can serve as a cleavage signal (Kaberdin, 2003), which further suggests that RNase E may distinguish the smaller pyrimidine from purine bases by steric hindrance (Figures 7B, S7A, and S7B). Nevertheless, U_+2_ is the preferred signal (Figure 2F), likely because its C_4_ oxygen possesses hydrogen bonding potential with RNase E (Figure S7B). In addition, some flexibility of RNase E is reflected near the cleavage sites, as RNase E frequently cuts 1 nt upstream or downstream of the determined cleavage site. To compensate for this, short stretches of uridines (1–4 U) are often found at the +2 positions, which may serve to reinforce RNase E recognition and cleavage (e.g., ArcZ; Figure 5).

Our identification of crucial U_+2_ residues for RNase-E-specific cleavage enables straightforward mutations of individual cleavage sites of interest instead of global inactivation of the enzyme. This will aid the molecular investigation of 3′ UTR-derived sRNAs and of RNase-E-mediated, post-transcriptional regulations, not only in the Hfq regulon but also for the recently discovered class of ProQ-associated sRNAs (Smirnov et al., 2016)—many of which might be RNase E targets, too. This information may also help design novel CRISPR-Cas or antisense-RNA-based synthetic tools to activate gene expression by specifically blocking a cleavage site, as well as helping to engineer stable mRNAs for better gene expression.

## Experimental Procedures

Full methods are described in the Supplemental Experimental Procedures; so are details of bacterial strains, plasmids, and oligonucleotides.

### Transient Inactivation of RNase E

The *Salmonella rne*^TS^ strains refer to *rne*-3071 and its isogenic WT control previously established in (Figueroa-Bossi et al., 2009). Bacteria were grown in Lennox LB medium at 28°C to an OD_600_ of 2, then shifted to 44°C for 30 min to inactivate RNase E.

### RNA-Seq and Data Analysis

cDNA libraries were constructed following a standard protocol (Chao et al., 2012, Westermann et al., 2016). Briefly, RNA was polyadenylated at 3′ end and ligated to an adaptor at 5′ end after treatment with tobacco acid pyrophosphatase. First-strand cDNA was synthesized using oligo(dT)-adaptor and M-MLV reverse transcriptase. The linear amplified cDNAs were multi-plexed and sequenced using Illumina HiSeq. Reads were mapped to *Salmonella* genome using READemption; 5′ end coverage was visualized in IGB. The RNase E sites, which are depleted 5′ ends in the *rne*^TS^ samples relative to WT at 44°C, were identified using DESeq2.

## Author Contributions

Y.C. and J.V. conceived the research; Y.C., N.S., C.C., M.S., and K.P. conducted experiments; Y.C., L.L., K.U.F., and B.F.L. analyzed data; D.G. and H.-J.W. performed MD simulations; R.R. performed RNA-seq; Y.C., B.F.L., and J.V. wrote the manuscript.

## Accession Numbers

The sequencing data have been deposited in the GEO database under GEO: GSE81869.

## Supplemental Information

Document S1. Supplemental Experimental Procedures and Figures S1–S7

Table S1. List of the Identified RNase E Cleavage Sites and Sequences: Related to Figure 1 and Figure 2

Table S2. The RPKM Values of Genes Expressed at 44°C: Related to Figure 1

Table S3. Essential Genes and Virulence-Associated Genes Containing RNase E Cleavage Sites: Related to Figure 2

Table S4. Genes with Cleavage Sites in the 5′ UTR: Related to Figure 2

Table S5. List of 3′UTR-sRNA Candidates: Related to Figure 3

Table S6. Small RNAs Containing RNase E Cleavage Sites: Related to Figure 2 and Figure 3

Table S7. Temperature-Upshift-Induced Changes in WT Samples: Related to Figure 1

Document S2. Article plus Supplemental Information
