# Supplementary material for: In Vivo Cleavage Map Illuminates the Central Role of RNase E in Coding and Non-coding RNA Pathways
Source: Mol Cell. 2017 Jan 5;65(1):39–51. doi: 10.1016/j.molcel.2016.11.002 (PMC5222698; doi:10.1016/j.molcel.2016.11.002)
Supplement: Document S1. Supplemental Experimental Procedures and Figures S1–S7 [file mmc1.pdf]

Molecular Cell, Volume *65*

Supplemental Information

In Vivo Cleavage Map Illuminates the Central Role of RNase E in Coding and Non-coding RNA Pathways

Yanjie Chao, Lei Li, Dylan Girodat, Konrad U. Förstner, Nelly Said, Colin Corcoran, Michał Śmiga, Kai Papenfort, Richard Reinhardt, Hans-Joachim Wieden, Ben F. Luisi, and Jörg Vogel

Supplemental discussion on molecular dynamics simulation

The identified cleavage sites for sRNA biogenesis suggest that RNase E recognizes its target site via a consensus motif (Fig. 2F) with strong selection for a uridine two nucleotides downstream of the cleavage site (U_{+2}). X-ray crystallographic structures of RNase E bound to a non-cognate substrate revealed that the base two nucleotides downstream of the cleavage site stacks with amino acid residues Phe_{67} and Lys_{112} of RNase E (Fig. S7A; (Callaghan et al., 2005; Mackie, 2013). The fact that the scissile phosphate is located two nucleotides upstream of the conserved uridine in the recognition motif suggests that RNase E recognizes the cleavage site using a two-nucleotide (U_{+2}) ruler mechanism. This two-nucleotide ruler mechanism is facilitated via the correct positioning of a water molecule (coordinated by an enzyme-bound magnesium ion) for attack on the phosphodiester bond at this position. However, the available structures do not provide information regarding the mechanism leading to uridine recognition. This is in part due to the fact that the available RNase E structures contain a non-cognate RNA trapped at pre-cleavage state (Callaghan et al., 2005). Based on this we hypothesized that upon binding to a cognate, cleavable RNA substrate, RNase E may undergo a conformational rearrangement of its catalytic site that requires the presence of a uridine two nucleotides downstream of the scissile phosphate. To investigate this, we carried out all-atom molecular dynamics simulations exploring the structural dynamics of RNase E in the presence of a cognate (U_{+2}) and a non-cognate (G_{+2}) substrate. Our simulations reveal that the cognate U_{+2} not only stacks with Phe_{67}, but it is also tightly bound in a putative uracil binding pocket consisting of the $Lys_{112}Gly_{113}Ala_{114}Ala_{115}$ ($^{112}KGAA^{115}$) loop in the S1 domain of RNase E (Fig. S7A-B). The peptide-backbone amides of this loop are oriented towards the U_{+2} forming a positively charged cleft where Ala_{114} and Gly_{113} can form hydrogen bonds to the O^4 of the U_{+2} base, ultimately providing a feature that allows to discriminate between uracil and guanine bases.

Interestingly, during the course of the simulations we observed a repositioning of the Lys_{112} side chain which only occurs in the presence of the cognate U_{+2} substrate (Fig. S7 A-C). In our simulation of the RNase E-cognate RNA complex, Lys_{112} adopts two distinct conformations that are different from the previously proposed position (Callaghan et al., 2005). In these conformations the side chain of Lys_{112} forms a transient interaction with the phosphate one nucleotide downstream (23% of the time within 4 Å of a phosphate oxygen) and a more stable conformation interacting with Phe_{76} (75% of the time within 4 Å of Phe_{67}), essentially closing the uracil binding pocket and trapping the U_{+2}. It is not surprising that this conformation has not previously been observed in the available non-cognate structures as the non-cognate G_{+2} base, contains an additional bulky imidazole ring that sterically prevents the sampling of this conformation by Lys_{112}. Constant with this, Lys_{112} is not resolved in the available X-ray crystallographic structures and is likely to be highly flexible in the non-cognate complex (Callaghan et al., 2005). The closed uracil pocket in conjunction with the reposition of Lys_{112} shift the position of A_{+1} and U_{+2} nucleotide bases leading to a distortion of the phosphodiester backbone (Fig. S7D & Fig. 7B). The distortion at the scissile phosphate likely proceeds through a pseudo trigonal bipyramidal geometry (or 'five-oxygen interacting state') optimal for the in-line nucleophilic attack of an activated water molecule mediated by divalent metal ions (Martick and Scott, 2006; Oivanen et al., 1998; Torres and Bruice, 1998). This geometry is not formed in the simulation with RNA alone (Fig. S7F), or with RNase E-RNAs complex containing the non-cognate G_{+2} in RNA (Fig. S7E), as the closing of the uracil pocket via the Lys_{112} is not possible due to the larger guanine base. The subsequent cleavage reaction will result in fragments with the phosphate

on the 5' end and a fragment with the OH group on the 3' end (Fig. S7D), consistent with the well-established features of RNase E cleavage (Mackie, 2013; Misra and Apirion, 1979).

Overall, our data suggest that RNase E is able to specifically recognize a uracil in substrate RNA and mediates cleavage using a U_{+2} ruler and cut mechanism (Fig. 7B & Fig. S7D). Notably, the residues critical for forming the uracil pocket are conserved in all RNase E homologs. The alanine substitution of the Lys_{112} has previously been shown to abolish cleavage activity also for cognate substrates, suggesting that the proposed mechanism is conserved and required for RNase E activity (Callaghan et al., 2005). This is further supported by previous *in vitro* observations, which show that substrate RNAs with G_{+2} can bind to RNase E but are not cleaved (Callaghan et al., 2005; Kaberdin, 2003).

Figure S1

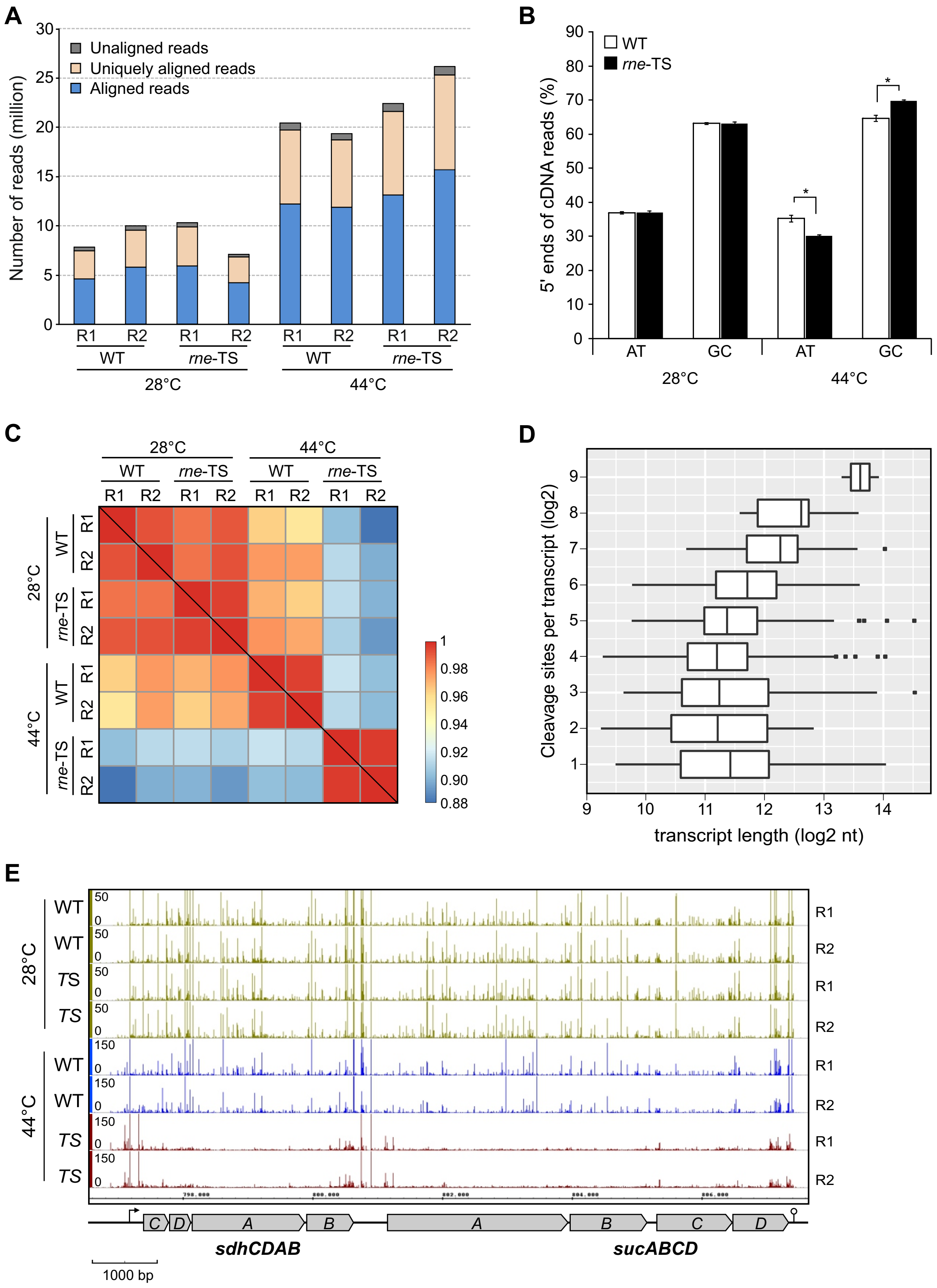

A
Unaligned reads
Uniquely aligned reads
Aligned reads
Number of reads (million)
R1 R2 R1 R2 R1 R2 R1 R2
WT rne-TS WT rne-TS
28°C 44°C
B
WT
rne-TS
5' ends of cDNA reads (%)
AT GC AT GC
28°C 44°C
C
28°C 44°C
WT rne-TS WT rne-TS
R1 R2 R1 R2 R1 R2 R1 R2
1 0.98 0.96 0.94 0.92 0.90 0.88
D
Cleavage sites per transcript (log2)
transcript length (log2 nt)
E
28°C WT WT TS TS
44°C WT WT TS TS
R1 R2 R1 R2 R1 R2 R1 R2
C D A B A B C D
sdhCDAB
sucABCD
1000 bp

Figure S2

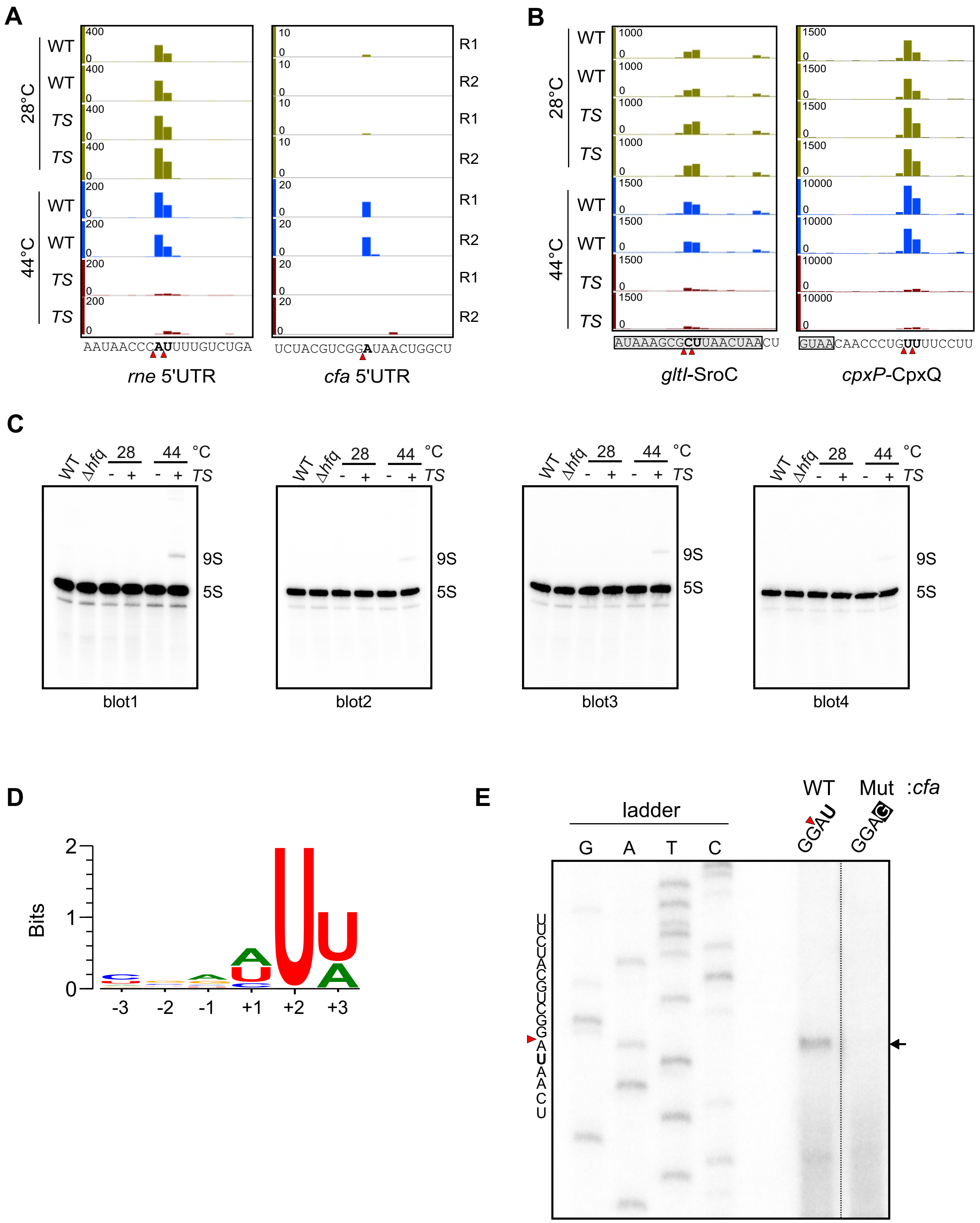
A
28°C
44°C
WT
TS
R1
R2
AAUAAACCCAUUUUGUCUGA
UCUACGUCGGAUAACUGGCU
rne 5'UTR
cfa 5'UTR
B
AUAAAGCGCUUAACUAACU
GUAACAACCCUGUUUUCCUU
gltI-SroC
cpxP-CpxQ
C
WT
Δhfq
28
44
°C
TS
9S
5S
blot1
blot2
blot3
blot4
D
Bits
-3
-2
-1
+1
+2
+3
E
ladder
G
A
T
C
WT
Mut
:cfa
GGAU
GGAG
UUCUACGUCGGAUAACU

Figure S3

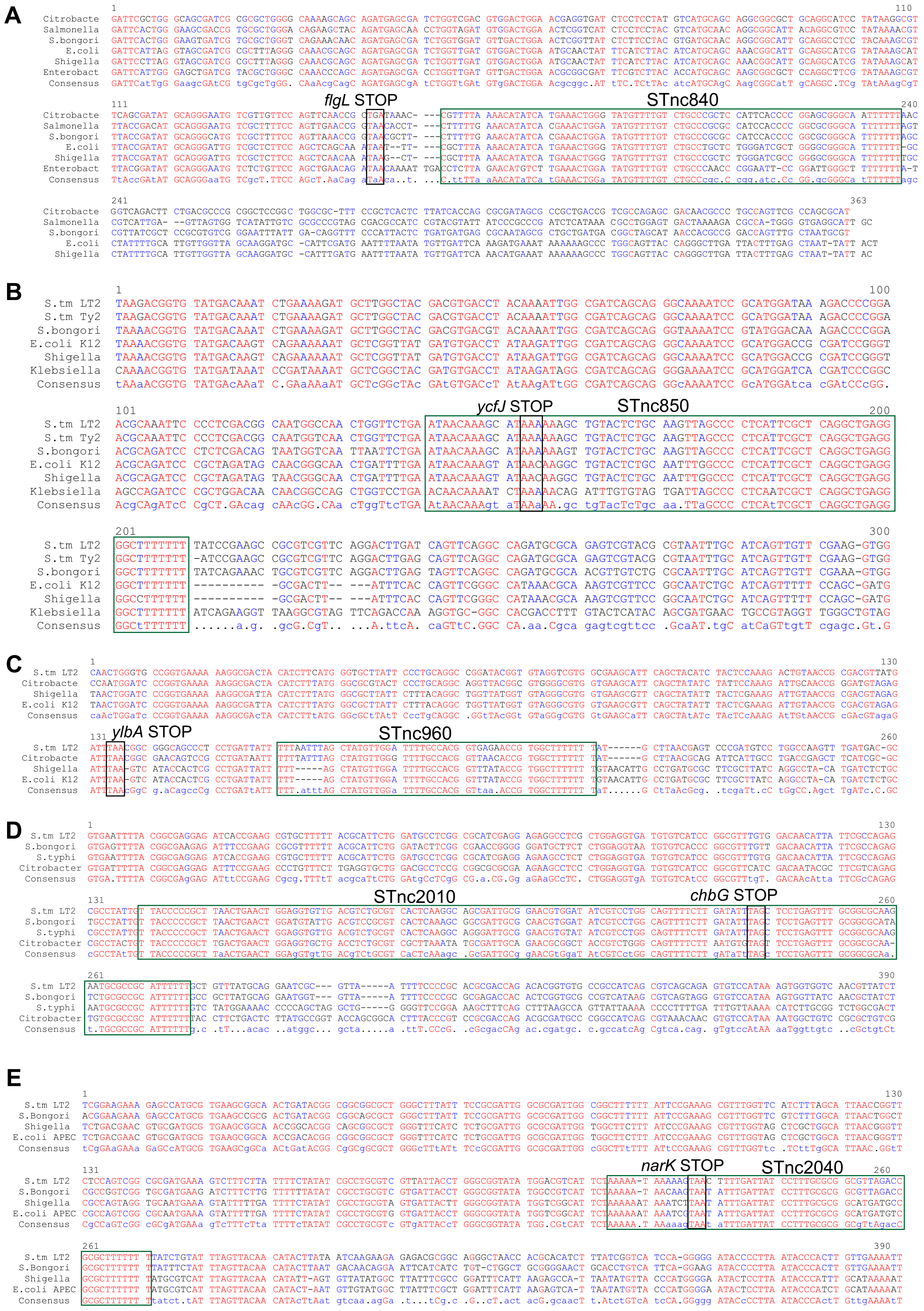

A
Citrobacte
Salmonella
S.bongori
E.coli
Shigella
Enterobact
Consensus
flgL STOP
STnc840
B
S.tm LT2
S.tm Ty2
S.bongori
E.coli K12
Shigella
Klebsiella
Consensus
ycfJ STOP
STnc850
C
S.tm LT2
Citrobacte
Shigella
E.coli K12
Consensus
ylbA STOP
STnc960
D
S.tm LT2
S.bongori
S.typhi
Citrobacter
Consensus
STnc2010
chbG STOP
E
S.tm LT2
S.Bongori
Shigella
E.coli APEC
Consensus
narK STOP
STnc2040

Figure S4

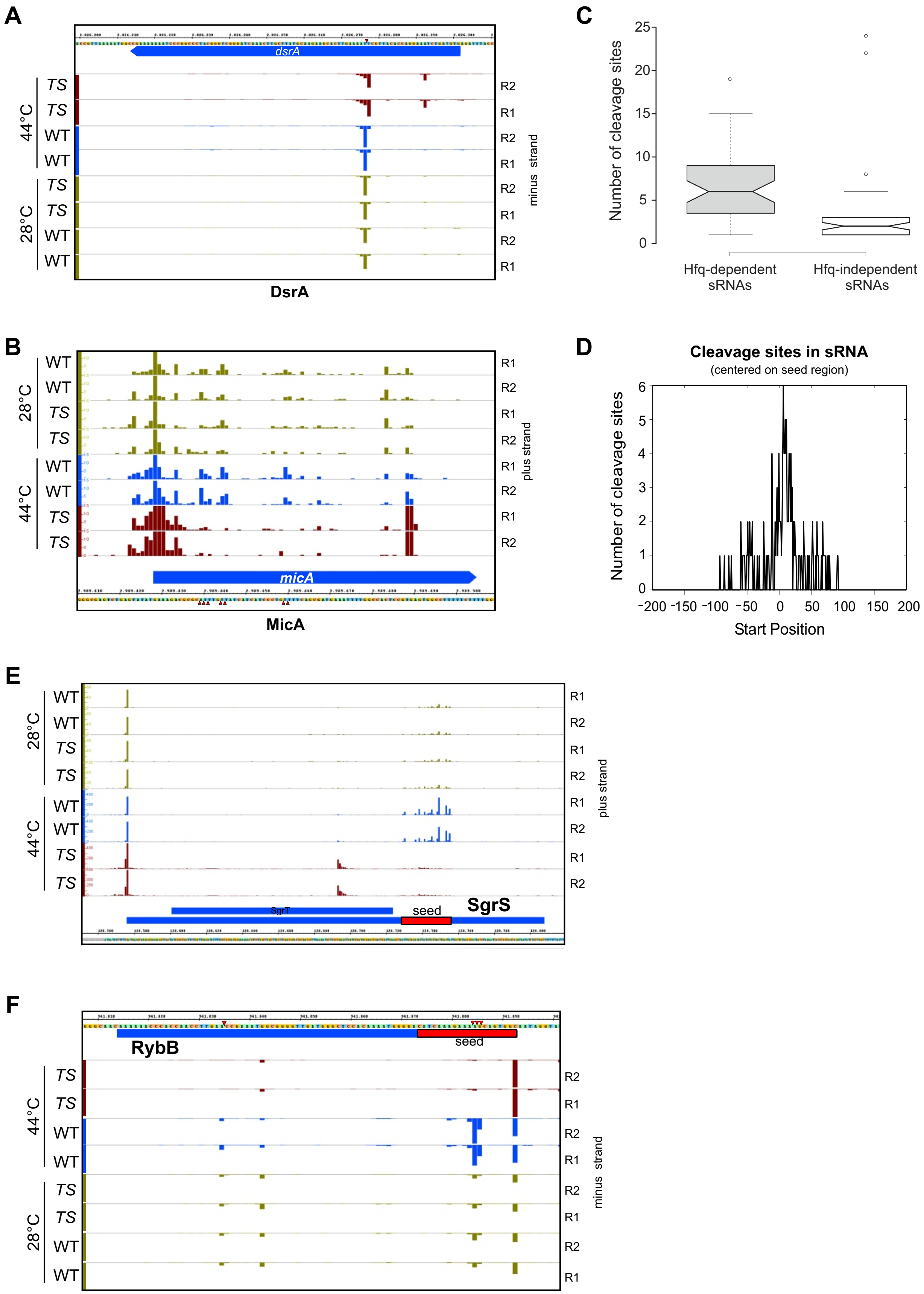

A
dsrA
TS
TS
WT
WT
TS
TS
WT
WT
44°C
28°C
R2
R1
R2
R1
R2
R1
R2
R1
minus strand
DsrA
B
WT
WT
TS
TS
WT
WT
TS
TS
28°C
44°C
R1
R2
R1
R2
R1
R2
R1
R2
plus strand
micA
MicA
C
Number of cleavage sites
25
20
15
10
5
0
Hfq-dependent sRNAs
Hfq-independent sRNAs
D
Cleavage sites in sRNA
(centered on seed region)
Number of cleavage sites
6
5
4
3
2
1
0
-200 -150 -100 -50 0 50 100 150 200
Start Position
E
WT
WT
TS
TS
WT
WT
TS
TS
28°C
44°C
R1
R2
R1
R2
R1
R2
R1
R2
plus strand
SgrT
seed
SgrS
F
RybB
seed
TS
TS
WT
WT
TS
TS
WT
WT
44°C
28°C
R2
R1
R2
R1
R2
R1
R2
R1
minus strand

Figure S5

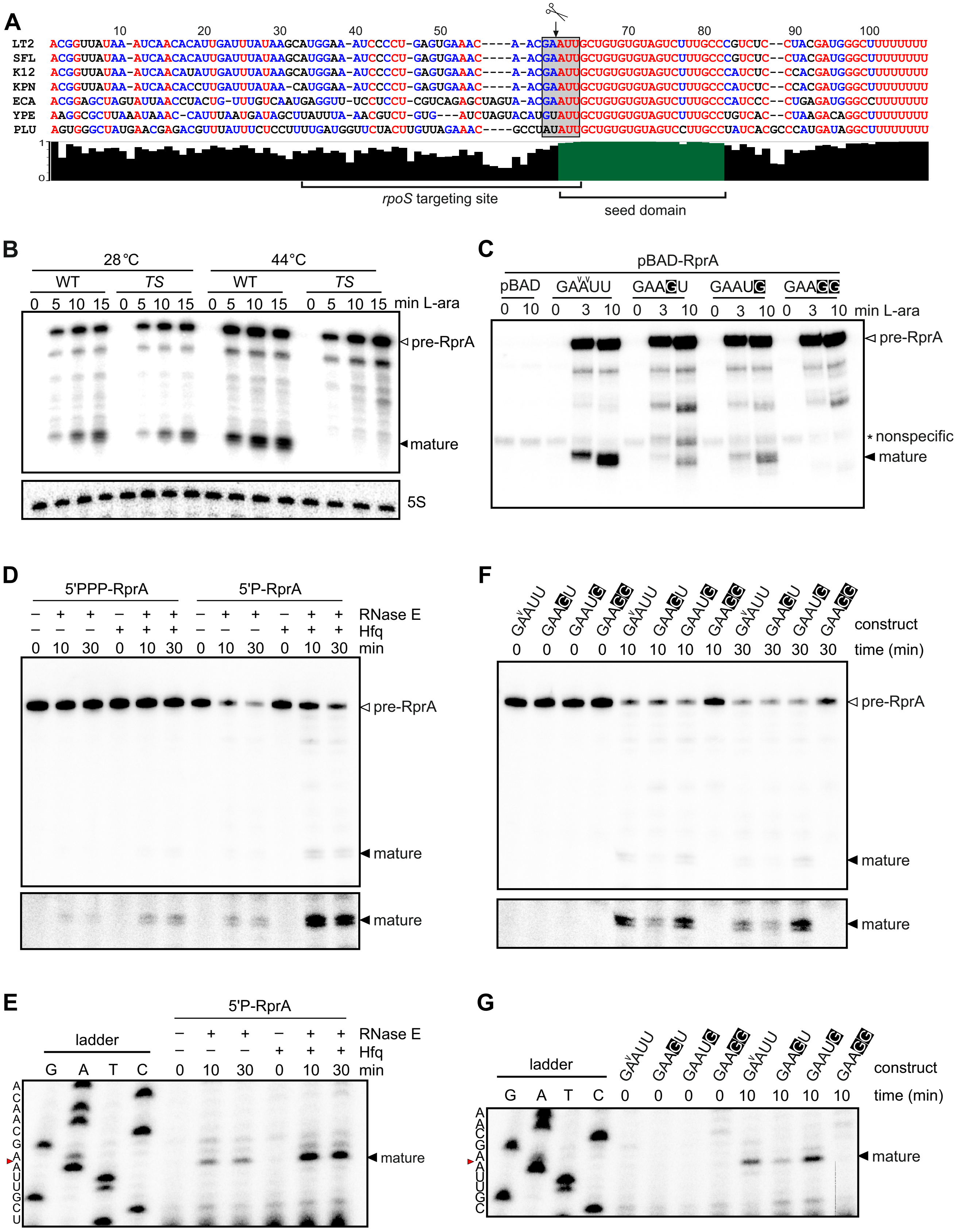

A
10 20 30 40 50 70 80 90 100
LT2 ACGGUUAUAA-AUCAACACAUUGAUUUAUAAGCAUGGAA-AUCCCCU-GAGUGAAAC----A-ACGAAUUGCUGUGUGUAGUCUUUGCCCGUCUC--CUACGAUGGGCUUUUUUUU
SFL ACGGUUAUAA-AUCAACACAUUGAUUUAUAAGCAUGGAA-AUCCCCU-GAGUGAAAC----A-ACGAAUUGCUGUGUGUAGUCUUUGCCCGUCUC--CUACGAUGGGCUUUUUUUU
K12 ACGGUUAUAA-AUCAACAUAUUGAUUUAUAAGCAUGGAA-AUCCCCU-GAGUGAAAC----A-ACGAAUUGCUGUGUGUAGUCUUUGCCCAUCUC--CCACGAUGGGCUUUUUUUU
KPN ACGGUUAUAA-AUCAACACCUUGAUUUAUAA-CAUGGAA-AUCCCCU-GAGUGAAAC----A-ACGAAUUGCUGUGUGUAGUCUUUGCCCAUCUC--CCACGAUGGGCUUUUUUUU
ECA ACGGAGCUAGUAUUAACCUACUG-UUUGUCAAUGAGGUU-UCCUCCU-CGUCAGAGCUAGUA-ACGAAUUGCUGUGUGUAGUCUUUGCCCAUCCC--CUGAGAUGGGCCUUUUUUU
YPE AAGGCGCUUAAAUAAAC-CAUUUAAUGAUAGCUUAUUUA-AACGUCU-GUG---AUCUAGUACAUGUAUUGCUGUGUGUAGUCUUUGCCUGUCAC--CUAAGACAGGCUUUUUUUU
PLU AGUGGGCUAUGAACGAGACGUUUAUUUCUCCUUUUGAUGGUUCUACUUGUUAGAAAC----GCCUAUAUUGCUGUGUGUAGUCCUUGCCUAUCACGCCCAUGAUAGGCUUUUUUUU
1
0
rpoS targeting site
seed domain
B
28 °C
44°C
WT
TS
WT
TS
0 5 10 15 0 5 10 15 0 5 10 15 0 5 10 15 min L-ara
pre-RprA
mature
5S
C
pBAD-RprA
pBAD
GAAUU
GAACU
GAAUC
GAACC
0 10 0 3 10 0 3 10 0 3 10 0 3 10 min L-ara
pre-RprA
* nonspecific
mature
D
5'PPP-RprA
5'P-RprA
– + + – + + – + + – + + RNase E
– – – + + + – – – + + + Hfq
0 10 30 0 10 30 0 10 30 0 10 30 min
pre-RprA
mature
mature
F
GAAUU
GAACU
GAAUC
GAACC
GAAUU
GAACU
GAAUC
GAACC
GAAUU
GAACU
GAAUC
GAACC
construct
0 0 0 0 10 10 10 10 30 30 30 30 time (min)
pre-RprA
mature
mature
E
ladder
5'P-RprA
– + + – + + RNase E
– – – + + + Hfq
G A T C 0 10 30 0 10 30 min
A
C
A
A
C
G
A
A
U
U
G
C
U
mature
G
ladder
GAAUU
GAACU
GAAUC
GAACC
GAAUU
GAACU
GAAUC
GAACC
construct
G A T C 0 0 0 0 10 10 10 10 time (min)
A
A
C
G
A
A
U
U
G
C
mature

Figure S6

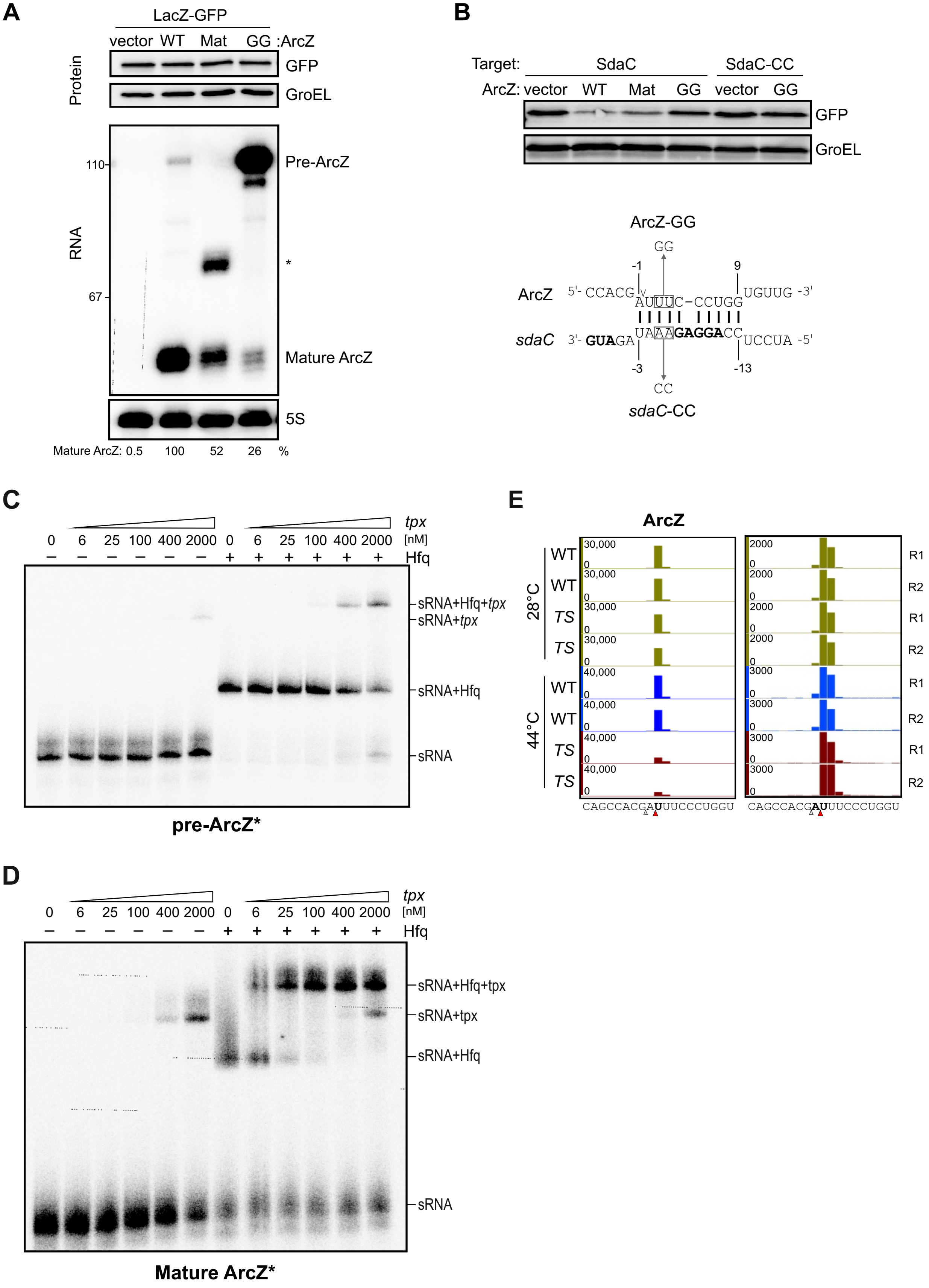

A
LacZ-GFP
vector WT Mat GG :ArcZ
Protein
GFP
GroEL
RNA
110
67
Pre-ArcZ
*
Mature ArcZ
5S
Mature ArcZ: 0.5 100 52 26 %
B
Target: SdaC SdaC-CC
ArcZ: vector WT Mat GG vector GG
GFP
GroEL
ArcZ-GG
GG
-1
9
ArcZ 5'- CCACG AUUUC-CCUGG UGUUG -3'
sdaC 3'- GUAGA UAAAGAGGACC UCCUA -5'
-3
-13
CC
sdaC-CC
C
tpx
0 6 25 100 400 2000 0 6 25 100 400 2000 [nM]
Hfq
sRNA+Hfq+tpx
sRNA+tpx
sRNA+Hfq
sRNA
pre-ArcZ*
D
tpx
0 6 25 100 400 2000 0 6 25 100 400 2000 [nM]
Hfq
sRNA+Hfq+tpx
sRNA+tpx
sRNA+Hfq
sRNA
Mature ArcZ*
E
ArcZ
28°C
44°C
WT
TS
R1
R2
30,000
40,000
2000
3000
0
CAGCCACGAUUUCCCUGGU

Figure S7

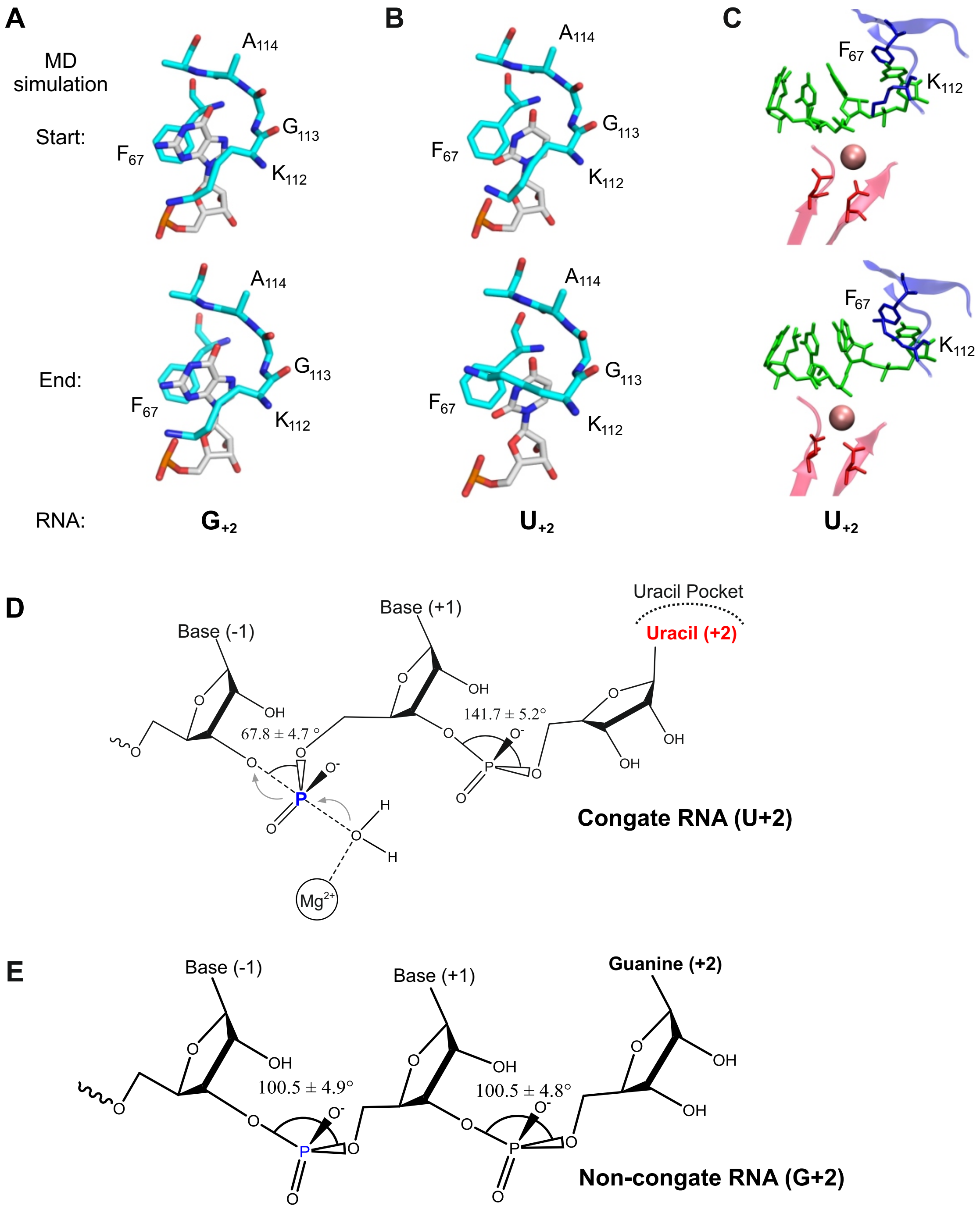

F

	Cognate U_{+2}	Non-Cognate G_{+2}	Cognate U_{+2} (RNA Alone)
O5' – P – O3'	67.8 ± 4.7°	100.5 ± 4.9°	100.6 ± 4.8°
O5' – P – OP1	110.1 ± 5.4°	109.4 ± 5.2°	109.5 ± 5.2°
O5' – P – OP2	109.6 ± 5.5°	111.4 ± 5.2°	109.4°± 5.3
O3' – P – OP1	120.7 ± 4.4°	109.1 ± 5.3°	110.1 ± 4.9°
O3' – P – OP2	120.0 ± 4.9°	110.4 ± 4.9°	110.0 ± 5.2°
OP1 – P – OP2	115.1 ± 4.6°	114.6 ± 5.1°	115.4 ± 5.1°

Supplemental Figure Legends:

Figure S1. Overview of TIER-seq results. Related to Figure 1 and Figure 2.

A) Bar plot showing the total number of raw cDNA reads (million) obtained for all the samples. R1 and R2 are two biological replicates. The unaligned reads are shown in grey; the rest reads have been aligned to the *Salmonella* genome. Among these aligned sequences, uniquely aligned reads (mostly non-rRNA/tRNA reads) are indicated in light orange.

B) The inactivation of RNase E leads to a reduction of cDNA reads with 5' A/T base. Bars indicate the relative amount (%) of reads that have A/T or G/C bases at the 5' end in a cDNA library. The error bars indicate standard deviation. * indicates $p<0.05$, two-tailed Student's *t*-test.

C) Samples are clustered based on the similarity of 5′ ends profile. The reads counts of all the detected 5′ end positions in a cDNA library are compared with that of another cDNA library. The Pearson correlation coefficient is plotted, and the scale bar is shown.

D) The number of cleavage sites per transcript was plotted against the respective gene (transcript) length.

E) The reads distribution in the polycistronic *sdh-suc* operons showing numerous RNase E cleavage-derived 5' ends. IGB screenshot is shown; R1 and R2 indicate two biological replicates.

Figure S2. Examples of mapped RNase E cleavage sites. Related to Figure 1, Figure 2 and Figure 3.

A) The mapped RNase E cleavage sites in the *rne* 5'UTR and the *cfa* 5'UTR, which match the previously determined sites. We note that the RNase E cleavage in *cfa* may be affected by temperature, as more cleavage products were detected at the elevated temperature (44°C).

B) The mapped RNase E cleavage sites in the 3'UTR of *cpxP* and *gltI*, which are responsible for the biogenesis of 3'UTR-derived CpxQ and SroC sRNA, respectively. The shaded box indicates the open reading frame of the respective host gene (*cpxP*, *gltI*). The arrowheads show the mapped cleavage site, or the 5' end of the resulting sRNAs.

C) 5S rRNA loading controls for the northern blots shown in the Fig. 3 and Fig. 4. Note that the 9S rRNA signals confirms the transient inactivation of RNase E at 44˚C (rne-TS).

D) RNase E sequence logo derived from all the cleavage sites in 3'UTR-derived sRNAs.

E) Mutation of U_{+2} abolishes RNase E cleavage in mRNA. The RNase E cleavage sites in the 5' UTR of *cfa* mRNA was determined using primer extension. WT *cfa* or mutant *cfa* was constitutively expressed from pKF31 or pYC311. Total RNA was isolated and subjected to primer extension analysis using 5' end-labeled oligo pZE-XbaI. The determined RNase E cleavage site is indicated by arrow.

Figure S3. Sequence alignment of new 3'UTR-derived sRNAs identified in this study. Related to Figure 3.

A) Sequence alignment of STnc840 in the *flgL* 3'UTR. The green box indicates sRNA sequence and the black box indicates the stop codon of its host mRNA.

B) Sequence alignment of STnc850 in the *ycfJ* 3'UTR.
C) Sequence alignment of STnc960 in the *ylbA* 3'UTR.
D) Sequence alignment of STnc2010 in the *chbG*/*celG* 3'UTR.
E) Sequence alignment of STnc2040 in the *narK* 3'UTR.

Figure S4. Analysis of RNase E cleavage sites in Hfq-dependent sRNAs. Related to Figure 3.

A) Screenshot showing all the 5' ends of reads within *dsrA* locus. The arrowhead indicates the mapped RNase E site. Data from both replicates are shown.
B) Screenshot showing all the 5'ends within *micA* locus. The arrowheads indicate the mapped RNase E sites. Data from both replicates are shown.
C) The number of identified cleavage sites in Hfq-dependent sRNAs are higher than that in Hfq-independent sRNAs. Center lines show the medians; box limits indicate the 25th and 75th percentiles; whiskers extend 1.5 times the interquartile range from the 25th and 75th percentiles, outliers are represented by dots.
D) Meat-gene analysis showing the distribution of cleavage sites within sRNAs. The position on the x-aixs is relative to the seed of sRNAs.
E) Screenshot showing all the 5' ends within *sgrS* locus. The defined seed region of SgrS is highlighted. Data from both replicates are shown.
F) Screenshot showing all the 5' ends within *rybB* locus. The defined seed region in the 5' end of RybB is highlighted, and the mapped RNase E cleavage sites are indicated by arrow heads. Data from both replicates are shown.

Figure S5. RNase E mediates the maturation of RprA small RNA. Related to Figure 4 and Figure 5.

A) Sequence alignment of the RprA small RNA. The RNase E cleavage site is indicated by a scissor. The sequence conservation score is shown below the alignment. LT2: S*almonella* Typhimurium LT2. SFL: *Shigella flexneri*. K12: *Escherichia coli* K-12. KPN: *Klebsiella pneumoniae*. ECA: *Erwinia carotovora*. YPE, *Yersinia pestis*. PLU: *Photorhabdus luminescens*.
B) The processing of RprA is dependent on RNase E. In both rne^{TS} (TS) and WT control strains, the RprA sRNA was pulse-expressed from pKP15-13 by the addition of 0.2% L-arabinose for 0, 5, 10, or 15 min. RprA was detected by northern blotting using 5'end-labeled oligo JVO-4049. 5S served as a loading control.
C) Mutations at U_{+2} abolishes RprA maturation in vivo. WT RprA and its variants were induced from pBAD plasmids by the addition of 0.2% L-arabinose for 0, 3, or 10 min. RprA was detected by northern blotting using 5'end-labeled oligo JVO-4049.
D) RprA was processed by RNase E in the presence of Hfq in vitro. The RprA sRNA with either 5'PPP or 5'P (TAP treated) was incubated with equimolar ratio of RNase E in the presence or absence of Hfq in vitro. The reactions were stopped at the indicated time point and separated by a denaturing 6% PAA/7M urea gel. RprA was detected by northern blotting using 5'end-labeled oligo JVO-4049.

E) Mapping the *in vitro* cleavage sites by primer extension using RNA from (D). The arrowhead indicates the RNase E cleavage sites in RprA determined by primer extension using 5'end-labeled oligo JVO-5902.

F) The processing of RprA is dependent on the critical uridine at +2 position. The RprA variants (with 5'P) were incubated with Hfq and RNase E for the indicated time period. The reactions were separated by a denatured 6% PAA/7M urea gel, and RprA was detected using 5'end-labeled oligo JVO-4049. The mature RprA fragment was shown in the lower panel with increased exposure.

G) Mapping the in vitro cleavage sites using primer extension. RNA from (F) was subjected to primer extension analysis using 5'end-labeled oligo JVO-5902. The arrowhead indicates the determined RNase E cleavage sites in RprA.

Figure S6. The maturation of ArcZ is required for target binding and regulation. Related to Figure 6.

A) Expression of ArcZ variants *in vivo*. *Salmonella* Δ*arcZ* strain (JVS-0082) containing LacZ-*gfp* control plasmid (pXG-1) and the sRNA plasmid vector (pKP8-35), full-ArcZ (pKP4-13), short-ArcZ (pKP31-5), or ArcZ* (pYC250) were grown to OD_{600} of 2.0 in LB medium (~3h) in the presence of 0.2% L-arabinose. The expression of GFP was analyzed by Western blotting and the expression of ArcZ sRNA was analyzed by northern blotting using 5'-end labeled oligo JVO-4208. The quantification of the mature ArcZ level is shown below (%).

B) Regulation of SdaC translational fusions. The expression of SdaC-GFP reporter (pKP-102) and SdaC*-GFP reporter (pYC348) was analyzed as in (A). The quantification of GFP levels is shown (%). The introduced mutations and the base pairing interactions are shown to the right (Papenfort et al., 2009).

C) EMSA analysis of the interactions between mature ArcZ sRNA and *tpx* mRNA. ~5 nM 5'-end-labeled mature wt ArcZ sRNA was incubated with increasing concentrations of the unlabeled *tpx* mRNA fragment (0, 6, 25, 100, 400, 2000 nM) at 37°C for 10 min, in the absence (-) or presence (+) of 40 nM Hfq.

D) EMSA analysis of the interactions between pre-ArcZ sRNA and *tpx* mRNA. ~5 nM 5'-end-labeled pre-ArcZ sRNA was incubated with increasing concentration of the unlabeled *tpx* mRNA fragment (0, 6, 25, 100, 400, 2000 nM) at 37°C for 10 min, in the absence (-) or presence (+) of 40 nM Hfq.

E) IGB screenshot showing the major (red arrowhead) and minor (open arrowhead) RNase E cleavage sites mapped in ArcZ.

Figure 7. Molecular Dynamics simulation reveals conformation changes during the RNase E-cognate RNA recognition facilitating cleavage of substrate RNA. Related to Figure 7.

A) Non-cognate RNA sequence bound to RNase E. At the start of the simulation, Lys_{112} coordinates both the +1 and scissile phosphates, and this conformation is maintained over the course of the simulation. RNA is colored in white, and the RNase E protein residues are colored in cyan. (top) RNA backbone at the beginning of the simulation, (bottom) RNA backbone at the end of the simulation.

B, C) Cognate RNA sequence bound to RNase E. Lys_{112} coordinates the +1 and scissile phosphates at the start of the simulation similar to (A). Over the course of the simulation, Lys_{112} adopts a conformation in which it has additional stacking potential with Phe_{67}. This conformation allows stacking of the Lys_{112} side chain with Phe_{67} and the cognate U_{+2} base present in the uracil pocket (C), closing the pocket around the base. Pink sphere, Mg^{2+} ion. (top) RNA backbone at the beginning of the simulation, (bottom) RNA backbone at the end of the simulation.

D) The conformation of RNA with cognate RNA with U_{+2}, revealed by molecular dynamics simulations. Upon recognition of the U_{+2} in the substrate RNA, the conformation of RNA-backbone 2 nt upstream of uridine is predicted to become distorted in a manner that may favor approach of the scissile phosphate to a pseudo-trigonal bipyramidal transition state geometry (90º) for in-line attack of a water molecule, consistent with the U_{+2} ruler mechanism.

E) The conformation of RNA with non-cognate G_{+2}. Different from the cognate RNA with U_{+2}, this geometry is maintained throughout the course of simulation, representing a stable bound state of RNA in the RNase E crystal (Callaghan et al., 2005).

F) Geometry of the Scissile Phosphate. Only the cognate RNA bound to RNase E adopts the distorted O5'-P-O3' angle that facilitates the in-line attack of a water molecule. The results are derived from three independent replicates, and the respective standard deviation is shown.

Supplemental Tables:

Table S1. List of the identified RNase E cleavage sites and sequences. Related to Figure 1 and Figure 2.

Table S2. The RPKM values of genes expressed at 44°C. Related to Figure 1.

Table S3. Essential genes and virulence-associated genes containing RNase E cleavage sites. Related to Figure 2.

Table S4. Genes with cleavage sites in the 5'UTR. Related to Figure 2.

Table S5. List of 3'UTR-sRNA candidates. Related to Figure 3.

Table S6. Small RNAs containing RNase E cleavage sites. Related to Figure 2 and Figure 3.

Table S7. Temperature upshift induced changes in WT samples. Related to Figure 1.

See the EXCEL files.

Supplemental Experimental Procedures

Bacterial strains and growth

The bacterial strains, plasmids, oligonucleotides used in this study are included in the supplemental lists. *Salmonella enterica* strains JVS-7000 ((*rluC-rne*)IG:*cat*/*rne*-3071, MA9816) and JVS-6999 (*rluC-rne*)IG:*cat*, MA9292) from the Bossi lab (Figueroa-Bossi et al., 2009) were used as the rne^{TS} and its isogenic WT strains. Bacteria were grown in LB-Lennox medium to an OD_{6oo} of 2.0 at 28°C with 220 rpm agitation, and were shifted to 44°C in a water bath and continue to grow for 30 min prior to RNA extraction. Experiments with other bacterial strains were performed at 37°C with 220 rpm agitation. A final concentration of 0.02% of L-arabinose was used to induce sRNA expression from pBAD vectors.

Total RNA extraction

Bacterial cultures corresponding to ~4 OD_{600} were collected and mixed with 0.2 vol. ice-cold STOP solution (95 % Ethanol, 5 % Phenol). Total RNA was isolated following the hot phenol protocol in order to preserve the RNA integrity. In brief, bacterial cells were resuspended in 600 µl of lysozyme solution (0.5 mg/ml, pH 8.0) and 60 µl of 10% SDS, and then incubated at 64°C for 1-2 minutes to lyse cells. After adding 66 µl of 3 M NaOAc (pH 5.2), the cell lysis was extracted by 750 µl phenol (Carl-Roth, #A980.3) and followed by another extraction using 750 µl chloroform. The supernatant was collected and mixed with 3 vol. ice-cold ethanol to precipitate total RNA at -20°C for 2 hours or overnight. RNA was treated with 5 units of DNase I (Fermentas) at 37°C for 45 min to remove residue DNA contamination. To isolate RNA from in vitro assays, samples were extracted by phenol:chloroform:isoamylaclcohol (P:C:I, 25:24:1, Carl-Roth, #A156.3), and RNA was precipitated using 3 vol. ice-cold ethanol at -20°C for >2h or at -80°C for 30 min.

Northern blotting

5 or 10 µg total RNA was denatured in 1× loading buffer II at 95°C for 3 min, and separated by 6% polyacrylamide (PAA) gel with 7 M urea for 2 h at 300 V. RNA was transferred to Hybond-XL membranes (GE Healthcare) by electro-blotting (1 h, 50 V, 4°C) in 1× TBE buffer. After crosslinking by 0.12 J/cm^2 UV light, membranes were hybridized with 5'-end radiolabeled DNA oligos at 42°C overnight. To radiolabel the DNA oligos, 10 pmol oligo was incubated with 25 µCi of [γ-32P]-ATP and 1 U T4 polynucleotide kinase (PNK) at 37°C for 1 h. The unincorporated ATP was removed using MicroSpin G-25 columns (GE healthcare) according to the manufacturer's instruction. The oligonucleotides sequences can be found in the Supplemental list of DNA oligonucleotides. 5S rRNA were probed using JVO-0322 as the loading control. Signal was visualized on a phosphorimager (Typhoon FLA 7000, GE Healthcare) and quantified using the AIDA image analyzer (Raytest).

In vitro RNA transcription and RNase E assays

RNA fragments were generated from DNA templates by T7 *in vitro* transcription using MEGAscript T7 kit (Life technologies) following the manufacturer's instructions. The DNA templates were prepared by PCR using gene-specific oligos containing T7 promoter sequences (see Supplemental list of DNA oligonucleotides). *In vitro*

transcription was carried out with ~200 ng DNA template at 37°C for ~6 h or at room temperature overnight. 5'P-RNAs are generated by incubating 5 µg *in vitro* transcribed RNA with 5 units tobacco alkaline phosphatase (TAP, Epicenter) at 37°C for 1 h.

RNase E assays were performed using an equimolar concentration (300 nM) of precursor RNAs, Hfq and the catalytic domain of RNase E (amino acids 1-529, NTD) in a 10 µl volume reaction, according to the established protocol (Bandyra et al., 2012; Chao and Vogel, 2016). Purified RNase E-NTD protein was provided by KJ Bandyra and BF Luisi at University of Cambridge. Prior to assays, *in vitro* transcribed RNA fragment was denatured for 1 min at 95°C, chilled on ice for 5 min. RNA was then mixed with the reaction buffer (25 mM Tris pH 7.5, 50 mM NaCl, 50 mM KCl, 10 mM $MgCl_2$, 1 mM DTT) in a 10 µl reaction volume. If necessary, the Hfq protein was added to the reaction, and incubated at 30°C for 10 min to allow Hfq-RNA complex formation. RNase E protein was then added and incubated at 30°C. Reactions were either directly loaded to denaturing PAA/7M urea gels for northern blotting analysis, or stopped by adding 100 µl of stop buffer (50 mM Tris-HCl, 0.1 % SDS and 10 mM EDTA) on ice. Following a P:C :I (25:24:1) extraction, purified RNA was used for primer extension analysis or was analyzed by northern blotting using the 5'-end-radiolabeled oligos.

Primer extension

Primer extension experiments were performed with half of the RNA extracted from *in vitro* assays. After a brief denaturation of RNA at 95°C and 5 min chilling on ice, 5 µl of the reaction mix (3 µl 5× First strand buffer, 0.5 mM each dATP, dGTP, dCTP and dTTP, and 5 mM DTT) was supplemented with 1 µl SuperScript III reverse transcriptase (Invitrogen, Thermo Fisher Scientific). Reverse transcription was carried out at 50°C for 60 min, and stopped by an incubation at 70°C for 15 min. After removal of DNA template by a digestion with 2.5 U RNase H at 37 °C for 15 min, samples were mixed with 1× loading buffer II and loaded to 6% PAA gel with a sequencing apparatus. The reference sequence ladder was prepared using the DNA fragment amplified by PCR using gene-specific oligos (Supplemental list of DNA oligonucleotides), and subjected to sequencing reaction using the DNA cycle sequencing kit (Jena Bioscience) following the manufacturer's instructions.

Electrophoretic Mobility Shift Assay (EMSA)

To prepare 5'-end-labeled RNAs for EMSA, 20 pmol in vitro synthesized RNA was first dephosphorylated at 37°C for 1 h using 10 U Calf Intestinal Alkaline Phosphates (CIP, New England Biolabs) and purified by P:C:I extraction and ethanol precipitation. The purified RNA was then 5' phosphorylated at 37°C for 1 h using 1 U T4 polynucleotide kinase (PNK, Fermentas) and 20 µCi [γ-32P]-ATP. Following a purification with MicroSpin G-25 columns to remove unincorporated ATP, labelled RNA was separated by denaturing PAGE, excised from gels and eluted by soaking gel pieces in RNA elution buffer at 4 to 8°C overnight.

EMSA assays were then performed with a 5'-end-labeled RNA and an unlabeled RNA in the presence or absence of the Hfq protein. One RNA fragment of interest was 5'-end-labeled, and denatured at 95°C for 2 min and chilled on ice for 5 min. For each 10 µl volume reaction, ~5 nM RNA was supplemented with 1× structure buffer, 1 µg yeast RNA, and various amount of unlabeled partner RNA. The reactions were incubated at 37°C for 10 min, then mixed with 3 µl native loading buffer (50 % glycerol, 0.5× TBE and 0.2 % bromphenol blue) and directly loaded to a

6% native PAGE gel running with cold 0.5× TBE buffer (4°C). Radiography signal was determined using a Typhoon FLA7000 phosphorimager and the AIDA software.

Western blotting

Bacterial samples were resuspended in 1× protein loading buffer and boilded at 95°C for 5 min. Equal amount of total protein samples (equivalent to ~0.05 OD cells) were loaded to each lane and separated by SDS-PAGE. Protein samples were transferred to a polyvinylidene fluoride (PVDF) membrane, and incubated with antibodies against GFP epitope or GroEL (Sigma) for 1 hour at room temperate or overnight at 4°C. After incubation with appropriate secondary antibodies conjugated with HRP, chemiluminescence signals were developed and analyzed with an ImageQuant LAS 4000 CCD imager (GE Healthcare) and the AIDA software (Raytest).

RNA Sequencing and data processing

For deep sequencing analysis, total RNAs were converted to cDNA libraries at Vertis Biotechnologie AG (Munich, Germany) following a standard protocol described previously (Chao et al., 2012; Westermann et al., 2016). In brief, total RNA was poly(A)-tailed using poly(A) polymerase, and the 5'PPP structure was removed using tobacco acid pyrophosphatase (TAP, epicenter). An RNA adaptor was ligated to the 5'P of the RNA. First-strand cDNA synthesis was performed using an oligo(dT)-adapter primer and the M-MLV reverse transcriptase. The resulting cDNAs were PCR-amplified to about 10-20 ng/μl using a high fidelity DNA polymerase, and purified using the Agencourt AMPure XP kit (Beckman Coulter Genomics). The cDNAs were multiplexed and sequenced using the TruSeq chemistry and Illumina HiSeq platform.

After an initial run with all samples, the samples from 44°C were re-sequenced to gain greater depth. The sequencing data from two runs were combined prior to downstream analysis. Reads with a Phred score lower than 20 were trimmed by the program fastq_quality_trimmer (FASTX toolkit), and reads shorter than 12 nt were removed using a size filter. The remaining reads were mapped to *Salmonella* Typhimurium SL1344 reference genome (NC_016810.1) and three associated plasmids (NC_017718.1, NC_017719.1, NC_017720.1) using segemehl (version 0.2.0) (Hoffmann et al., 2009) and READemption (version 0.3.5) with a mapping accuracy cutoff of 95% (Forstner et al., 2014). The 5' end of unique aligned reads was extracted from mapped BAM files using samtools (Li et al., 2009). The sequence composition of 5' nucleotide was calculated by the program fastacomposition (version 2.2.0). Pair-wise Pearson correlations were performed on 5' read coverage of all cDNA libraries. The coverage plots for all mapped 5' ends were generated by READemption with the 'first_base_only' option and stored in the wiggle format. IGB genome browser (http://bioviz.org/igb) was used to visualize the coverage plots and to generate the screenshot figures (Nicol et al., 2009). The raw reads as well as normalized coverage files have been deposited in NCBI GEO database with accession number GSE81869. Note that data from the initial run were used to analyze temperature effect in wild-type samples (Supplemental Table S7), because of the similar library sizes between 28°C and 44°C samples.

Identification of RNase E cleavage sites

The 5' end profile (number of reads per nucleotide) was compared between *rne*^{TS} and WT samples using DESeq2 with default options (v1.8.1) (Love et al., 2014); and the RNase E cleavage sites are identified by comparing the 5' end profile of WT at 44°C to that of the *rne*^{TS} samples at 44°C. The 5' ends that show >3-fold decrease (depletion) in the

rne^{TS} samples and false discovery rate (<0.05) corrected *P*-value lower than 0.05, were considered significantly different. To facilitate differential expression analyses, a pseucount of 0.0001 was added for the nucleotide with zero read count. MA plot was created to display the distribution between log fold change and mean expression, and the significant 5' ends were shown using python. To avoid putative false-positives, candidate cleavage sites are filtered by TEX-resistant positions inferred from dRNA-Seq data (unpublished), which led to a removal of 357 positions. The final set of 22,033 RNase E cleavage sites were the 5' ends that display >3-fold decrease (depletion) in the *rne*^{TS} samples at 44°C and statistically significant (FDR-adjusted *P* value<0.05).

Analysis of sequence features of cleavage sites

For the analyses of RNase E sites distribution, the genomic annotation of SL1344 was used and extended by custom annotations of sRNAs and UTRs. The annotations of CDS, tRNA and rRNA was collected from NCBI *Salmonella* Typhimurium SL1344 genome database (NC_016810.gff). The list of *Salmonella* essential genes is derived from a previously determined dataset (Barquist et al., 2013). The annotation of 5'UTRs was based on the primary transcription start sites (pTSS) determined previously (Kröger et al., 2013; Kröger et al., 2012). And the annotation of 3'UTRs was based on the position of downstream Rho-independent terminators, which were predicted by RNIE (Gardner et al., 2011). The cleavage sites located within these annotated features were identified using bedtools (Quinlan, 2014; Quinlan and Hall, 2010). In the absence of an upstream TSS, an arbitrary 100 nt 5'UTR was added upstream of the CDS, and similarly an arbitrary 100 nt 3'UTR was added in the absence of a terminator. The transcript length considers the distance from the pTSS to the predicated terminator. The sRNA annotations are based on previously published dataset (Chao et al., 2012; Chinni et al., 2010; Kröger et al., 2013; Kröger et al., 2012; Perkins et al., 2009) as well as unpublished annotations.

To identify the sequence and structural features of RNase E cleavage sites, sequences upstream and downstream of the identified cleavage sites were extracted. The consensus motif was identified using the MEME suite (v 4.9.1) with default parameters (Bailey et al., 2009; Bailey et al., 2015). The RNase E consensus motif logo was generated using all the sequences derived from 22,033 cleavage sites by Weblogo3 webserver (http://weblogo.threeplusone.com) (Crooks et al., 2004).

To evaluate the structural properties around RNase E cleavage sites, the secondary structure of RNA fragment in a sliding window (size: 25 nt, 5 nt/step) was predicted and the minimum free energy (MFE) was calculated using the RNAfold algorithm from the ViennaRNA Package (Lorenz et al., 2011). The MEF of the sequence in a window surrounding the cleavage sites was compared to the distribution of 1000 random shuffled sequences with the same dinucleotide composition, and a *Z*-score was calculated and used for illustration.

For meta-gene analysis, the density of cleavage sites was calculated by counting the number of sites along the specified annotation features described above, e.g. the start codon and the stop codon of respective genes. The number of cleavage sites were summarized based on the relative distance to either start or stop codon for genes, or based on the start of the established 'Seed' domains for 21 known Hfq-binding sRNAs. The theoretical distribution of RNase E cleavage sites was analyzed by counting the number of predicted cleavage motifs positioned in *Salmonella* genome; and the rate of occurrence at each position relative to the start or stop codon was calculated.

3'UTR-derieved sRNA candidates

To identify candidate 3'UTR-derived sRNAs, the genes that have at least one identified cleavage sites near the last nucleotide of stop codon (50 bp upstream or downstream) were extracted. The Hfq-association profile of these genes at 3'UTR was analyzed as well based on the Hfq-coIP data under the same growth condition (OD_{600} of 2.0) published previously (Chao et al, 2012). The 3'UTRs that possess a predicted Rho-independent terminator and show >3 fold enrichment in Hfq-coIP compared to WT-coIP were selected as sRNA candidates, and listed in the Supplemental Table S5.

Molecular Dynamics

Structure-based heavy atom (non-hydrogen) Gō models were designed based on the crystal structure of *E. coli* RNase E-RNA complex (PDB ID: 2C0B) (Callaghan et al., 2005) and the respective topology and parameter files were generated using the SMOG server (Noel et al., 2010). To reduce the size of the system and in turn the computational cost of the simulations we used the functional dimer of RNase E (Callaghan et al., 2005). Two separate models were constructed using this method. The first was RNase E containing a non-cognate 10-mer RNA sequence derived directly from the crystal structure (5'-ACAGUAUUUG-3'), the second RNase E model contained an RNA 10-mer with the cognate sequence of (5'-ACAGUAGUAU-3') derived from our analysis. To this end we altered the RNA sequence in the respective model using the psfgen package in the Visual Molecular Dynamics (VMD) suite (Humphrey et al., 1996). The models were simulated using native contact parameters previously described in (Whitford et al., 2009).All simulations were performed using reduced units in the GROMACS v 5.0.4 software package (Hess et al., 2008; Lindahl et al., 2001; Van Der Spoel et al., 2005) utilizing force fields available from the SMOG server (Whitford et al., 2009). Each simulation was performed using a time step of 0.0005 for a total of 20 million time-steps. Phosphate angles and bond distances were measured using the 'g_angle' and 'g_dist' packages as implemented in GROMACS v 5.0.4 (Hess et al. 2008). To assess the conformations accessible to the Lys_{112} side chain in the cognate and non-cognate complex, two additional simulations with the native contacts constrains for Lys_{112} removed were performed using the method described above.

T7-RNA sequences

>WT ArcZ:

GUGCGGCCUGAAAACAGGACUGCGCCUUUGACAUCAUCAUAAUAAGCACGGCGCAGCCACGAUUUCCCUGGUGUUGGC
GCAGUAUUCGCGCACCCCGGUCAAACCGGGGUCAUUUUU

>ArcZ G+1:

GUGCGGCCUGAAAACAGGACUGCGCCUUUGACAUCAUCAUAAUAAGCACGGCGCAGCCACGAGUUCCCUGGUGUUGGC
GCAGUAUUCGCGCACCCCGGUCAAACCGGGGUCAUUUUU

> ArcZ G+2:

GUGCGGCCUGAAAACAGGACUGCGCCUUUGACAUCAUCAUAAUAAGCACGGCGCAGCCACGAUGUCCCUGGUGUUGGC
GCAGUAUUCGCGCACCCCGGUCAAACCGGGGUCAUUUUU

>ArcZ G+2G+3:

GUGCGGCCUGAAAACAGGACUGCGCCUUUGACAUCAUCAUAAUAAGCACGGCGCAGCCACGAUGGCCCUGGUGUUGGC
GCAGUAUUCGCGCACCCCGGUCAAACCGGGGUCAUUUUU

>Mature ArcZ:

UUUCCCUGGUGUUGGCGCAGUAUUCGCGCACCCCGGUCAAACCGGGGUCAUUUUU

>WT-RprA:

GACGGUUAUAAAUCAACACAUUGAUUUAUAAGCAUGGAAAUCCCCUGAGUGAAACAACGAAUUGCUGUGUGUAGUCUU
UGCCCGUCUCCUACGAUGGGCUUUUU

>RprA G+2:

GACGGUUAUAAAUCAACACAUUGAUUUAUAAGCAUGGAAAUCCCCUGAGUGAAACAACGAAGUGCUGUGUGUAGUCUU
UGCCCGUCUCCUACGAUGGGCUUUUU

>RprA G+3:

GACGGUUAUAAAUCAACACAUUGAUUUAUAAGCAUGGAAAUCCCCUGAGUGAAACAACGAAUGGCUGUGUGUAGUCUU
UGCCCGUCUCCUACGAUGGGCUUUUU

>RprA G+2G+3:

GACGGUUAUAAAUCAACACAUUGAUUUAUAAGCAUGGAAAUCCCCUGAGUGAAACAACGAAGGGCUGUGUGUAGUCUU
UGCCCGUCUCCUACGAUGGGCUUUUU

>Mature RprA:

AUUGCUGUGUGUAGUCUUUGCCCGUCUCCUACGAUGGGCUUUUU

>*tpx* 5’ fragment

GACGUUAACUAUAAGUAAAUAGGAACAUAAUUAUGUCACAGACUGUACAUUUCCAGGGUAACCCGGUCACCGUUGCCA
ACGUUAUUCCGCAGGCUGGUAGCAAAGCACAGGCUUUUACUCUUGUCGCAAAAGAUUUGUCUGACGUUUCCCUCAGCC
AAUAUGCAGGCAAACGCAAAGUGCUGAAUAUUUUCCCAAGCAUUGAUACUGGCGUAUGCG

Supplemental list of bacterial strains used in this study.

**Trivial name**	**Strain**	**Relevant genotype/ markers**	**Reference**
Salmonella enterica serovar Typhimurium			
Wild-type	SL1344 (JVS-1574)	*Str*^{R} *hisG rpsL xyl*	(Hoiseth and Stocker, 1981), provided by D. Bumann, MPI-IB Berlin
Δ*hfq*	JVS-0584	Δ*hfq*::FRT	(Sittka et al., 2007)
WT	JVS-6999	(*rluC-rne*) IG::*cat*	(Figueroa-Bossi et al., 2009)
rne^{TS}	JVS-7000	(*rluC-rne*) IG::*cat*/ *rne*-3071 (ts)	(Figueroa-Bossi et al., 2009)
Δ*arcZ*	JVS-0082	Δ*arcZ*::Kan^R	(Papenfort et al., 2008)
Δ*rprA*	JVS-0088	Δ*rprA*::Kan^R	(Papenfort et al., 2008)
Δ*arcZ*/ WT	JVS-11411	(*rluC-rne*) IG::*cat*/ Δ*arcZ*::Kan^R	This study
Δ*arcZ*/ *rne*^{TS}	JVS-11412	(*rluC-rne*) IG::*cat*/ *rne*-3071 (ts)/ Δ*arcZ*::Kan^R	This study
Δ*rprA*/ WT	JVS-11413	(*rluC-rne*) IG::*cat*/ Δ*rprA*::Kan^R	This study
Δ*rprA*/ *rne*^{TS}	JVS-11414	(*rluC-rne*) IG::*cat*/ *rne*-3071 (ts)/ Δ*rprA*::Kan^R	This study
Escherichia coli			
	TOP10 F′	F´{*lacI*q Tn10 (Tet^R) *mcrA* Δ(*mrr-hsd*RMS-*mcrBC*) Φ80*lacZ*ΔM15 Δ*lacX*74 *recA*1 *araD*139 Δ(*ara-leu*)7697 *galU galK rpsL endA*1 *nupG*	Invitrogen

Supplemental list of plasmids used in this study.

Stock name	Relevant fragment	Construction and Comment	Origin /marker	Reference
pKP-8-35		pBAD control plasmid, expresses the same ~50 nt nonsense RNA.	pBR322, Amp^R	(Papenfort et al., 2006)
pKP4-13	pBAD-ArcZ	WT full-length ArcZ under the control of the inducible pBAD promoter.	pBR322, Amp^R	(Papenfort et al., 2009)
pKP15-13	pBAD-RprA	WT full-length RprA under the control of the inducible pBAD promoter.	pBR322, Amp^R	(Papenfort et al., 2015)
pKP31-5		WT mature-ArcZ under the control of the inducible pBAD promoter.	pBR322, Amp^R	(Papenfort et al., 2009)
pYC247-2		pKP4-13 derived plasmid with *arcZ* T_{+1}/G mutation. pKP4-13 was re-amplified by PCR with JVO-12432/ -12433 and self-ligated.	pBR322, Amp^R	This study
pYC248		pKP4-13 derived plasmid with *arcZ* T_{+2}/G mutation. pKP4-13 was re-amplified with JVO-12432/-12434 and self-ligated.	pBR322, Amp^R	This study
pYC249-2		pKP4-13 derived plasmid with *arcZ* $T_{+1}T_{+2}$/GG mutation. pKP4-13 was re-amplified with JVO-12432/-12435 and self-ligated.	pBR322, Amp^R	This study
pYC250-2		pKP4-13 derived plasmid with *arcZ* $T_{+2}T_{+3}$/GG mutation. pKP4-13 was re-amplified with JVO-12432/-12436 and self-ligated.	pBR322, Amp^R	This study
pYC306		pKP15-13 derived plasmid with *rprA* T_{+2}/G mutation. pKP15-13 was re-amplified by JVO-13641/-9457 and self-ligated.	pBR322, Amp^R	This study
pYC307		pKP15-13 derived plasmid with *rprA* T_{+3}/G mutation. pKP15-13 was re-amplified by JVO-13642/-9457 and self-ligated.	pBR322, Amp^R	This study
pYC308		pKP15-13 derived plasmid with *rprA* $T_{+2}T_{+3}$/GG mutation. pKP15-13 was re-amplified by JVO-13643/-9457 and self-ligated.	pBR322, Amp^R	This study
pKP-102-1	*sdaC*-10aa-*gfp*	GFP reporter plasmid carries the SdaC-10aa-gfp translational fusion.	pSC101*, Cm^R	(Papenfort et al., 2009)
pKP-125-1	*tpx*-70aa-*gfp*	GFP reporter plasmid carriers the Tpx-70aa-gfp translational fusion.	pSC101*, Cm^R	(Papenfort et al., 2009)
pYC348	*sdaC*-10aa-M'-*gfp*	pKP102-1 was re-amplified by overlapping PCR with JVO-14140/-14141.	pSC101*, Cm^R	This study
pYC349	*tpx*-70aa-M'-*gfp*	pKP125-1 was re-amplified by overlapping PCR with JVO-14243/-14244.	pSC101*, Cm^R	This study
pKF31-1	*cfa-gfp*	GFP reporter plasmid carries the *cfa*-10aa-gfp translational fusion.	pSC101*, Cm^R	(Fröhlich et al., 2013)
pYC311		pKF31-1 derivative. A T_{+2}/G mutation was introduced in pKF31-1 using overlapping PCR with JVO-13648/-13649 and Phusion polymerase.	pSC101*, Cm^R	This study
pXG-1	LacZ-GFP	Control vector containing wild type *gfp* under the control of pLtetO promoter.	pSC101*, Cm^R	(Urban and Vogel, 2007)
pXG-10		Backbone vector for constructing *gfp* translational fusions.	pSC101*, Cm^R	(Urban and Vogel, 2007)
pBAD/*Myc*-His A		pBAD backbone vector used for sRNA cloning.	pBR322, Amp^R	Invitrogen

Supplemental list of DNA oligonucleotides used in this study.

Name	Sequence	Comment
pZE-XbaI	TCGTTTTATTTGATGCCTCTAGA	Antisense oligo for *cfa* primer extension
JVO-0944	GTTTTTTTTAATACGACTCACTATAGGACGGTTAT AAATCAACACAT	Sense oligo for *rprA* T7 template, carries T7 promoter
JVO-3144	TCATGTTACCGGTAAAATACCACC	Antisense oligo to probe for STnc200
JVO-4045	TTTTTTGAATTCTAATACGACTCACTATAGGGTGC GGCCTGAAAACAG	Sense oligo for cloning of ArcZ, carries EcoRI site and T7 promoter
JVO-4049	GGGCAAAGACTACACACAGC	Antisense oligo to probe RprA
JVO-4055	GTTTTTATGCATGGTTGTTTATATTACGATAATTAT TAG	Sense oligo to amplify *cfa* from +1
JVO-4208	GAATACTGCGCCAACACCAG	Antisense oligo to probe ArcZ
JVO-4652	GTTTTTTTTAATACGACTCACTATAGGGACGTTAA CTATAAGTAAATAGGAA	Sense oligo for *tpx* T7 template, carries T7 promoter
JVO-4663	TCCAGTTTCGTGATATGTTTCA	Oligo to probe STnc840
JVO-4664	GGCTAACTTGCAGAGTACAGCTT	Oligo to probe STnc850
JVO-4666	GAAGACAGGGATGGTGTCTATG	Oligo to probe STnc870/CpxQ
JVO-5902	AAAAAGCCCATCGTAGGA	Antisense oligo for RprA T7 template
JVO-5954	TACAACGGGAAAAGATTAGCG	Oligo to probe STnc890
JVO-5957	GCGCAGACTATATCACCGAAG	Oligo to probe STnc920
JVO-5961	AAAATCCAACATAGCTAAATTAAAAATAAT	Oligo to probe STnc960
JVO-5963	AGAACTTGTTGTGCCGGATG	Oligo to probe STnc980
JVO-5968	GGACATGACACACGGATTTTAC	Oligo to probe STnc2030
JVO-5969	AAAGGATAATCAAAAGTTACTTTTTATTTTTAG	Oligo to probe STnc2040
JVO-5974	GGCGATACACTCAATGTAAGGG	Oligo to probe STnc2090
JVO-5978	CTGCAAAATCAGCACTGTGG	Oligo to probe STnc2130
JVO-5980	AATACTCAGGGAATACATGCCATC	Oligo to probe STnc2150
JVO-5981	AAAGTACCACTCAACGAAGTGTTATC	Oligo to probe STnc2160
JVO-5992	TGGCCAACCATGTCGAAA	Oligo to probe RybD, partially overlap with terminator
JVO-7106	TGACCCCGGTTTGACC	Antisense oligo for ArcZ primer extension
JVO-8755	AAAAATGACCCCGGTTTG	Antisense to ArcZ terminator. Use with JVO-4045 for T7 template
JVO-8798	CGCATACGCCAGTATCAATG	Oligo to probe *tpx* mRNA
JVO-9457	5'P~TCGTTGTTTCACTCAGGG	Antisense oligo to reamplify rprA 5' part in pKP15.
JVO-9590	GAAACGTATTAGCCGTGGCG	Oligo to probe *serC* 3'UTR
JVO-9591	ACTGCCAGGACGATATCCAC	Oligo to probe *celG* 3'UTR (STnc2010)
JVO-9594	GTGGGACTAAAGCTCTTGTTCAA	Oligo to probe *argR* 3'UTR
JVO-12432	5'P~GTGGCTGCGCCGTGCTTAT	Antisense oligo to reamplify *arcZ* 5' part in pKP4.
JVO-12433	GAGTTCCCTGGTGTTGGCGCAGTA	Sense oligo to reamplify *arcZ* 3' part in pKP4, to mutate U+1.
JVO-12434	GATGTCCCTGGTGTTGGCGCAGTA	Sense oligo to reamplify *arcZ* 3' part in pKP4, to mutate U+2.
JVO-12435	GAGGTCCCTGGTGTTGGCGCAGTA	Sense oligo to reamplify *arcZ* 3' part in pKP4, to mutate U+1U+2.
JVO-12436	GATGGCCCTGGTGTTGGCGCAGTA	Sense oligo to reamplify *arcZ* 3' part in pKP4, to mutate U+2U+3.
JVO-13641	AGTGCTGTGTGTAGTCTTTGCC	Sense oligo to mutate +2U in *rprA*, use with JVO-9457
JVO-13642	ATGGCTGTGTGTAGTCTTTGCC	Sense oligo to mutate +3U in *rprA*, use with JVO-9457
JVO-13643	AGGGCTGTGTGTAGTCTTTGCC	Sense oligo to mutate U+2U+3 in *rprA*, use with JVO-9457
JVO-13648	TCGGAGAACTGGCTCACGGAATTGT	Sense oligo to mutate U+2 in *cfa*
JVO-13649	CAGTTCTCCGACGTAGAACAGAGGAA	Antisense oligo to mutate U+2 in *cfa*.

JVO-13995	ACGACACTGCTTATTGCTTTG	Antisense oligo to probe leuZ-3’ETS
JVO-14140	CAGGAGCCATAGATGGAAACCACTCAGA	Sense oligo to introduce compensatory mutations in *sdaC*
JVO-14141	ATCTATGGCTCCTGGAGGATGCTAAAA	Antisense oligo to introduce compensatory mutations in *sdaC*
JVO-14243	CCAGGGCCACCCGGTCACCGTTGCCAAC	Sense oligo to introduce compensatory mutations in *tpx*
JVO-14244	CCGGGTGGCCCTGGAAATGTACAGTCTGTG	Antisense oligo to introduce compensatory mutations in *tpx*

5’P denotes 5’-phosphorylation.

Supplemental references

Bailey, T.L., Boden, M., Buske, F.A., Frith, M., Grant, C.E., Clementi, L., Ren, J., Li, W.W., and Noble, W.S. (2009). MEME SUITE: tools for motif discovery and searching. Nucleic Acids Res *37*, W202-208.

Bailey, T.L., Johnson, J., Grant, C.E., and Noble, W.S. (2015). The MEME Suite. Nucleic Acids Res *43*, W39-49.

Bandyra, K.J., Said, N., Pfeiffer, V., Gorna, M.W., Vogel, J., and Luisi, B.F. (2012). The seed region of a small RNA drives the controlled destruction of the target mRNA by the endoribonuclease RNase E. Mol Cell *47*, 943-953.

Barquist, L., Langridge, G.C., Turner, D.J., Phan, M.D., Turner, A.K., Bateman, A., Parkhill, J., Wain, J., and Gardner, P.P. (2013). A comparison of dense transposon insertion libraries in the Salmonella serovars Typhi and Typhimurium. Nucleic Acids Res *41*, 4549-4564.

Callaghan, A.J., Marcaida, M.J., Stead, J.A., McDowall, K.J., Scott, W.G., and Luisi, B.F. (2005). Structure of Escherichia coli RNase E catalytic domain and implications for RNA turnover. Nature *437*, 1187-1191.

Chao, Y., Papenfort, K., Reinhardt, R., Sharma, C.M., and Vogel, J. (2012). An atlas of Hfq-bound transcripts reveals 3' UTRs as a genomic reservoir of regulatory small RNAs. EMBO J *31*, 4005-4019.

Chao, Y., and Vogel, J. (2016). A 3' UTR-Derived Small RNA Provides the Regulatory Noncoding Arm of the Inner Membrane Stress Response. Mol Cell *61*, 352-363.

Chinni, S.V., Raabe, C.A., Zakaria, R., Randau, G., Hoe, C.H., Zemann, A., Brosius, J., Tang, T.H., and Rozhdestvensky, T.S. (2010). Experimental identification and characterization of 97 novel npcRNA candidates in Salmonella enterica serovar Typhi. Nucleic Acids Res *38*, 5893-5908.

Crooks, G.E., Hon, G., Chandonia, J.M., and Brenner, S.E. (2004). WebLogo: a sequence logo generator. Genome Res *14*, 1188-1190.

Figueroa-Bossi, N., Valentini, M., Malleret, L., Fiorini, F., and Bossi, L. (2009). Caught at its own game: regulatory small RNA inactivated by an inducible transcript mimicking its target. Genes Dev *23*, 2004-2015.

Forstner, K.U., Vogel, J., and Sharma, C.M. (2014). READemption-a tool for the computational analysis of deep-sequencing-based transcriptome data. Bioinformatics *30*, 3421-3423.

Fröhlich, K.S., Papenfort, K., Fekete, A., and Vogel, J. (2013). A small RNA activates CFA synthase by isoform-specific mRNA stabilization. EMBO J *32*, 2963-2979.

Gardner, P.P., Barquist, L., Bateman, A., Nawrocki, E.P., and Weinberg, Z. (2011). RNIE: genome-wide prediction of bacterial intrinsic terminators. Nucleic Acids Res *39*, 5845-5852.

Hess, B., Kutzner, C., Van Der Spoel, D., and Lindahl, E. (2008). GROMACS 4: algorithms for highly efficient, load-balanced, and scalable molecular simulation. Journal of chemical theory and computation *4*, 435-447.

Hoffmann, S., Otto, C., Kurtz, S., Sharma, C.M., Khaitovich, P., Vogel, J., Stadler, P.F., and Hackermuller, J. (2009). Fast mapping of short sequences with mismatches, insertions and deletions using index structures. PLoS Comput Biol *5*, e1000502.

Hoiseth, S.K., and Stocker, B.A. (1981). Aromatic-dependent Salmonella typhimurium are non-virulent and effective as live vaccines. Nature *291*, 238-239.

Humphrey, W., Dalke, A., and Schulten, K. (1996). VMD: visual molecular dynamics. J Mol Graph *14*, 33-38, 27-38.

Kaberdin, V.R. (2003). Probing the substrate specificity of Escherichia coli RNase E using a novel oligonucleotide-based assay. Nucleic Acids Res *31*, 4710-4716.

Kröger, C., Colgan, A., Srikumar, S., Handler, K., Sivasankaran, S.K., Hammarlof, D.L., Canals, R., Grissom, J.E., Conway, T., Hokamp, K., *et al.* (2013). An infection-relevant transcriptomic compendium for Salmonella enterica Serovar Typhimurium. Cell Host Microbe *14*, 683-695.

Kröger, C., Dillon, S.C., Cameron, A.D., Papenfort, K., Sivasankaran, S.K., Hokamp, K., Chao, Y., Sittka, A., Hebrard, M., Handler, K., *et al.* (2012). The transcriptional landscape and small RNAs of Salmonella enterica serovar Typhimurium. Proc Natl Acad Sci U S A *109*, E1277-1286.

Li, H., Handsaker, B., Wysoker, A., Fennell, T., Ruan, J., Homer, N., Marth, G., Abecasis, G., Durbin, R., and Genome Project Data Processing, S. (2009). The Sequence Alignment/Map format and SAMtools. Bioinformatics *25*, 2078-2079.

Lindahl, E., Hess, B., and Van Der Spoel, D. (2001). GROMACS 3.0: a package for molecular simulation and trajectory analysis. Molecular modeling annual *7*, 306-317.

Lorenz, R., Bernhart, S.H., Honer Zu Siederdissen, C., Tafer, H., Flamm, C., Stadler, P.F., and Hofacker, I.L. (2011). ViennaRNA Package 2.0. Algorithms Mol Biol *6*, 26.

Love, M.I., Huber, W., and Anders, S. (2014). Moderated estimation of fold change and dispersion for RNA-seq data with DESeq2. Genome Biol *15*, 550.

Mackie, G.A. (2013). RNase E: at the interface of bacterial RNA processing and decay. Nat Rev Microbiol *11*, 45-57.

Martick, M., and Scott, W.G. (2006). Tertiary contacts distant from the active site prime a ribozyme for catalysis. Cell *126*, 309-320.

Misra, T.K., and Apirion, D. (1979). RNase E, an RNA processing enzyme from Escherichia coli. J Biol Chem *254*, 11154-11159.

Nicol, J.W., Helt, G.A., Blanchard, S.G., Jr., Raja, A., and Loraine, A.E. (2009). The Integrated Genome Browser: free software for distribution and exploration of genome-scale datasets. Bioinformatics *25*, 2730-2731.

Noel, J.K., Whitford, P.C., Sanbonmatsu, K.Y., and Onuchic, J.N. (2010). SMOG@ctbp: simplified deployment of structure-based models in GROMACS. Nucleic Acids Res *38*, W657-661.

Oivanen, M., Kuusela, S., and Lonnberg, H. (1998). Kinetics and Mechanisms for the Cleavage and Isomerization of the Phosphodiester Bonds of RNA by Bronsted Acids and Bases. Chem Rev *98*, 961-990.

Papenfort, K., Espinosa, E., Casadesus, J., and Vogel, J. (2015). Small RNA-based feedforward loop with AND-gate logic regulates extrachromosomal DNA transfer in Salmonella. Proc Natl Acad Sci U S A *112*, E4772-4781.

Papenfort, K., Pfeiffer, V., Lucchini, S., Sonawane, A., Hinton, J.C., and Vogel, J. (2008). Systematic deletion of Salmonella small RNA genes identifies CyaR, a conserved CRP-dependent riboregulator of OmpX synthesis. Mol Microbiol *68*, 890-906.

Papenfort, K., Pfeiffer, V., Mika, F., Lucchini, S., Hinton, J.C., and Vogel, J. (2006). SigmaE-dependent small RNAs of Salmonella respond to membrane stress by accelerating global omp mRNA decay. Mol Microbiol *62*, 1674-1688.

Papenfort, K., Said, N., Welsink, T., Lucchini, S., Hinton, J.C., and Vogel, J. (2009). Specific and pleiotropic patterns of mRNA regulation by ArcZ, a conserved, Hfq-dependent small RNA. Mol Microbiol *74*, 139-158.

Perkins, T.T., Kingsley, R.A., Fookes, M.C., Gardner, P.P., James, K.D., Yu, L., Assefa, S.A., He, M., Croucher, N.J., Pickard, D.J., *et al.* (2009). A strand-specific RNA-Seq analysis of the transcriptome of the typhoid bacillus Salmonella typhi. PLoS Genet *5*, e1000569.

Quinlan, A.R. (2014). BEDTools: The Swiss-Army Tool for Genome Feature Analysis. Curr Protoc Bioinformatics *47*, 11 12 11-11 12 34.

Quinlan, A.R., and Hall, I.M. (2010). BEDTools: a flexible suite of utilities for comparing genomic features. Bioinformatics *26*, 841-842.

Sittka, A., Pfeiffer, V., Tedin, K., and Vogel, J. (2007). The RNA chaperone Hfq is essential for the virulence of Salmonella typhimurium. Mol Microbiol *63*, 193-217.

Torres, R.A., and Bruice, T.C. (1998). Molecular dynamics study displays near in-line attack conformations in the hammerhead ribozyme self-cleavage reaction. Proc Natl Acad Sci U S A *95*, 11077-11082.

Urban, J.H., and Vogel, J. (2007). Translational control and target recognition by Escherichia coli small RNAs in vivo. Nucleic Acids Res *35*, 1018-1037.

Van Der Spoel, D., Lindahl, E., Hess, B., Groenhof, G., Mark, A.E., and Berendsen, H.J. (2005). GROMACS: fast, flexible, and free. Journal of computational chemistry *26*, 1701-1718.

Westermann, A.J., Forstner, K.U., Amman, F., Barquist, L., Chao, Y., Schulte, L.N., Muller, L., Reinhardt, R., Stadler, P.F., and Vogel, J. (2016). Dual RNA-seq unveils noncoding RNA functions in host-pathogen interactions. Nature *529*, 496-501.

Whitford, P.C., Noel, J.K., Gosavi, S., Schug, A., Sanbonmatsu, K.Y., and Onuchic, J.N. (2009). An all-atom structure-based potential for proteins: bridging minimal models with all-atom empirical forcefields. Proteins *75*, 430-441.
