# Supplementary material for: In Vivo Cleavage Map Illuminates the Central Role of RNase E in Coding and Non-coding RNA Pathways
Source: Mol Cell. 2017 Jan 5;65(1):39–51. doi: 10.1016/j.molcel.2016.11.002 (PMC5222698; doi:10.1016/j.molcel.2016.11.002)
Supplement: Document S2. Article plus Supplemental Information [file mmc9.pdf]

Molecular Cell

Article

In Vivo Cleavage Map Illuminates the Central Role of RNase E in Coding and Non-coding RNA Pathways

Graphical Abstract

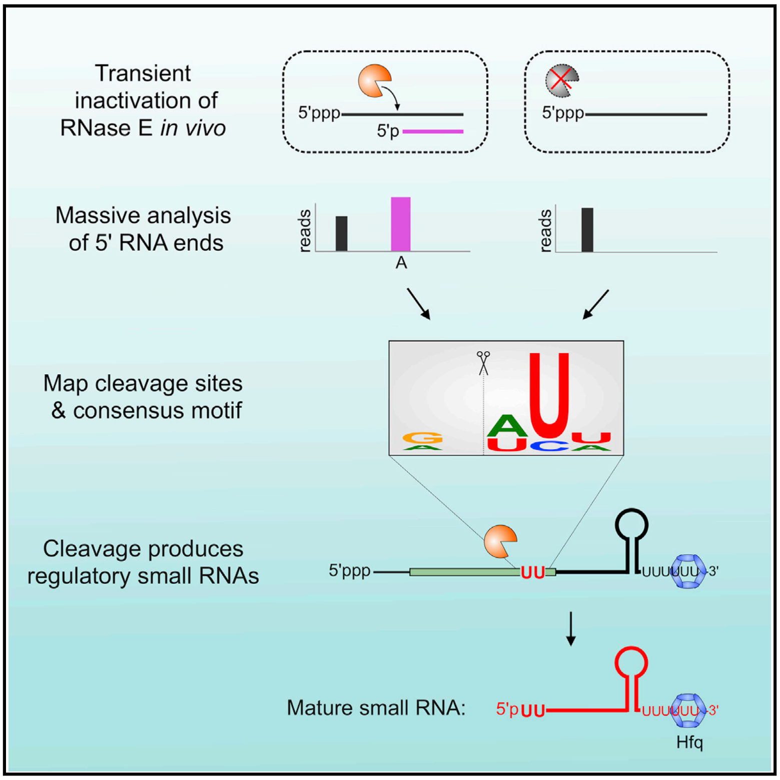

Highlights

- TIER-seq precisely maps ~22,000 endogenous RNase E cleavage sites in *Salmonella*
- Consensus motif of RNase E reveals a 2-nt uridine ruler-and-cut mechanism
- RNase E is a central component in both maturation and degradation of small RNAs
- There is a general small-RNA biogenesis pathway requiring RNase E and Hfq

Authors

Yanjie Chao, Lei Li, Dylan Girodat, ..., Hans-Joachim Wieden, Ben F. Luisi, Jörg Vogel

Correspondence

joerg.vogel@uni-wuerzburg.de

In Brief

Chao et al. discover that the essential bacterial RNase E cleaves numerous transcripts at preferred sites by sensing uridine as a 2-nt ruler. RNase E processing of various precursor RNAs produces many small regulatory RNAs, constituting a major small-RNA biogenesis pathway in bacteria.

Accession Numbers

GSE81869

Chao et al., 2017, Molecular Cell *65*, 39–51
January 5, 2017 © 2017 The Authors. Published by Elsevier Inc.
http://dx.doi.org/10.1016/j.molcel.2016.11.002

CrossMark

CellPress

CellPress

In Vivo Cleavage Map Illuminates the Central Role of RNase E in Coding and Non-coding RNA Pathways

Yanjie Chao,[1] Lei Li,[1,2] Dylan Girodat,[3] Konrad U. Förstner,[1,2] Nelly Said,[4] Colin Corcoran,[1] Michał Śmiga,[1] Kai Papenfort,[1,5] Richard Reinhardt,[6] Hans-Joachim Wieden,[3] Ben F. Luisi,[7] and Jörg Vogel[1,8,9,*]

[1]Institute of Molecular Infection Biology
[2]Core Unit Systems Medicine
University of Würzburg, 97080 Würzburg, Germany
[3]Alberta RNA Research and Training Institute, Department of Chemistry and Biochemistry, University of Lethbridge, Lethbridge, Alberta T1K 3M4, Canada
[4]Department of Biology I, Microbiology, Ludwig-Maximilians-Universität Munich, 82152 Martinsried, Germany
[5]Laboratory of Structural Biochemistry, Freie Universität Berlin, 14195 Berlin, Germany
[6]Max Planck Genome Centre Cologne, Max Planck Institute for Plant Breeding Research, 50829 Cologne, Germany
[7]Department of Biochemistry, University of Cambridge, Cambridge CB2 1GA, UK
[8]Helmholtz Institute for RNA-based Infection Research (HIRI), 97080 Würzburg, Germany
[9]Lead Contact
*Correspondence: joerg.vogel@uni-wuerzburg.de

http://dx.doi.org/10.1016/j.molcel.2016.11.002

SUMMARY

Understanding RNA processing and turnover requires knowledge of cleavages by major endoribonucleases within a living cell. We have employed TIER-seq (transiently inactivating an endoribonuclease followed by RNA-seq) to profile cleavage products of the essential endoribonuclease RNase E in *Salmonella enterica*. A dominating cleavage signature is the location of a uridine two nucleotides downstream in a single-stranded segment, which we rationalize structurally as a key recognition determinant that may favor RNase E catalysis. Our results suggest a prominent biogenesis pathway for bacterial regulatory small RNAs whereby RNase E acts together with the RNA chaperone Hfq to liberate stable 3′ fragments from various precursor RNAs. Recapitulating this process in vitro, Hfq guides RNase E cleavage of a representative small-RNA precursor for interaction with a mRNA target. In vivo, the processing is required for target regulation. Our findings reveal a general maturation mechanism for a major class of post-transcriptional regulators.

INTRODUCTION

Small, non-coding RNAs (sRNAs) that associate with the RNA chaperone Hfq constitute the largest class of post-transcriptional regulators in Gram-negative bacteria (De Lay et al., 2013; Storz et al., 2011; Vogel and Luisi, 2011; Wagner and Romby, 2015). Initially defined as a class in non-pathogenic *Escherichia coli* (Zhang et al., 2003), Hfq-dependent sRNAs have been globally mapped in numerous important human pathogens (Barquist and Vogel, 2015; Holmqvist et al., 2016; Koo et al., 2011; Melamed et al., 2016; Tree et al., 2014). These sRNAs generally act as multi-target repressors and activators through seed pairing interactions with the 5′ untranslated region (UTR) of mRNAs (Desnoyers et al., 2013; Feng et al., 2015; Papenfort and Vanderpool, 2015). A full understanding of these sRNA-mediated networks requires knowledge of how their RNA constituents are synthesized and turned over.

Many of the bacterial sRNAs characterized to date are transcribed from non-coding intergenic regions and operate as full-length, primary transcripts capped with a 5′ triphosphate (5′ PPP). However, some primary sRNAs such as ArcZ and RprA are converted into shorter stable species that retain the seed region for target mRNA recognition (Mandin and Gottesman, 2010; Papenfort et al., 2009, 2015). It is currently unclear whether such processing generates the active sRNAs, as is the case with eukaryotic microRNAs (Kim, 2005). Moreover, several recent studies reported sRNAs that are produced from the 3′ region of mRNA genes (Miyakoshi et al., 2015b), only a subset of which are the result of gene-internal promoters (Chao et al., 2012; Guo et al., 2014), while many others appear to originate from mRNA processing. These 3′-derived sRNAs are likely to be functional, since they abundantly associate with Hfq (Chao et al., 2012), whose cellular concentration is limited (Wagner, 2013). Their physiological importance is further supported by established roles of the 3′-mRNA-derived sRNAs CpxQ and SroC in the envelope stress response or amino acid pathways, respectively (Chao and Vogel, 2016; Miyakoshi et al., 2015a). Furthermore, 3′ fragments of *E. coli* tRNA precursors function as molecular sponges of conserved sRNAs (Lalaouna et al., 2015). Collectively, these findings suggest that sRNA processing is a prevalent event; however, both its functional relevance and the major responsible nuclease(s) remain to be established.

Of several candidate nucleases involved in sRNA processing and turnover, the conserved and essential endoribonuclease E (RNase E) is the likely central player (Mackie, 2013; Massé et al., 2003; Saramago et al., 2014). It can be inferred, from transcript accumulation upon its inactivation, that RNase E drives the

Molecular Cell 65, 39–51, January 5, 2017 © 2017 The Authors. Published by Elsevier Inc.
This is an open access article under the CC BY license (http://creativecommons.org/licenses/by/4.0/).
CrossMark

decay of most mRNAs in *E. coli* (Bernstein et al., 2004; Clarke et al., 2014), and in *Salmonella* it processes the mRNA 3′ end-derived CpxQ and SroC sRNAs (Chao and Vogel, 2016; Miyakoshi et al., 2015a). RNase E also degrades several sRNAs in the absence of Hfq or upon base pairing with target mRNAs (Bandyra et al., 2012; Massé et al., 2003; Moll et al., 2003). Conversely, some sRNAs activate gene expression by blocking RNase E cleavage sites in target mRNAs (Fröhlich et al., 2013; Papenfort et al., 2013). In addition, RNase E is known to engage in rRNA and tRNA precursor processing (Apirion and Lassar, 1978; Bessarab et al., 1998; Kime et al., 2014; Li and Deutscher, 2002; Ow and Kushner, 2002).

Despite the importance of RNase E in post-transcriptional control, its activity toward most non-coding RNAs is not known. Previous studies have characterized major RNase E cleavage sites in a few abundant model transcripts (e.g., Apirion and Lassar, 1978; Delvillani et al., 2011; Ehretsmann et al., 1992; Mackie, 1991; Ow and Kushner, 2002; Patel and Dunn, 1992; Régnier and Hajnsdorf, 1991; Roy and Apirion, 1983) and concluded that the enzyme preferentially cleaves AU-rich regions in single-stranded RNA (Arraiano et al., 2010; Huang et al., 1998; McDowall et al., 1994, 1995). Here, to achieve a systems-level understanding of RNase E activity, we have analyzed in depth the in vivo RNase E cleavage events in *Salmonella typhimurium*, a close relative of *E. coli* and a pathogenic model organism to study post-transcriptional regulation (Westermann et al., 2016). Our genome-wide capture of tens of thousands of endogenous cleavage sites reveals a minimal consensus sequence and a 2-nt uridine ruler-and-cut structural mechanism for this major endoribonuclease. Intriguingly, RNase E employs this mechanism to cleave many coding and non-coding transcripts at the 3′ end and releases stable, Hfq-bound RNA fragments, indicating that sRNA biogenesis through endonucleolytic processing is widespread. Searches for these predicted critical uridines in sRNAs enabled us to show that maturation by RNase E is essential for target regulation by the ArcZ sRNA. Moreover, our data reveal a high frequency of RNase-E-mediated cleavages in Hfq-dependent sRNAs, supporting the functional link between RNase E and Hfq for the first time on a global level.

RESULTS

A Transcriptome-wide Map of RNase E Cleavage Sites In Vivo

To globally map RNase E cleavage events in vivo, we profiled 5′ ends of cellular transcripts by comparative RNA-seq before and 30 min after programmed inactivation of the enzyme using a temperature-sensitive *rne*^{TS} mutant (*rne*-3071) (Apirion and Lassar, 1978; Figueroa-Bossi et al., 2009). We refer to this approach, which builds upon work by Clarke and colleagues (Clarke et al., 2014) as transient inactivation of endoribonuclease followed by RNA-seq (TIER-Seq; see Figure 1A). At the permissive temperature (28°C), *Salmonella* wild-type (WT) *rne* and mutant *rne*^{TS} strains both exhibit full RNase E activity, whereas upon shift to 44°C, only WT RNase E retains its activity to process RNA. To achieve a comprehensive RNase-E-specific "degradome" analysis at single-nucleotide resolution (Figure 1A), we analyzed biological duplicates of all four of the above strains and conditions in the early stationary growth phase (OD_{600} of 2) by RNA-seq, obtaining ~130 million reads (Figure S1A). In agreement with previous work showing that RNase E cleaves AU-rich sequences (McDowall et al., 1994, 1995), the inactivation of RNase E leads to a ~5% reduction of cDNA reads with 5′-A/T bases (Figure S1B).

To pinpoint cleavage sites, we aligned all reads to the *Salmonella* genome, mapping a total of ~500,000 unique 5′ ends (Figures 1B and 1C). WT and *rne*^{TS} samples from growth at 28°C gave nearly identical 5′ end profiles (R^2 = 0.98; Figures 1B and S1C), confirming that the mutant RNase E is fully functional at the permissive temperature, whereas at the non-permissive temperature (44°C), many positions were selectively depleted in the *rne*^{TS} cDNA libraries (Figure 1C). Since *Salmonella* has no 5′→3′ exoribonuclease (Hui et al., 2014), we interpret these depleted positions as RNase E cleavage sites (Figure 1A). This classification is supported by the capture of many previously known *E. coli* RNase E cleavage sites (Figure 1D)—for example, in the *rpsO*, *cspE*, *uncC/atpC*, and *glmUS* mRNAs (Delvillani et al., 2011; Joanny et al., 2007; Patel and Dunn, 1992; Régnier and Hajnsdorf, 1991), in the 9S precursor of 5S rRNA (Roy and Apirion, 1983), and near the 3′ end of tRNAs (Ow and Kushner, 2002). Applying a threshold of >3-fold as significant depletion ($p < 0.05$, FDR < 0.05) in the *rne*^{TS} samples at 44°C, we assigned 22,033 RNase-E-mediated cleavages in the *Salmonella* transcriptome, expanding by several orders of magnitude the database of in vivo target sites for this ribonuclease. The full list of cleavage sites is available in Table S1.

A Systems-Level View on RNase E Activity in RNA Metabolism

Systematic analysis of the 22,033 RNase E cleavage sites revealed their distribution in coding and non-coding transcripts from the *Salmonella* chromosome and virulence plasmids (Figure 2A): ~80% occurred in mRNAs, primarily in the coding sequence (CDS), indicating that a major activity of RNase E is to degrade mRNAs in addition to processing housekeeping RNAs. Altogether, we detected a total of 2,557 mRNAs cleaved by RNase E, with a different number of cleavage sites per transcript (Figure 2B); these represent 78% of 3,286 *Salmonella* mRNAs that are well expressed (RPKM > 10, Table S2) in the early stationary phase. Notably, the assay captured many essential genes and virulence genes required for intracellular growth (Table S3), which provide insights into the processing of transcripts from indispensable genes and the roles of RNase E in *Salmonella* pathogenesis (Viegas et al., 2013), respectively. Longer transcripts generally tend to contain a higher number of cleavage sites (Figures S1D and S1E). After normalizing the number of cleavage sites to gene length, RNase E cleavage frequency in these genes (RPKM > 10) ranges from 0 to ≥30 sites per kilobase, with a median value at ~5.7 cleavages per kilobase, or one site every ~175 nt of mRNA (Figure 2C). This non-saturating cleavage pattern might suggest that most sites in mRNAs are inaccessible, perhaps due to structural constraints or protein binding.

The position of an RNase E site within a transcript may provide information about the function of the cleavage. For example, RNase E auto-regulates its synthesis by cutting in the 5′ UTR

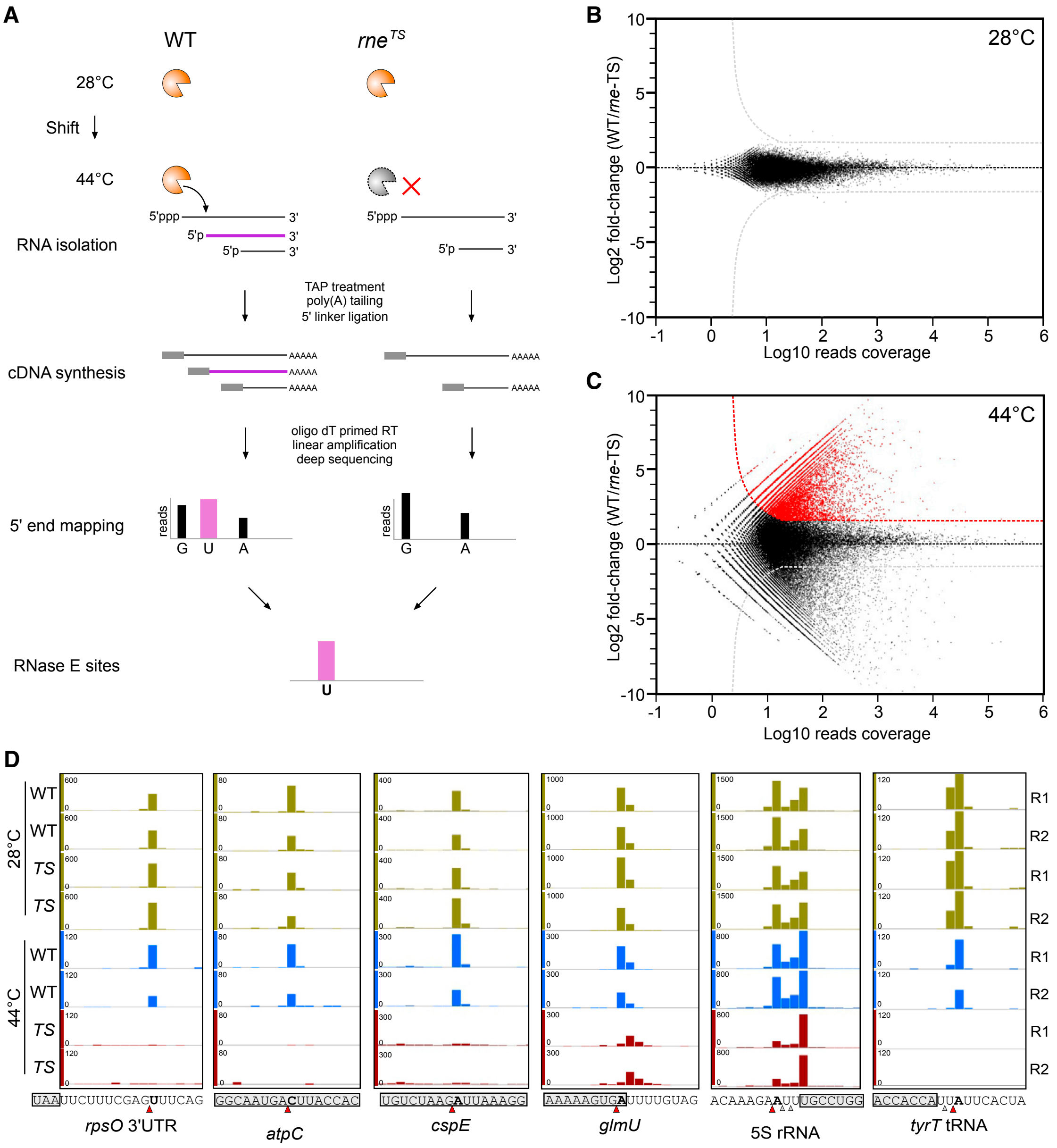

Figure 1. Global Mapping of Endogenous RNase E Cleavage Sites in *Salmonella* using TIER-Seq

(A) Schema of the TIER-seq approach. Endogenous cleavage sites were identified by analyzing the 5′ ends of RNase E cleavage products (purple) in the WT and *rne*TS strains at the non-permissive temperature (44°C). Total RNA from WT and *rne*TS was converted to cDNAs and sequenced; the 5′ ends depleted in the *rne*TS libraries at 44°C indicate the RNase E cleavage sites (e.g., purple U).

(B and C) Global analysis of 5′ end profile at the permissive temperature 28°C (B) and non-permissive temperature 44°C (C). The plots show the read counts for every 5′ base in WT samples and the relative fold change compared to *rne*TS samples. Candidate RNase E cleavage sites that show >3-fold depletion in *rne*TS samples ($p < 0.05$, FDR < 0.05) are colored in red.

(D) TIER-seq captures known RNase E cleavage sites with single-nucleotide resolution. *TS* indicates the *rne*TS samples. R1 and R2 are two biological replicates. The major RNase E sites are marked by red arrowheads and bold lettering; secondary cleavage sites are indicated by open arrowheads. The ORF or mature RNAs are shadowed by gray boxes. See also Figures S1 and S2.

of its own mRNA (Jain and Belasco, 1995); our analysis readily captured this critical site (Figure S2A). As another example, we detect the RNase E site in the 5′ UTR of *cfa* mRNA (Figure S2A) that becomes protected by the *trans*-acting RydC sRNA, with the consequence that the transcript is stabilized (Dimastrogiovanni et al., 2014; Fröhlich et al., 2013). Thus, our candidate list of ~1,300 RNase E cleavage sites identified in the 5′ UTRs of 548 genes (Table S4) provides a resource to predict sites for post-transcriptional control by sRNAs and/or RNA-binding proteins.

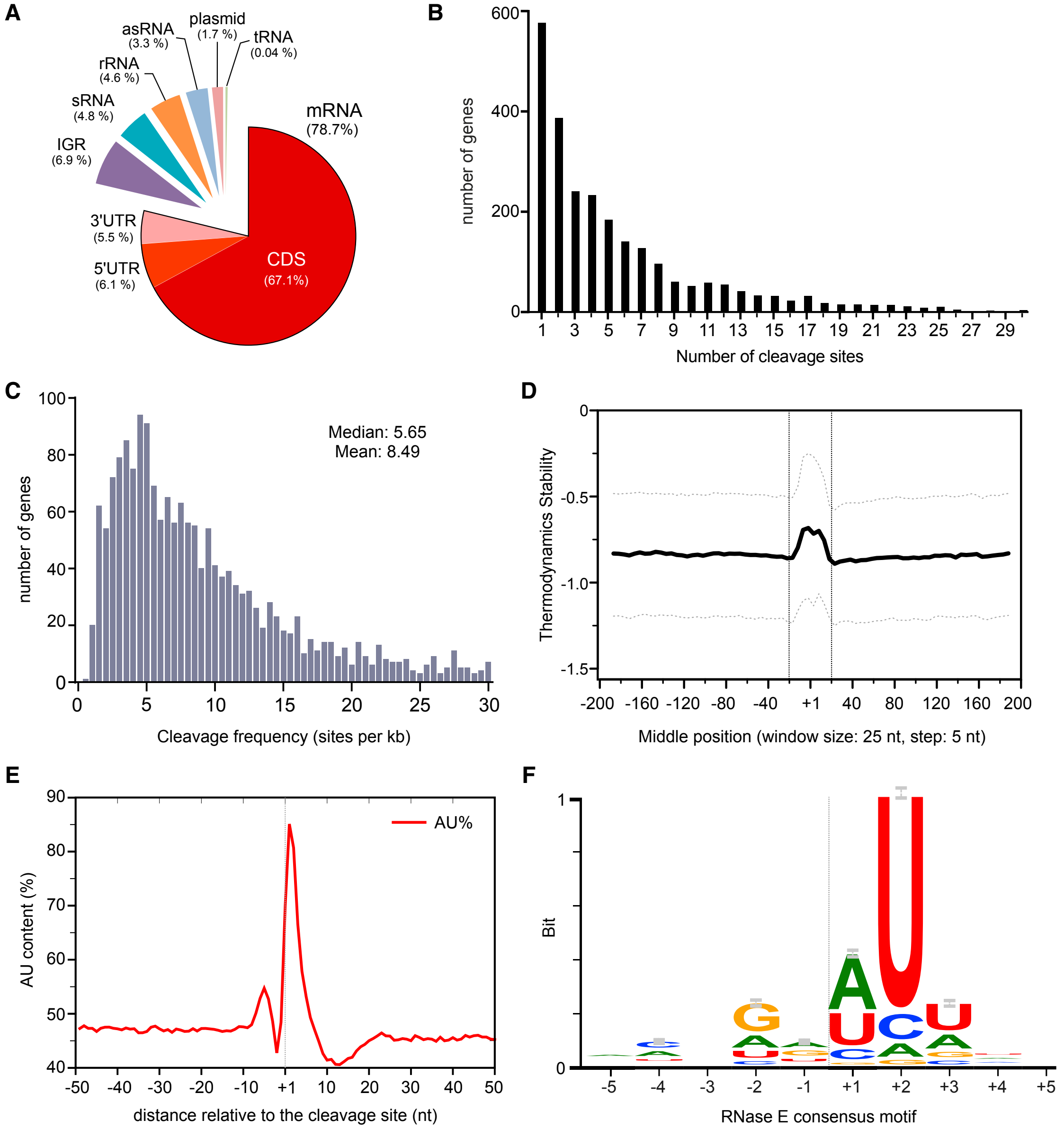

Figure 2. Systems-wide Analysis of Cleavage Sites Reveals a Consensus for RNase E

(A) Classification of all RNase E cleavage sites. The proportion (%) of all sites mapped within a category is shown. See also Table S1.

(B) The number of cleavage sites mapped per mRNA gene.

(C) The distribution of RNase E cleavage frequencies in mRNAs (RPKM > 10). See also Figure S1.

(D) Sequences at the RNase E sites are less structured. Minimal folding energy (MFE) was calculated for each 25 nt using a sliding window and was compared to randomly shuffled sequences. Median *Z* score is shown as a bold line; dotted lines indicate the upper and lower quartile.

(E) Distribution of AU content at the RNase E cleavage sites. Dashed line indicates the cleavage site (+1 nt).

(F) The RNase E consensus motif based on alignment of all mapped cleavage sites. Error bars indicate 95% confidence intervals. See also Figures S1 and S2.

A Specific Sequence Motif Recognized by RNase E

Even seemingly non-specific nucleases often exhibit a certain degree of sequence or structural preference. To understand the substrate determinants of RNase E activity, we analyzed the primary sequences and putative secondary structures around all of the 22,033 cleavage sites. At the cleavage site we observed an overall increase in the calculated folding energy (ΔG), indicating little secondary structure (Figure 2D), and a spike of AU-rich sequences (Figure 2E), both of which agree with previously studied individual RNase E sites (McDowall et al., 1994, 1995). Importantly, sequence alignment of all 22,033 sites predicts a minimal RNase E consensus sequence (Figure 2F) with a marked preference for uridine at the +2 position in the 5 nt “RN↓WUU” core motif (with R as G/A, W as A/U, and N as any nucleotide). This RNase E motif, based entirely on global in vivo data, fully recapitulates

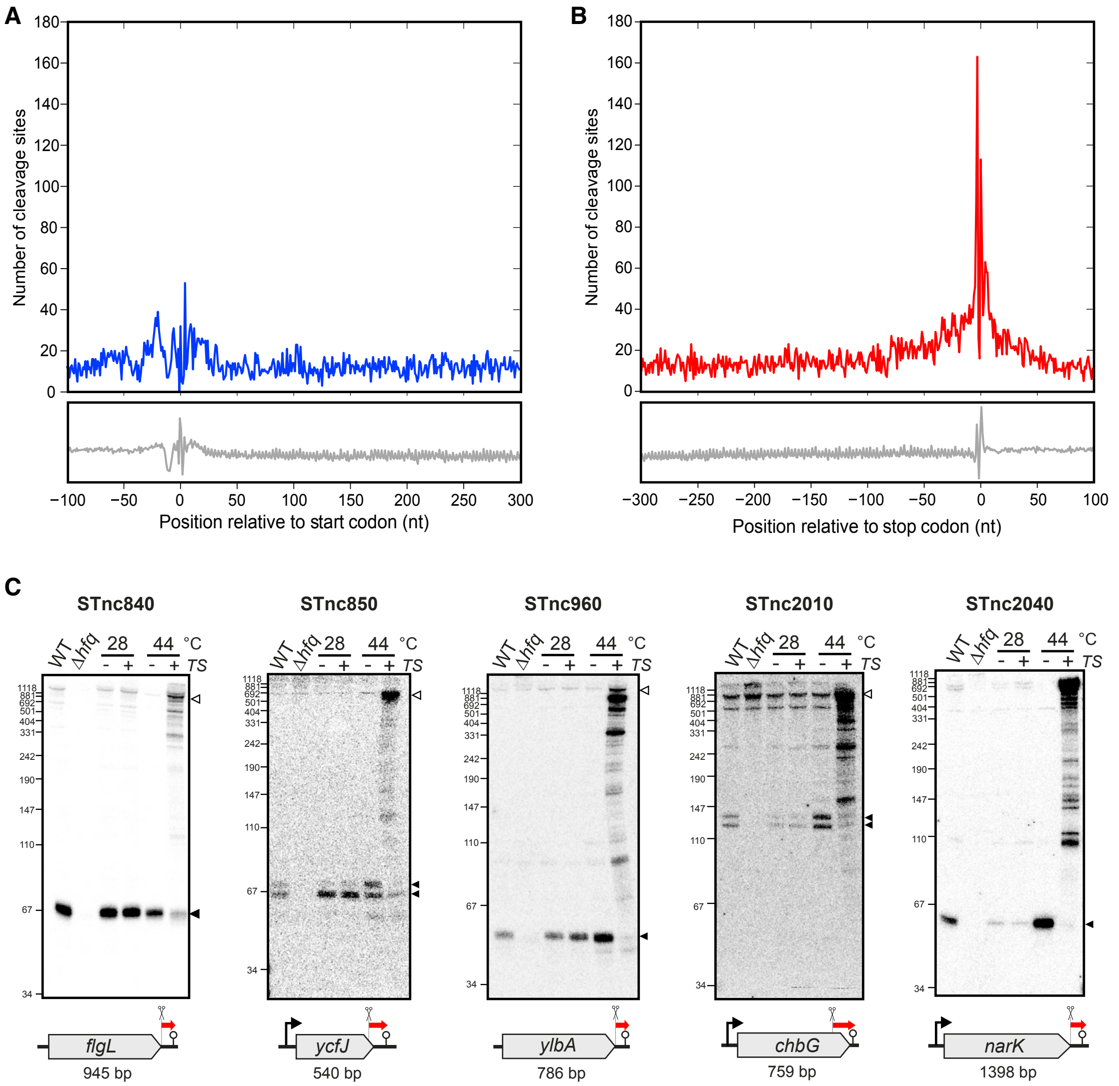

Figure 3. RNase E Cleaves mRNAs to Produce 3′ UTR-Derived sRNAs

(A and B) Distribution of RNase E cleavage sites in mRNAs relative to their start codon (A) or stop codon (B). The gray lines in the lower panel indicate the distribution of consensus motif based on genomic sequence.

(C) RNase E and Hfq are required for the biogenesis of 3′ UTR-derived sRNAs. WT and Δ*hfq* strains were grown at 37°C to an OD_{600} of 2. The location of sRNAs (red arrows) and host genes are shown in the lower panel. Promoters (where available) and terminators are shown. The 5S rRNA served as loading control (Figure S2C). See also Figures S2, S3, and S4.

preferences previously documented with model substrates in vitro (Ehretsmann et al., 1992; Kaberdin, 2003; Mackie, 1991) and with cell-derived RNA (Del Campo et al., 2015), while it clearly differs from recognition motifs of other major bacterial endoribonucleases such as tRNA-processing RNase P (McClain et al., 1987) or RNase III, which cleaves double-stranded RNA (Gan et al., 2005).

RNase E Cleavages Underlie sRNA Biogenesis from 3′ UTRs

In analyzing cleavage-site distributions relative to mRNA start or stop codons (Figures 3A and 3B), we observed that, on average, 5′ UTRs and the coding regions showed similar cleavage frequencies. Translation initiation regions were slightly counter-selected, perhaps because the prominent Shine-Dalgarno sequence (GGAGGA) is devoid of RNase E cleavage motifs. In contrast, RNase E sites were enriched around mRNA stop codons (Figure 3B); the high AU-rich content and/or translation termination may favor this enrichment. Since bacterial 3′ UTRs are generally short (Belasco, 2010), many of these stop codon sites may represent the most downstream sites, leaving 3′ fragments for degradation by 3′ → 5′ exoribonucleases. Interestingly, approximately one-third of these mRNAs carry protective ρ-independent terminators (Arraiano et al., 2010) that can, in principle, interact with the sRNA chaperone Hfq (Otaka et al., 2011; Sauer and Weichenrieder, 2011). These data point to the

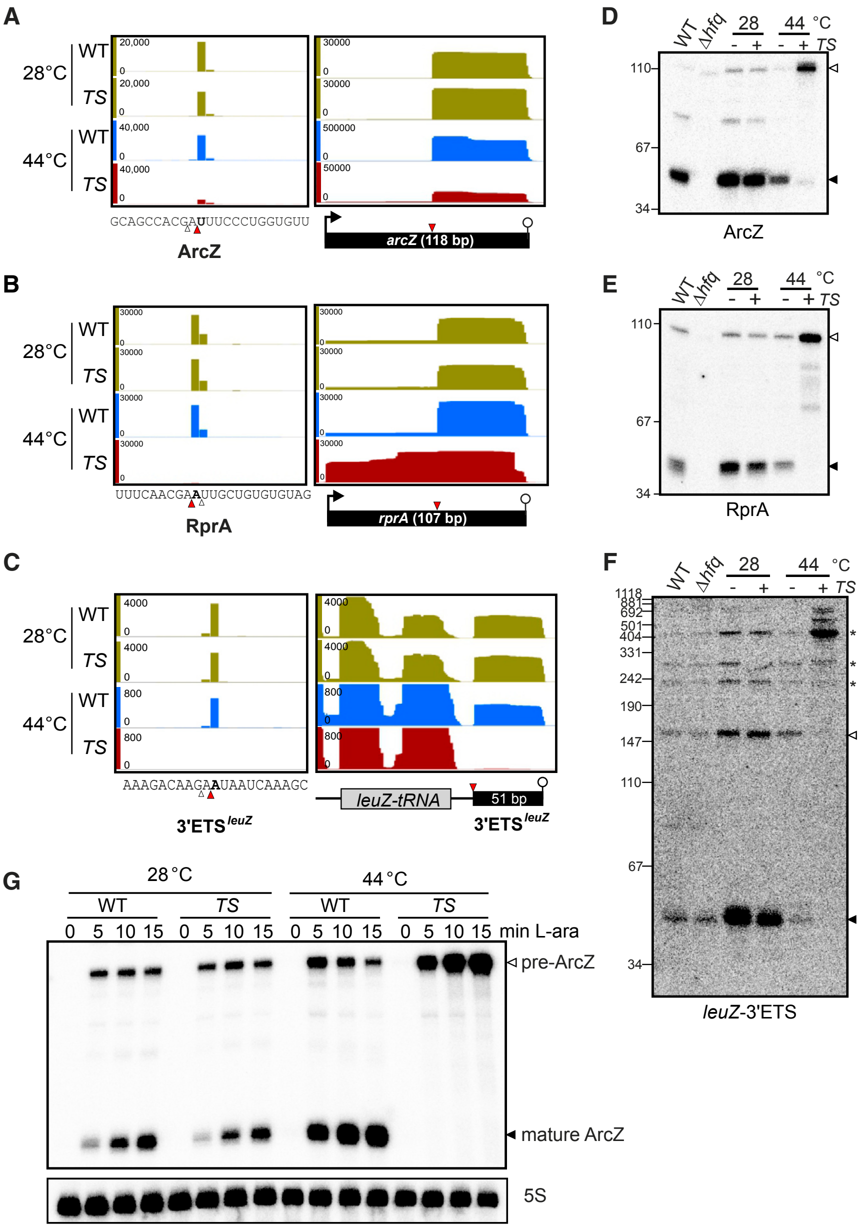

Figure 4. RNase E Cleaves Non-coding RNAs to Release 3′ Mature sRNAs

(A–C) RNase E cleavage sites are identified in the ArcZ sRNA (A), RprA (B), and 3′ETS^{leuZ} (C). The major sites are marked by red arrowheads and bold lettering, whereas the minor sites are indicated by open arrowheads. See also Figures S5 and S6.

(D–F) RNase E is required for the processing of ArcZ (D), RprA (E), and 3′ETS^{leuZ} (F). Open arrowheads indicate precursor fragments and filled arrowheads indicate processed mature species. * indicates longer precursors of polycistronic LeuZ-tRNA fragments; 5S loading controls, see Figure S2C.

(G) The maturation of ArcZ is dependent on RNase E activity. Expression of the full-length ArcZ precursor (pre-ArcZ) was induced by L-arabinose.

possibility that stable 3′ UTR fragments accumulate with functional consequence in the guise of regulatory sRNAs (Table S5; Chao et al., 2012; Miyakoshi et al., 2015b). Indeed, we have detected the mRNA 3′ UTR processing sites that produce the CpxQ and SroC sRNAs (Figure S2B). Northern blot probing of several selected candidates revealed distinct RNA species from mRNA 3′ ends, the generation of which required both active RNase E and the presence of Hfq (Figures 3C and S2C). Most of these 3′-derived sRNAs co-accumulate with their parental mRNA transcripts and possess potential seed regions (Figure S3), suggesting that they are bona fide regulatory sRNAs with conserved targets and functions. In addition, the cleavage sites in these sRNAs resemble the "RNWUU" sequence (Figure S2D), supporting the recognition of this consensus by RNase E (Figure 2F).

Cleavage by RNase E Produces sRNAs from Non-coding RNA Precursors

The majority of well-characterized, Hfq-dependent sRNAs in *E. coli* and *Salmonella* are primary transcripts of 50–250 nt in length. Although previous work on a few model sRNAs has implicated RNase E in their decay (Göpel et al., 2013; Madhugiri et al., 2010; Miyakoshi et al., 2015a; Viegas et al., 2007), it is unknown whether this sRNA class is generally processed by RNase E. Here, we have mapped ~600 RNase E cleavage sites in 107 experimentally validated sRNAs (Table S6), corroborating previously proposed sites in model sRNAs such as DsrA and MicA (Figures S4A and S4B; Moll et al., 2003). RNase E seems to preferentially target sRNAs that are bound by Hfq, as there are more cleavage sites in Hfq-dependent sRNAs compared to those that are Hfq independent (Chao et al., 2012; Figure S4C). Additionally, many cleavage sites in these sRNAs mapped to the vicinity of the seed region (Figure S4D), as exemplified by their clustering in the well-characterized seed of SgrS and RybB (Figures S4E and S4F). These data suggest that RNase E may inactivate sRNAs by removing the seed region; this is in agreement with previous results for MicC (Bandyra et al., 2012) and RyhB (Massé et al., 2003; Moll et al., 2003). Both MicC and RyhB are turned over by RNase E through seed cleavage if the target is absent, and this could provide a surveillance mechanism for accurate seed matching (Bandyra et al., 2012).

Another group of sRNAs is spared from immediate degradation following RNase E cleavage; instead, these RNAs appear to be processed by the enzyme. The highly conserved ArcZ and RprA sRNAs, which each regulate a number of targets, including *rpoS* (Majdalani et al., 2001; Mandin and Gottesman,

2010; Papenfort et al., 2009, 2015), provide cogent examples in which RNase E converts a precursor into a stable, shorter sRNA form (Figure 4). For both ArcZ and RprA, the detected cleavage sites precisely match the RNase E consensus motif (Figures 4A–4C) and are fully consistent with the size of the previously documented ~50 nt 3′ species of these sRNAs (Argaman et al., 2001; Mandin and Gottesman, 2010; Papenfort et al., 2009, 2015). These 3′ species accumulated to significantly higher levels than the primary sRNAs in an Hfq-dependent manner (Figures 4D and 4E). When RNase E was inactivated for 30 min, these shorter ArcZ and RprA species became undetectable on northern blots (Figures 4D and 4E; lane rne^{TS}, 44°C), suggesting a primary role for the enzyme in the processing event. To independently evaluate the function of RNase E in processing these sRNAs in vivo, each RNA was expressed from a plasmid-borne promoter subsequent to heat inactivation of the enzyme. While the full-length sRNAs accumulated under this condition, they were not converted into the short 3′ species (Figures 4G and S5B). These findings establish RNase E as a primary nuclease for generating functional short ArcZ and RprA, both of which regulate numerous *trans*-encoded target mRNAs (Papenfort et al., 2009, 2015).

Hfq-dependent regulatory RNA can also originate from other types of precursors, such as polycistronic tRNA transcripts. One such precursor is the sRNA sponge *leuZ*-3′ETS (Lalaouna et al., 2015), which was suggested to be processed by RNase E during *leuZ*-tRNA maturation (Li and Deutscher, 2002). Our TIER-seq data confirm that the 5′ end of *leuZ*-3′ETS is generated by RNase E and pinpoints the cleavage site to an adenine 15 nt downstream of the mature *leuZ*-tRNA (Figure 4C). Using the rne^{TS} strain, we observe RNase E to be essential for the production of this sRNA sponge (Figure 4F). Together, these results argue for a major role of RNase E in maturing non-coding regulatory RNAs from different types of cellular transcripts.

Determinants of RNase E in sRNA Processing

To understand how RNase E matures Hfq-associated sRNAs, we chose ArcZ for further characterization (Figure 5A). Using the purified catalytic domain (NTD) of RNase E in combination with Hfq, we could readily reconstitute in vitro the release of 3′ ArcZ (56 nt) from its 118-nt-long precursor (pre-ArcZ) prepared with T7 RNA polymerase (Figure 5B). Within 3 min, the reaction produced the mature ArcZ fragment, which accumulated over time; the cleavage occurred precisely at the expected sites identified by TIER-seq in vivo (Figure 5D). However, in the absence of Hfq, RNase E rapidly hydrolyzed pre-ArcZ into fragments without producing 3′ ArcZ (Figure 5B). This suggests that Hfq plays a role in directing the correct processing of ArcZ by RNase E.

The maturation site in ArcZ in *Salmonella* and *E. coli* matches well with our TIER-seq-derived RNase E consensus (GA↓$U_{+1}U_{+2}U_{+3}$; Figure 5A versus Figure 2F), featuring uridines ($U_{+2}U_{+3}$) at the second and third position downstream of the cleavage site that are highly conserved in numerous enterobacterial species (Papenfort et al., 2009). Strikingly, changing U_{+2} to a disfavored G in the RNase E motif strongly diminished ArcZ processing by RNase E in vitro (Figures 5C and 5D), and processing was fully inhibited by further mutating U_{+3}. By contrast, the same change at U_{+1} alone had little if any effect (Figures 5C and 5D). To explore if these findings have bearing on the maturation process in vivo, we expressed mutant ArcZ variants from inducible pBAD plasmids and analyzed the status of the ArcZ sRNA. Consistent with the in vitro results, the $U_{+2} \rightarrow G_{+2}$ mutation strongly reduced the levels of 3′ ArcZ in *Salmonella* (Figure 5E), with further reductions upon additional mutation of the upstream ($U_{+1}U_{+2} \rightarrow G_{+1}G_{+2}$) and downstream ($U_{+2}U_{+3} \rightarrow G_{+2}G_{+3}$) uridines. Of note, the processing of ArcZ seems to be required for the regulation of its target *tpx* mRNA (see below).

The crucial roles of U_{+2} and Hfq in RNase E cleavage were also evident for the RprA sRNA (Figure S5). Full-length RprA precursor (pre-RprA) was processed by RNase E in vitro at its internal seed sequence (GA↓$A_{+1}U_{+2}U_{+3}$), producing mature RprA only in the presence of Hfq. Mutating U_{+2} alone significantly reduced the maturation of RprA by RNase E, which was fully abolished by changing both $U_{+2}U_{+3}$ to non-preferred guanines. The essentiality of U_{+2} in RprA processing could also be demonstrated in vivo (Figure S5C), as well as in directing the cleavage of the *cfa* mRNA (Figure S2E). Together, these mutational studies further validate our TIER-seq-based prediction of U_{+2} as a key nucleotide for specific RNase E cleavage of cellular transcripts.

RNase-E-Dependent sRNA Maturation Is Essential for Target Regulation

To consider RNase E as an sRNA maturation factor with functional consequences requires that its processing activity is essential for sRNA function. Demonstrating such a property requires first the development of a system in which processing of an sRNA precursor can be impeded without changing or losing the seed region. The ArcZ sRNA offers such a system: exploiting our finding that mutation of the crucial U_{+2} in the RNase E motif of ArcZ abolished cleavage enabled us to produce pre-ArcZ with diminished amounts of 3′ ArcZ in vivo (Figure 5). We examined the ability of the pre-ArcZ to repress the synthesis of Tpx (Figure 5E), whose mRNA is targeted by the conserved seed region of ArcZ (Papenfort et al., 2009; Figures 6A and S6). While a 10 min expression of WT ArcZ downregulated the *tpx* mRNA by 7-fold, the $U_{+2} \rightarrow G_{+2}$ mutant (variant GAUGU) achieved only 3-fold repression despite the higher levels of precursor (Figure 5E). Additional mutation of an adjacent uridine (variants GAGGU or GAUGG) fully inhibited 3′ ArcZ production and abrogated *tpx* regulation despite higher levels of the precursor, strongly suggesting that only the mature 3′ ArcZ is the functional regulator.

According to previous work (Papenfort et al., 2009), the $U_{+2}U_{+3}$ residues in the RNase E site of ArcZ may not engage in base paring with *tpx* (Figure 6A). If they do at all, they might extend the duplex by two additional A:U pairs; this could be disrupted by the non-functional, locked GAUGG variant of pre-ArcZ. To rule out that the failure of the GAUGG variant (ArcZ-GG) to repress *tpx* was simply due to insufficient base pairing, we introduced a compensatory AU→CC mutation in the *tpx*-GFP fusion (Tpx-CC), but again no regulation by the ArcZ-GG variant was observed (Figure 6B). Likewise, the processing-deficient ArcZ-GG variant also failed to regulate the *sdaC* mRNA target either

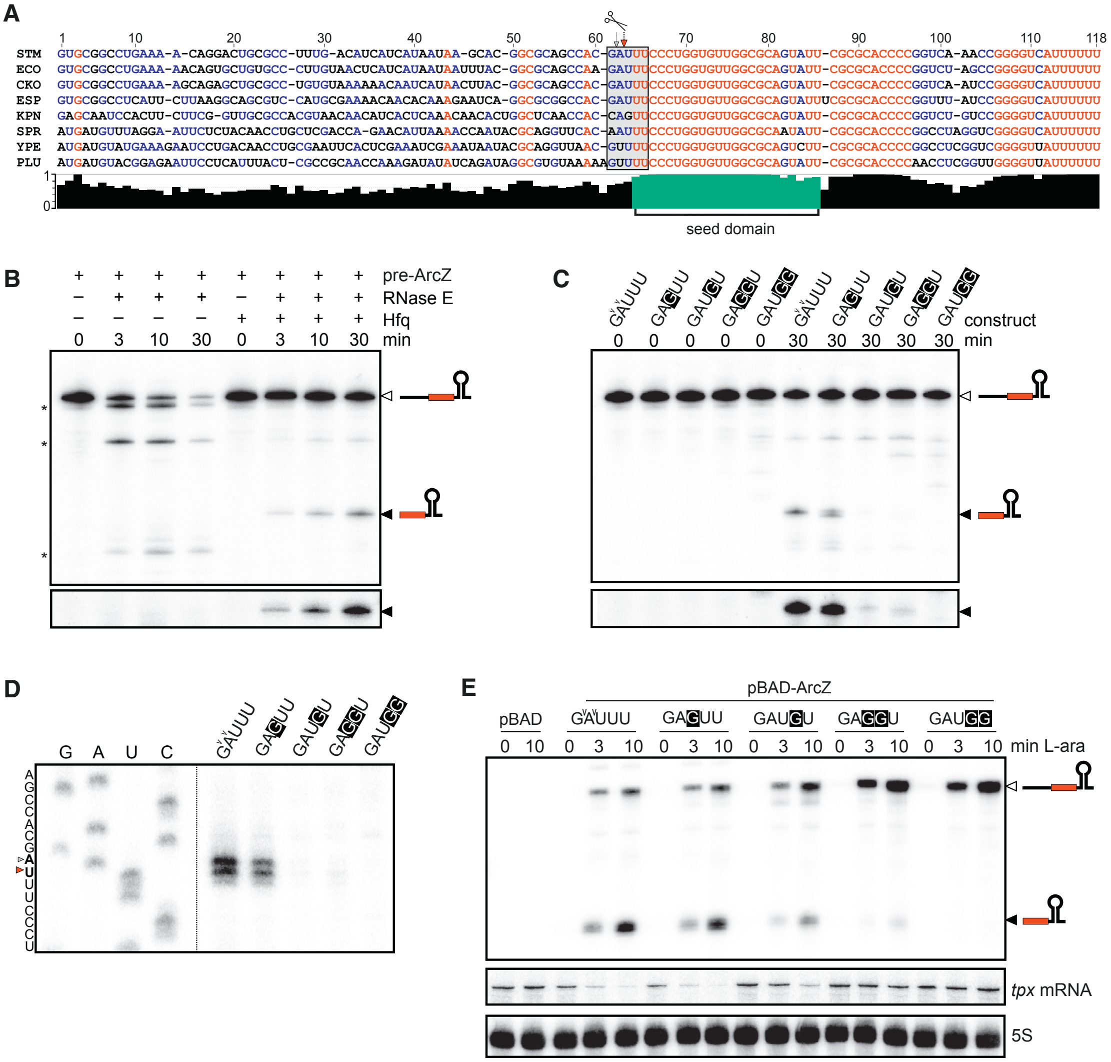

Figure 5. RNase E Mediates the Maturation of ArcZ sRNA In Vitro and In Vivo

(A) Alignment of ArcZ sequence. Conservation scores are plotted below the sequences, and the conserved seed is colored in green.

(B) Reconstitution of ArcZ maturation in vitro. Full-length pre-ArcZ RNA was incubated with RNase E in the presence or absence of Hfq. RNA was analyzed by northern blotting with an oligo antisense to the mature ArcZ. The lower set shows mature ArcZ signals with longer exposure.

(C) Mutation of RNase E cleavage site. Variants of ArcZ precursors were incubated with Hfq, and then subjected to RNase E cleavage. The lower set shows mature ArcZ signals with longer exposure.

(D) Primer extension to map the RNase E cleavage sites in ArcZ in vitro.

(E) Validation of RNase E motif in ArcZ in vivo. See also Figures S5 and S6.

in its WT form or with a duplex-extending CC mutation (Figure S6B). Thus, RNase E cleavage is essential for the production of functional ArcZ.

A likely explanation for ArcZ maturation to be essential for regulation is that the ArcZ seed may only become available for target pairing upon RNase E cleavage. To test this, we examined sRNA duplex formation with *tpx* mRNA in vitro. Electrophoretic mobility shift assays with radiolabeled sRNA showed that the mature 3′ ArcZ binds to the target region of *tpx* mRNA (a 216 nt fragment containing 5′ UTR and early CDS) with very high affinity ($K_D \approx 15$ nM; Figure 6C); by contrast, an ~500-fold excess of pre-ArcZ over target was insufficient for full duplex formation (Figure 6D), similar to the low affinity observed for pre-ArcZ binding to the *rpoS* mRNA (Soper et al., 2010). In addition, Hfq promotes formation of the sRNA target duplex in the case of mature ArcZ, but less so for pre-ArcZ (Figures S6C and S6D). These results were further confirmed by reciprocal experiments with labeled *tpx* mRNA. Again, mature ArcZ readily bound to the target and formed a stable ArcZ-*tpx*-Hfq ternary complex (Figure 6E), whereas excess of the pre-ArcZ RNA only competed with the *tpx*-Hfq complex formation and released free *tpx* mRNA. These combined in vivo and in vitro results show that pre-ArcZ undergoes an RNase-E-dependent maturation to activate ArcZ for

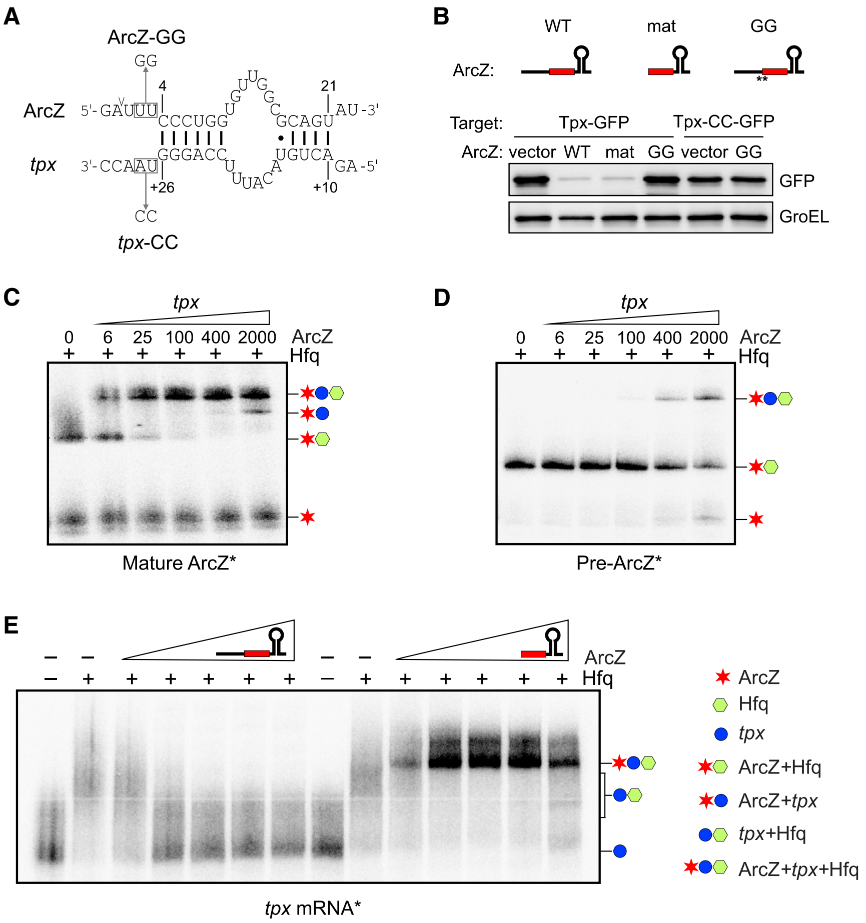

Figure 6. Maturation of ArcZ sRNA Is Essential for Target Regulation

(A) Established base pair interactions between ArcZ and *tpx* mRNA (Papenfort et al., 2009). The major cleavage site in ArcZ is indicated.

(B) Western blot detection of GFP levels. GFP was fused with *tpx* 5′ UTR; the introduced mutations are shown in (A). "WT" refers to WT full-length ArcZ, "mat" refers to mature ArcZ, and "GG" refers to the GAUGG variant of ArcZ. GroEL served as loading control.

(C) Direct interaction of *tpx* with mature ArcZ by EMSA. Radiolabeled mature ArcZ was incubated with increasing concentration of *tpx* mRNA in the presence of Hfq (40 nM). The gel was resized; see Figure S6.

(D) Direct interaction of *tpx* with pre-ArcZ by EMSA.

(E) Mature ArcZ was co-shifted with *tpx* mRNA. Radiolabeled *tpx* mRNA was incubated with increasing concentration of pre-ArcZ or mature ArcZ (0, 6, 25, 100, 400, and 2,000 nM) in the presence of 40 nM Hfq. See also Figure S6.

repression of *tpx* and perhaps other targets. This demonstrates for the first time that RNase E cleavage is required to activate an Hfq-dependent sRNA.

DISCUSSION

Bacterial transcripts are generally short lived (Bernstein et al., 2002; Chen et al., 2015) and subject to rapid turnover by cellular ribonucleases (Hui et al., 2014; Mackie, 2013). Gene expression and regulation typically take place at the level of primary transcripts bearing the native 5′ PPP end. This is fundamentally different from higher eukaryotes, where nearly all types of regulatory transcripts undergo processing and maturation as a prerequisite for function. Our identification of numerous conserved regulatory sRNAs that result from RNase E cleavage (Figures 3 and S3) illustrates the complexity of the bacterial "RNA degradome." These increasing numbers of processing-derived RNA species (Chao and Vogel, 2016; Davis and Waldor, 2007; Deltcheva et al., 2011; Guo et al., 2014; Miyakoshi et al., 2015a) contrast with the general perception that cleaved bacterial transcripts are usually labile species of little biological relevance.

TIER-seq offers a generic approach both for global analysis of processed transcripts and cleavage sites in living cells with single-nucleotide resolution and for mechanistic understanding of ribonuclease activities at a systems level. We have here employed a temperature-sensitive strain to transiently inactivate the endogenous RNase E, which minimizes the potentially confounding effects of "non-native" conditions used in previous degradome studies where the nucleases were genetically deleted (Linder et al., 2014), ectopically overexpressed (Schifano et al., 2014), or supplemented in vitro (Clarke et al., 2014). To circumvent the need for a thermosensitive mutant and temperature-induced transcriptomic changes (Table S7), future TIER-seq studies may benefit from using alternative means of transient nuclease inactivation such as small molecules (Kime et al., 2015), small inhibitory proteins (Kim et al., 2008; Lee et al., 2003), target-specific proteases (Cameron and Collins, 2014), or conditionally spliced inteins (Zeidler et al., 2004).

RNase-E-Dependent sRNA Biogenesis and Maturation in Bacteria

We identify RNase E as a key factor both for the biogenesis of many 3′ UTR-derived sRNAs and for the maturation of active sRNAs from their non-coding precursors. This establishes RNase E cleavage as a second major pathway for the biogenesis of Hfq-dependent sRNAs (Figure 7A). As compared to the canonical pathway of de novo transcription, this cleavage-based biogenesis may confer several advantages. RNase E can generate sRNAs from diverse origins, including essentially all existing transcripts (Figure 7A), greatly expanding the sRNA repertoire in the cell. This pathway could reduce regulatory overhead during evolution of new genes (Mattick, 2004), using the existing regulatory elements of the parental transcripts to control the expression of 3′-derived sRNAs. Activating an internal seed sequence by sRNA precursor cleavage, as shown here for ArcZ, offers an additional layer of control in post-transcriptional regulation—for example, via an adaptor protein such as RapZ,

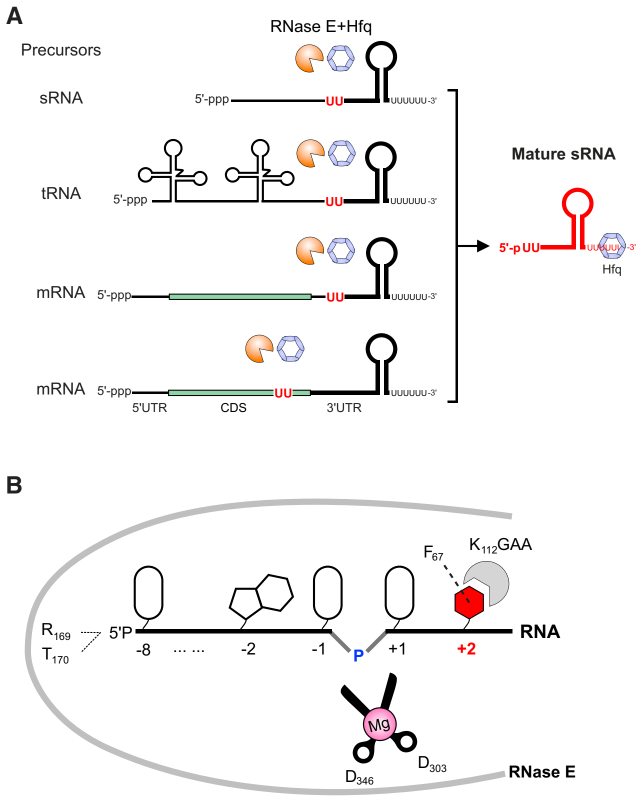

Figure 7. Mechanism of RNase E Cleavage and an Alternative sRNA Biogenesis Pathway

(A) RNase E cleavage constitutes a major sRNA biogenesis pathway in bacteria.

(B) Proposed model for the +2 uridine ruler-and-cut mechanism of specific RNase E cleavage. The scissile phosphate is attacked hydrolytically by a water molecule (not shown) that is coordinated by the magnesium ion bound by the carboxylates of D_{346} and D_{303}. Stacking interactions (between F_{67} and K_{112}) and hydrogen bonding (with the K_{112}GAA loop) with the base at position +2 favor uridine at this position. The interactions are predicted to help orient the phosphate backbone into a geometry that would facilitate cleavage at the scissile phosphate. See also Figure S7.

which facilitates specific RNase E cleavage in certain sRNAs (Göpel et al., 2013). Lastly, RNase-E-cleavage-derived sRNAs carry a 5′ P end which promotes mRNA target degradation (Bandyra et al., 2012; Chao and Vogel, 2016; Pfeiffer et al., 2009) and, as a consequence, different regulation kinetics than translational control alone (Levine and Hwa, 2008).

The key role of RNase E in sRNA biogenesis mirrors the central role of this enzyme in mRNA target regulation by many Hfq-dependent sRNAs (Massé et al., 2003; Saramago et al., 2014; Vogel and Luisi, 2011). Importantly, target degradation was proposed to involve tripartite RNase-E-based ribonucleoprotein complexes with sRNA and Hfq (Ikeda et al., 2011; Morita et al., 2005; Worrall et al., 2008). Our results indicate that this complex may form in order to mediate the alternative biogenesis of sRNAs prior to their target decay. For example, an ArcZ-Hfq-RNase E complex must form in the course of ArcZ maturation from the Hfq-bound, pre-ArcZ sRNA. In this respect, the Hfq-RNase E complex in bacteria could have a dual function: it processes precursor transcripts to stable, mature sRNA and guides the mature sRNA for target regulation.

A U_{+2} Ruler-and-Cut Mechanism Mediates Specific RNase E Cleavage

The hallmark of the RNase E consensus motif inferred from our in vivo map (Figure 2) is a predominant uridine at 2 nt downstream of the cleavage sites (U_{+2}), and we provide in vivo and in vitro evidence that the U_{+2} is crucial for RNase E cleavage. Analysis of the available crystal structure of an RNase E-RNA complex shows that the enzyme interacts with RNA at +2 nt via a stable stacking interaction of the nucleobase with Phe_{67} and Lys_{112} (Callaghan et al., 2005; Mackie, 2013). However, this structure contains a non-cognate substrate with G_{+2}, representing a stable RNA-binding conformation trapped at the pre-cleavage state. Why is a uridine at this position preferred for cleavage? A molecular dynamics simulation analysis in which G_{+2} is substituted for U in silico suggests that the RNase E-RNA complex undergoes a conformational change favored by the presence of U_{+2}; this allows us to propose a new model (Figure 7B) whereby RNase E mediates specific cleavage using a U_{+2} ruler-and-cut mechanism. Simulations of the pre-cleavage state show that U_{+2} was tightly bound in a crevice of the protein formed by the backbone of the $Lys_{112}Gly_{113}Ala_{114}Ala_{115}$ loop and the Lys_{112} side chain, resulting in a binding pocket that favors uracil (uracil pocket, Figures S7A–S7C and supplemental discussion). Importantly, the presence of the cognate U_{+2} promotes a distortion of the phosphodiester backbone angles at the cleavage site 2 nt upstream. The new conformation of the scissile phosphate may closely resemble, with slight deviation, the pseudo-trigonal, bipyramidal geometry that facilitates in-line nucleophilic attack of scissile phosphate (Figures 7B and S7D–S7F). While we have shown here that mutating U_{+2} in RNA abolishes cleavage, mutation of the critical Lys_{112} also abrogates RNase E cleavage of cognate substrates (Callaghan et al., 2005). The high conservation of residues forming the uracil pocket (e.g., Phe_{67} and Lys_{112}) indicates that this may be a conserved mechanism for the RNase E protein family.

The uridine ruler-and-cut mechanism is also employed by other endoribonucleases, including the unrelated human nuclease RNase L. RNase L recognizes uridine in single-stranded RNAs and cleaves 2 nt downstream (Han et al., 2014), whereas RNase E cuts 2 nt upstream due to different dimeric structure arrangements. Interestingly, a fraction of RNase E sites contain C_{+2} (Figure 2F), indicating that RNase E displays a certain degree of flexibility by accepting a cytosine in the absence of other specificity signals. Indeed, in vitro experiments with poly(A) RNA demonstrate that C_{+2} can serve as a cleavage signal (Kaberdin, 2003), which further suggests that RNase E may distinguish the smaller pyrimidine from purine bases by steric hindrance (Figures 7B, S7A, and S7B). Nevertheless, U_{+2} is the preferred signal (Figure 2F), likely because its C_4 oxygen possesses hydrogen bonding potential with RNase E (Figure S7B). In addition, some flexibility of RNase E is reflected near the cleavage sites, as RNase E frequently cuts 1 nt upstream or downstream of the determined cleavage site. To

compensate for this, short stretches of uridines (1–4 U) are often found at the +2 positions, which may serve to reinforce RNase E recognition and cleavage (e.g., ArcZ; Figure 5).

Our identification of crucial U_{+2} residues for RNase-E-specific cleavage enables straightforward mutations of individual cleavage sites of interest instead of global inactivation of the enzyme. This will aid the molecular investigation of 3′ UTR-derived sRNAs and of RNase-E-mediated, post-transcriptional regulations, not only in the Hfq regulon but also for the recently discovered class of ProQ-associated sRNAs (Smirnov et al., 2016)—many of which might be RNase E targets, too. This information may also help design novel CRISPR-Cas or antisense-RNA-based synthetic tools to activate gene expression by specifically blocking a cleavage site, as well as helping to engineer stable mRNAs for better gene expression.

EXPERIMENTAL PROCEDURES

Full methods are described in the Supplemental Experimental Procedures; so are details of bacterial strains, plasmids, and oligonucleotides.

Transient Inactivation of RNase E

The *Salmonella rne*^{TS} strains refer to *rne*-3071 and its isogenic WT control previously established in (Figueroa-Bossi et al., 2009). Bacteria were grown in Lennox LB medium at 28°C to an OD_{600} of 2, then shifted to 44°C for 30 min to inactivate RNase E.

RNA-Seq and Data Analysis

cDNA libraries were constructed following a standard protocol (Chao et al., 2012; Westermann et al., 2016). Briefly, RNA was polyadenylated at 3′ end and ligated to an adaptor at 5′ end after treatment with tobacco acid pyrophosphatase. First-strand cDNA was synthesized using oligo(dT)-adaptor and M-MLV reverse transcriptase. The linear amplified cDNAs were multi-plexed and sequenced using Illumina HiSeq. Reads were mapped to *Salmonella* genome using READemption; 5′ end coverage was visualized in IGB. The RNase E sites, which are depleted 5′ ends in the *rne*^{TS} samples relative to WT at 44°C, were identified using DESeq2.

ACCESSION NUMBERS

The sequencing data have been deposited in the GEO database under GEO: GSE81869.

SUPPLEMENTAL INFORMATION

Supplemental Information includes Supplemental Experimental Procedures, seven figures, and seven tables and can be found with this article online at http://dx.doi.org/10.1016/j.molcel.2016.11.002.

AUTHOR CONTRIBUTIONS

Y.C. and J.V. conceived the research; Y.C., N.S., C.C., M.S., and K.P. conducted experiments; Y.C., L.L., K.U.F., and B.F.L. analyzed data; D.G. and H.-J.W. performed MD simulations; R.R. performed RNA-seq; Y.C., B.F.L., and J.V. wrote the manuscript.

ACKNOWLEDGMENTS

We thank L. Bossi for *Salmonella* strain *rne*-3071, K. Bandyra for purified RNase E protein, S. Gorski for editing, K. McDowall for sharing data, and T. Yano and B. Plaschke for technical assistance. This study was funded by DFG (Vo875/14-1) and BioSysNet grants. B.F.L. is supported by the Wellcome Trust. K.P. was supported by the Human Frontiers Science Program (CDA00024/2016-C).

Received: June 9, 2016
Revised: September 26, 2016
Accepted: October 31, 2016
Published: January 5, 2017

REFERENCES

Apirion, D., and Lassar, A.B. (1978). A conditional lethal mutant of Escherichia coli which affects the processing of ribosomal RNA. J. Biol. Chem. *253*, 1738–1742.

Argaman, L., Hershberg, R., Vogel, J., Bejerano, G., Wagner, E.G., Margalit, H., and Altuvia, S. (2001). Novel small RNA-encoding genes in the intergenic regions of Escherichia coli. Curr. Biol. *11*, 941–950.

Arraiano, C.M., Andrade, J.M., Domingues, S., Guinote, I.B., Malecki, M., Matos, R.G., Moreira, R.N., Pobre, V., Reis, F.P., Saramago, M., et al. (2010). The critical role of RNA processing and degradation in the control of gene expression. FEMS Microbiol. Rev. *34*, 883–923.

Bandyra, K.J., Said, N., Pfeiffer, V., Górna, M.W., Vogel, J., and Luisi, B.F. (2012). The seed region of a small RNA drives the controlled destruction of the target mRNA by the endoribonuclease RNase E. Mol. Cell *47*, 943–953.

Barquist, L., and Vogel, J. (2015). Accelerating discovery and functional analysis of small RNAs with new technologies. Annu. Rev. Genet. *49*, 367–394.

Belasco, J.G. (2010). All things must pass: contrasts and commonalities in eukaryotic and bacterial mRNA decay. Nat. Rev. Mol. Cell Biol. *11*, 467–478.

Bernstein, J.A., Khodursky, A.B., Lin, P.H., Lin-Chao, S., and Cohen, S.N. (2002). Global analysis of mRNA decay and abundance in Escherichia coli at single-gene resolution using two-color fluorescent DNA microarrays. Proc. Natl. Acad. Sci. USA *99*, 9697–9702.

Bernstein, J.A., Lin, P.H., Cohen, S.N., and Lin-Chao, S. (2004). Global analysis of Escherichia coli RNA degradosome function using DNA microarrays. Proc. Natl. Acad. Sci. USA *101*, 2758–2763.

Bessarab, D.A., Kaberdin, V.R., Wei, C.L., Liou, G.G., and Lin-Chao, S. (1998). RNA components of Escherichia coli degradosome: evidence for rRNA decay. Proc. Natl. Acad. Sci. USA *95*, 3157–3161.

Callaghan, A.J., Marcaida, M.J., Stead, J.A., McDowall, K.J., Scott, W.G., and Luisi, B.F. (2005). Structure of Escherichia coli RNase E catalytic domain and implications for RNA turnover. Nature *437*, 1187–1191.

Cameron, D.E., and Collins, J.J. (2014). Tunable protein degradation in bacteria. Nat. Biotechnol. *32*, 1276–1281.

Chao, Y., and Vogel, J. (2016). A 3′ UTR-derived small RNA provides the regulatory noncoding arm of the inner membrane stress response. Mol. Cell *61*, 352–363.

Chao, Y., Papenfort, K., Reinhardt, R., Sharma, C.M., and Vogel, J. (2012). An atlas of Hfq-bound transcripts reveals 3′ UTRs as a genomic reservoir of regulatory small RNAs. EMBO J. *31*, 4005–4019.

Chen, H., Shiroguchi, K., Ge, H., and Xie, X.S. (2015). Genome-wide study of mRNA degradation and transcript elongation in Escherichia coli. Mol. Syst. Biol. *11*, 781.

Clarke, J.E., Kime, L., Romero A, D., and McDowall, K.J. (2014). Direct entry by RNase E is a major pathway for the degradation and processing of RNA in Escherichia coli. Nucleic Acids Res. *42*, 11733–11751.

Davis, B.M., and Waldor, M.K. (2007). RNase E-dependent processing stabilizes MicX, a Vibrio cholerae sRNA. Mol. Microbiol. *65*, 373–385.

De Lay, N., Schu, D.J., and Gottesman, S. (2013). Bacterial small RNA-based negative regulation: Hfq and its accomplices. J. Biol. Chem. *288*, 7996–8003.

Del Campo, C., Bartholomäus, A., Fedyunin, I., and Ignatova, Z. (2015). Secondary structure across the bacterial transcriptome reveals versatile roles in mRNA regulation and function. PLoS Genet. *11*, e1005613.

Deltcheva, E., Chylinski, K., Sharma, C.M., Gonzales, K., Chao, Y., Pirzada, Z.A., Eckert, M.R., Vogel, J., and Charpentier, E. (2011). CRISPR RNA

maturation by trans-encoded small RNA and host factor RNase III. Nature *471*, 602–607.

Delvillani, F., Papiani, G., Dehò, G., and Briani, F. (2011). S1 ribosomal protein and the interplay between translation and mRNA decay. Nucleic Acids Res. *39*, 7702–7715.

Desnoyers, G., Bouchard, M.P., and Massé, E. (2013). New insights into small RNA-dependent translational regulation in prokaryotes. Trends Genet. *29*, 92–98.

Dimastrogiovanni, D., Fröhlich, K.S., Bandyra, K.J., Bruce, H.A., Hohensee, S., Vogel, J., and Luisi, B.F. (2014). Recognition of the small regulatory RNA RydC by the bacterial Hfq protein. eLife *3*.

Ehretsmann, C.P., Carpousis, A.J., and Krisch, H.M. (1992). Specificity of Escherichia coli endoribonuclease RNase E: in vivo and in vitro analysis of mutants in a bacteriophage T4 mRNA processing site. Genes Dev. *6*, 149–159.

Feng, L., Rutherford, S.T., Papenfort, K., Bagert, J.D., van Kessel, J.C., Tirrell, D.A., Wingreen, N.S., and Bassler, B.L. (2015). A qrr noncoding RNA deploys four different regulatory mechanisms to optimize quorum-sensing dynamics. Cell *160*, 228–240.

Figueroa-Bossi, N., Valentini, M., Malleret, L., Fiorini, F., and Bossi, L. (2009). Caught at its own game: regulatory small RNA inactivated by an inducible transcript mimicking its target. Genes Dev. *23*, 2004–2015.

Fröhlich, K.S., Papenfort, K., Fekete, A., and Vogel, J. (2013). A small RNA activates CFA synthase by isoform-specific mRNA stabilization. EMBO J. *32*, 2963–2979.

Gan, J., Tropea, J.E., Austin, B.P., Court, D.L., Waugh, D.S., and Ji, X. (2005). Intermediate states of ribonuclease III in complex with double-stranded RNA. Structure *13*, 1435–1442.

Göpel, Y., Papenfort, K., Reichenbach, B., Vogel, J., and Görke, B. (2013). Targeted decay of a regulatory small RNA by an adaptor protein for RNase E and counteraction by an anti-adaptor RNA. Genes Dev. *27*, 552–564.

Guo, M.S., Updegrove, T.B., Gogol, E.B., Shabalina, S.A., Gross, C.A., and Storz, G. (2014). MicL, a new σE-dependent sRNA, combats envelope stress by repressing synthesis of Lpp, the major outer membrane lipoprotein. Genes Dev. *28*, 1620–1634.

Han, Y., Donovan, J., Rath, S., Whitney, G., Chitrakar, A., and Korennykh, A. (2014). Structure of human RNase L reveals the basis for regulated RNA decay in the IFN response. Science *343*, 1244–1248.

Holmqvist, E., Wright, P.R., Li, L., Bischler, T., Barquist, L., Reinhardt, R., Backofen, R., and Vogel, J. (2016). Global RNA recognition patterns of post-transcriptional regulators Hfq and CsrA revealed by UV crosslinking in vivo. EMBO J. *35*, 991–1011.

Huang, H., Liao, J., and Cohen, S.N. (1998). Poly(A)- and poly(U)-specific RNA 3′ tail shortening by E. coli ribonuclease E. Nature *391*, 99–102.

Hui, M.P., Foley, P.L., and Belasco, J.G. (2014). Messenger RNA degradation in bacterial cells. Annu. Rev. Genet. *48*, 537–559.

Ikeda, Y., Yagi, M., Morita, T., and Aiba, H. (2011). Hfq binding at RhlB-recognition region of RNase E is crucial for the rapid degradation of target mRNAs mediated by sRNAs in Escherichia coli. Mol. Microbiol. *79*, 419–432.

Jain, C., and Belasco, J.G. (1995). RNase E autoregulates its synthesis by controlling the degradation rate of its own mRNA in Escherichia coli: unusual sensitivity of the rne transcript to RNase E activity. Genes Dev. *9*, 84–96.

Joanny, G., Le Derout, J., Bréchemier-Baey, D., Labas, V., Vinh, J., Régnier, P., and Hajnsdorf, E. (2007). Polyadenylation of a functional mRNA controls gene expression in Escherichia coli. Nucleic Acids Res. *35*, 2494–2502.

Kaberdin, V.R. (2003). Probing the substrate specificity of Escherichia coli RNase E using a novel oligonucleotide-based assay. Nucleic Acids Res. *31*, 4710–4716.

Kim, V.N. (2005). MicroRNA biogenesis: coordinated cropping and dicing. Nat. Rev. Mol. Cell Biol. *6*, 376–385.

Kim, K.S., Manasherob, R., and Cohen, S.N. (2008). YmdB: a stress-responsive ribonuclease-binding regulator of E. coli RNase III activity. Genes Dev. *22*, 3497–3508.

Kime, L., Clarke, J.E., Romero A, D., Grasby, J.A., and McDowall, K.J. (2014). Adjacent single-stranded regions mediate processing of tRNA precursors by RNase E direct entry. Nucleic Acids Res. *42*, 4577–4589.

Kime, L., Vincent, H.A., Gendoo, D.M., Jourdan, S.S., Fishwick, C.W., Callaghan, A.J., and McDowall, K.J. (2015). The first small-molecule inhibitors of members of the ribonuclease E family. Sci. Rep. *5*, 8028.

Koo, J.T., Alleyne, T.M., Schiano, C.A., Jafari, N., and Lathem, W.W. (2011). Global discovery of small RNAs in Yersinia pseudotuberculosis identifies Yersinia-specific small, noncoding RNAs required for virulence. Proc. Natl. Acad. Sci. USA *108*, E709–E717.

Lalaouna, D., Carrier, M.C., Semsey, S., Brouard, J.S., Wang, J., Wade, J.T., and Massé, E. (2015). A 3′ external transcribed spacer in a tRNA transcript acts as a sponge for small RNAs to prevent transcriptional noise. Mol. Cell *58*, 393–405.

Lee, K., Zhan, X., Gao, J., Qiu, J., Feng, Y., Meganathan, R., Cohen, S.N., and Georgiou, G. (2003). RraA. a protein inhibitor of RNase E activity that globally modulates RNA abundance in E. coli. Cell *114*, 623–634.

Levine, E., and Hwa, T. (2008). Small RNAs establish gene expression thresholds. Curr. Opin. Microbiol. *11*, 574–579.

Li, Z., and Deutscher, M.P. (2002). RNase E plays an essential role in the maturation of Escherichia coli tRNA precursors. RNA *8*, 97–109.

Linder, P., Lemeille, S., and Redder, P. (2014). Transcriptome-wide analyses of 5′-ends in RNase J mutants of a gram-positive pathogen reveal a role in RNA maturation, regulation and degradation. PLoS Genet. *10*, e1004207.

Mackie, G.A. (1991). Specific endonucleolytic cleavage of the mRNA for ribosomal protein S20 of Escherichia coli requires the product of the ams gene in vivo and in vitro. J. Bacteriol. *173*, 2488–2497.

Mackie, G.A. (2013). RNase E: at the interface of bacterial RNA processing and decay. Nat. Rev. Microbiol. *11*, 45–57.

Madhugiri, R., Basineni, S.R., and Klug, G. (2010). Turn-over of the small non-coding RNA RprA in E. coli is influenced by osmolarity. Mol. Genet. Genomics *284*, 307–318.

Majdalani, N., Chen, S., Murrow, J., St John, K., and Gottesman, S. (2001). Regulation of RpoS by a novel small RNA: the characterization of RprA. Mol. Microbiol. *39*, 1382–1394.

Mandin, P., and Gottesman, S. (2010). Integrating anaerobic/aerobic sensing and the general stress response through the ArcZ small RNA. EMBO J. *29*, 3094–3107.

Massé, E., Escorcia, F.E., and Gottesman, S. (2003). Coupled degradation of a small regulatory RNA and its mRNA targets in Escherichia coli. Genes Dev. *17*, 2374–2383.

Mattick, J.S. (2004). RNA regulation: a new genetics? Nat. Rev. Genet. *5*, 316–323.

McClain, W.H., Guerrier-Takada, C., and Altman, S. (1987). Model substrates for an RNA enzyme. Science *238*, 527–530.

McDowall, K.J., Lin-Chao, S., and Cohen, S.N. (1994). A+U content rather than a particular nucleotide order determines the specificity of RNase E cleavage. J. Biol. Chem. *269*, 10790–10796.

McDowall, K.J., Kaberdin, V.R., Wu, S.W., Cohen, S.N., and Lin-Chao, S. (1995). Site-specific RNase E cleavage of oligonucleotides and inhibition by stem-loops. Nature *374*, 287–290.

Melamed, S., Peer, A., Faigenbaum-Romm, R., Gatt, Y.E., Reiss, N., Bar, A., Altuvia, Y., Argaman, L., and Margalit, H. (2016). Global mapping of small RNA-target interactions in bacteria. Mol. Cell *63*, 884–897.

Miyakoshi, M., Chao, Y., and Vogel, J. (2015a). Cross talk between ABC transporter mRNAs via a target mRNA-derived sponge of the GcvB small RNA. EMBO J. *34*, 1478–1492.

Miyakoshi, M., Chao, Y., and Vogel, J. (2015b). Regulatory small RNAs from the 3′ regions of bacterial mRNAs. Curr. Opin. Microbiol. *24*, 132–139.

Moll, I., Afonyushkin, T., Vytvytska, O., Kaberdin, V.R., and Bläsi, U. (2003). Coincident Hfq binding and RNase E cleavage sites on mRNA and small regulatory RNAs. RNA *9*, 1308–1314.

Morita, T., Maki, K., and Aiba, H. (2005). RNase E-based ribonucleoprotein complexes: mechanical basis of mRNA destabilization mediated by bacterial noncoding RNAs. Genes Dev. *19*, 2176–2186.

Otaka, H., Ishikawa, H., Morita, T., and Aiba, H. (2011). PolyU tail of rho-independent terminator of bacterial small RNAs is essential for Hfq action. Proc. Natl. Acad. Sci. USA *108*, 13059–13064.

Ow, M.C., and Kushner, S.R. (2002). Initiation of tRNA maturation by RNase E is essential for cell viability in E. coli. Genes Dev. *16*, 1102–1115.

Papenfort, K., and Vanderpool, C.K. (2015). Target activation by regulatory RNAs in bacteria. FEMS Microbiol. Rev. *39*, 362–378.

Papenfort, K., Said, N., Welsink, T., Lucchini, S., Hinton, J.C., and Vogel, J. (2009). Specific and pleiotropic patterns of mRNA regulation by ArcZ, a conserved, Hfq-dependent small RNA. Mol. Microbiol. *74*, 139–158.

Papenfort, K., Sun, Y., Miyakoshi, M., Vanderpool, C.K., and Vogel, J. (2013). Small RNA-mediated activation of sugar phosphatase mRNA regulates glucose homeostasis. Cell *153*, 426–437.

Papenfort, K., Espinosa, E., Casadesús, J., and Vogel, J. (2015). Small RNA-based feedforward loop with AND-gate logic regulates extrachromosomal DNA transfer in Salmonella. Proc. Natl. Acad. Sci. USA *112*, E4772–E4781.

Patel, A.M., and Dunn, S.D. (1992). RNase E-dependent cleavages in the 5′ and 3′ regions of the Escherichia coli unc mRNA. J. Bacteriol. *174*, 3541–3548.

Pfeiffer, V., Papenfort, K., Lucchini, S., Hinton, J.C., and Vogel, J. (2009). Coding sequence targeting by MicC RNA reveals bacterial mRNA silencing downstream of translational initiation. Nat. Struct. Mol. Biol. *16*, 840–846.

Régnier, P., and Hajnsdorf, E. (1991). Decay of mRNA encoding ribosomal protein S15 of Escherichia coli is initiated by an RNase E-dependent endonucleolytic cleavage that removes the 3′ stabilizing stem and loop structure. J. Mol. Biol. *217*, 283–292.

Roy, M.K., and Apirion, D. (1983). Purification and properties of ribonuclease E, an RNA-processing enzyme from Escherichia coli. Biochim. Biophys. Acta *747*, 200–208.

Saramago, M., Bárria, C., Dos Santos, R.F., Silva, I.J., Pobre, V., Domingues, S., Andrade, J.M., Viegas, S.C., and Arraiano, C.M. (2014). The role of RNases in the regulation of small RNAs. Curr. Opin. Microbiol. *18*, 105–115.

Sauer, E., and Weichenrieder, O. (2011). Structural basis for RNA 3′-end recognition by Hfq. Proc. Natl. Acad. Sci. USA *108*, 13065–13070.

Schifano, J.M., Vvedenskaya, I.O., Knoblauch, J.G., Ouyang, M., Nickels, B.E., and Woychik, N.A. (2014). An RNA-seq method for defining endoribonuclease cleavage specificity identifies dual rRNA substrates for toxin MazF-mt3. Nat. Commun. *5*, 3538.

Smirnov, A., Förstner, K.U., Holmqvist, E., Otto, A., Günster, R., Becher, D., Reinhardt, R., and Vogel, J. (2016). Grad-seq guides the discovery of ProQ as a major small RNA-binding protein. Proc. Natl. Acad. Sci. USA *113*, 11591–11596.

Soper, T., Mandin, P., Majdalani, N., Gottesman, S., and Woodson, S.A. (2010). Positive regulation by small RNAs and the role of Hfq. Proc. Natl. Acad. Sci. USA *107*, 9602–9607.

Storz, G., Vogel, J., and Wassarman, K.M. (2011). Regulation by small RNAs in bacteria: expanding frontiers. Mol. Cell *43*, 880–891.

Tree, J.J., Granneman, S., McAteer, S.P., Tollervey, D., and Gally, D.L. (2014). Identification of bacteriophage-encoded anti-sRNAs in pathogenic Escherichia coli. Mol. Cell *55*, 199–213.

Viegas, S.C., Pfeiffer, V., Sittka, A., Silva, I.J., Vogel, J., and Arraiano, C.M. (2007). Characterization of the role of ribonucleases in Salmonella small RNA decay. Nucleic Acids Res. *35*, 7651–7664.

Viegas, S.C., Mil-Homens, D., Fialho, A.M., and Arraiano, C.M. (2013). The virulence of Salmonella enterica Serovar Typhimurium in the insect model galleria mellonella is impaired by mutations in RNase E and RNase III. Appl. Environ. Microbiol. *79*, 6124–6133.

Vogel, J., and Luisi, B.F. (2011). Hfq and its constellation of RNA. Nat. Rev. Microbiol. *9*, 578–589.

Wagner, E.G. (2013). Cycling of RNAs on Hfq. RNA Biol. *10*, 619–626.

Wagner, E.G., and Romby, P. (2015). Small RNAs in bacteria and archaea: who they are, what they do, and how they do it. Adv. Genet. *90*, 133–208.

Westermann, A.J., Förstner, K.U., Amman, F., Barquist, L., Chao, Y., Schulte, L.N., Müller, L., Reinhardt, R., Stadler, P.F., and Vogel, J. (2016). Dual RNA-seq unveils noncoding RNA functions in host-pathogen interactions. Nature *529*, 496–501.

Worrall, J.A., Górna, M., Crump, N.T., Phillips, L.G., Tuck, A.C., Price, A.J., Bavro, V.N., and Luisi, B.F. (2008). Reconstitution and analysis of the multienzyme Escherichia coli RNA degradosome. J. Mol. Biol. *382*, 870–883.

Zeidler, M.P., Tan, C., Bellaiche, Y., Cherry, S., Häder, S., Gayko, U., and Perrimon, N. (2004). Temperature-sensitive control of protein activity by conditionally splicing inteins. Nat. Biotechnol. *22*, 871–876.

Zhang, A., Wassarman, K.M., Rosenow, C., Tjaden, B.C., Storz, G., and Gottesman, S. (2003). Global analysis of small RNA and mRNA targets of Hfq. Mol. Microbiol. *50*, 1111–1124.

Molecular Cell, Volume *65*

Supplemental Information

In Vivo Cleavage Map Illuminates the Central Role of RNase E in Coding and Non-coding RNA Pathways

Yanjie Chao, Lei Li, Dylan Girodat, Konrad U. Förstner, Nelly Said, Colin Corcoran, Michał Śmiga, Kai Papenfort, Richard Reinhardt, Hans-Joachim Wieden, Ben F. Luisi, and Jörg Vogel

Supplemental discussion on molecular dynamics simulation

The identified cleavage sites for sRNA biogenesis suggest that RNase E recognizes its target site via a consensus motif (Fig. 2F) with strong selection for a uridine two nucleotides downstream of the cleavage site (U_{+2}). X-ray crystallographic structures of RNase E bound to a non-cognate substrate revealed that the base two nucleotides downstream of the cleavage site stacks with amino acid residues Phe_{67} and Lys_{112} of RNase E (Fig. S7A; (Callaghan et al., 2005; Mackie, 2013). The fact that the scissile phosphate is located two nucleotides upstream of the conserved uridine in the recognition motif suggests that RNase E recognizes the cleavage site using a two-nucleotide (U_{+2}) ruler mechanism. This two-nucleotide ruler mechanism is facilitated via the correct positioning of a water molecule (coordinated by an enzyme-bound magnesium ion) for attack on the phosphodiester bond at this position. However, the available structures do not provide information regarding the mechanism leading to uridine recognition. This is in part due to the fact that the available RNase E structures contain a non-cognate RNA trapped at pre-cleavage state (Callaghan et al., 2005). Based on this we hypothesized that upon binding to a cognate, cleavable RNA substrate, RNase E may undergo a conformational rearrangement of its catalytic site that requires the presence of a uridine two nucleotides downstream of the scissile phosphate. To investigate this, we carried out all-atom molecular dynamics simulations exploring the structural dynamics of RNase E in the presence of a cognate (U_{+2}) and a non-cognate (G_{+2}) substrate. Our simulations reveal that the cognate U_{+2} not only stacks with Phe_{67}, but it is also tightly bound in a putative uracil binding pocket consisting of the $Lys_{112}Gly_{113}Ala_{114}Ala_{115}$ ($^{112}KGAA^{115}$) loop in the S1 domain of RNase E (Fig. S7A-B). The peptide-backbone amides of this loop are oriented towards the U_{+2} forming a positively charged cleft where Ala_{114} and Gly_{113} can form hydrogen bonds to the O^4 of the U_{+2} base, ultimately providing a feature that allows to discriminate between uracil and guanine bases.

Interestingly, during the course of the simulations we observed a repositioning of the Lys_{112} side chain which only occurs in the presence of the cognate U_{+2} substrate (Fig. S7 A-C). In our simulation of the RNase E-cognate RNA complex, Lys_{112} adopts two distinct conformations that are different from the previously proposed position (Callaghan et al., 2005). In these conformations the side chain of Lys_{112} forms a transient interaction with the phosphate one nucleotide downstream (23% of the time within 4 Å of a phosphate oxygen) and a more stable conformation interacting with Phe_{76} (75% of the time within 4 Å of Phe_{67}), essentially closing the uracil binding pocket and trapping the U_{+2}. It is not surprising that this conformation has not previously been observed in the available non-cognate structures as the non-cognate G_{+2} base, contains an additional bulky imidazole ring that sterically prevents the sampling of this conformation by Lys_{112}. Constant with this, Lys_{112} is not resolved in the available X-ray crystallographic structures and is likely to be highly flexible in the non-cognate complex (Callaghan et al., 2005). The closed uracil pocket in conjunction with the reposition of Lys_{112} shift the position of A_{+1} and U_{+2} nucleotide bases leading to a distortion of the phosphodiester backbone (Fig. S7D & Fig. 7B). The distortion at the scissile phosphate likely proceeds through a pseudo trigonal bipyramidal geometry (or 'five-oxygen interacting state') optimal for the in-line nucleophilic attack of an activated water molecule mediated by divalent metal ions (Martick and Scott, 2006; Oivanen et al., 1998; Torres and Bruice, 1998). This geometry is not formed in the simulation with RNA alone (Fig. S7F), or with RNase E-RNAs complex containing the non-cognate G_{+2} in RNA (Fig. S7E), as the closing of the uracil pocket via the Lys_{112} is not possible due to the larger guanine base. The subsequent cleavage reaction will result in fragments with the phosphate

on the 5' end and a fragment with the OH group on the 3' end (Fig. S7D), consistent with the well-established features of RNase E cleavage (Mackie, 2013; Misra and Apirion, 1979).

Overall, our data suggest that RNase E is able to specifically recognize a uracil in substrate RNA and mediates cleavage using a U_{+2} ruler and cut mechanism (Fig. 7B & Fig. S7D). Notably, the residues critical for forming the uracil pocket are conserved in all RNase E homologs. The alanine substitution of the Lys_{112} has previously been shown to abolish cleavage activity also for cognate substrates, suggesting that the proposed mechanism is conserved and required for RNase E activity (Callaghan et al., 2005). This is further supported by previous *in vitro* observations, which show that substrate RNAs with G_{+2} can bind to RNase E but are not cleaved (Callaghan et al., 2005; Kaberdin, 2003).

Figure S1

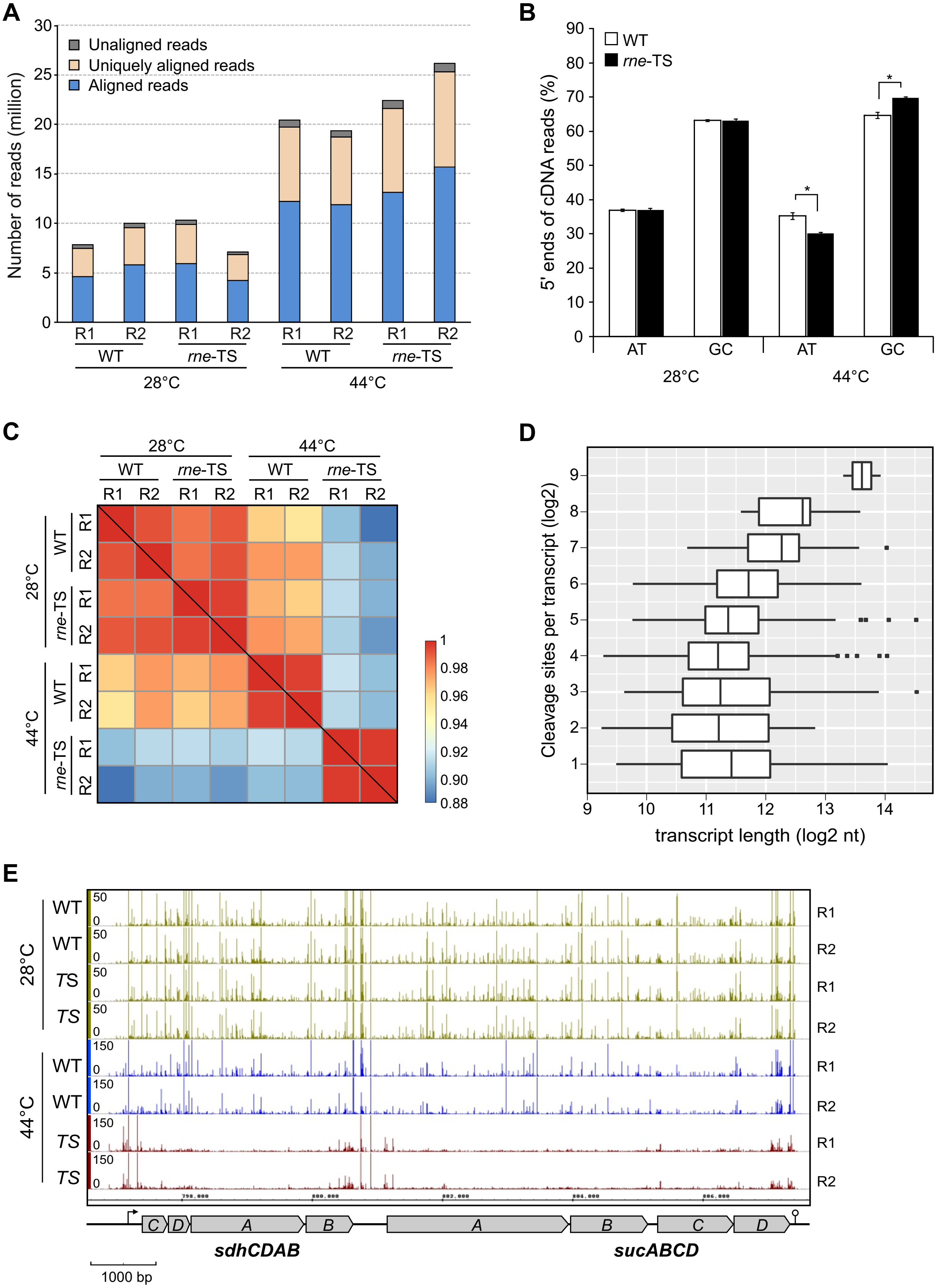
A
Unaligned reads
Uniquely aligned reads
Aligned reads
Number of reads (million)
R1
R2
WT
rne-TS
28°C
44°C
B
WT
rne-TS
5' ends of cDNA reads (%)
AT
GC
28°C
44°C
C
28°C
44°C
WT
rne-TS
R1
R2
D
Cleavage sites per transcript (log2)
transcript length (log2 nt)
E
28°C
44°C
WT
TS
R1
R2
sdhCDAB
sucABCD
1000 bp

Figure S2

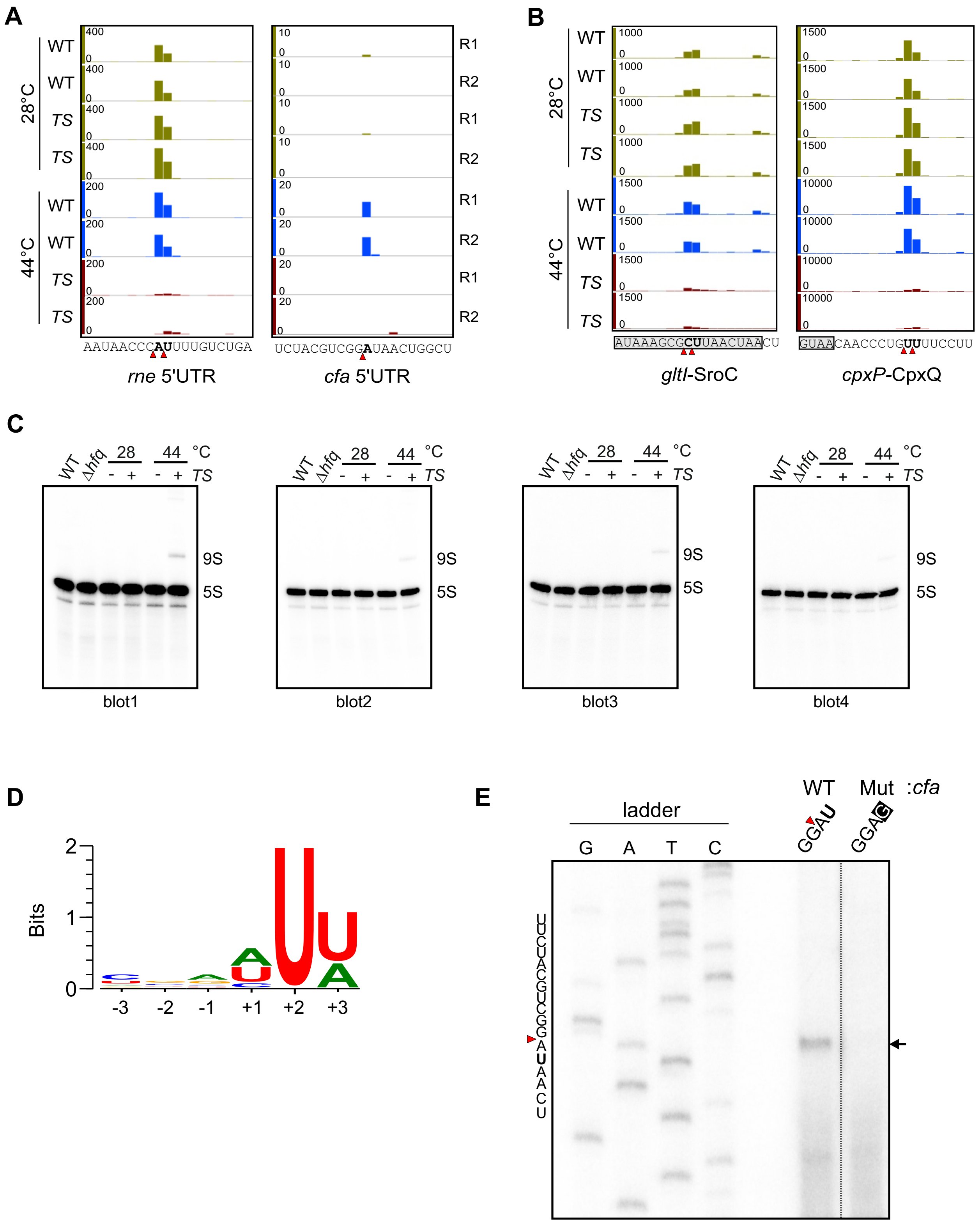

A
28°C
44°C
WT
TS
R1
R2
AAUAAACCCAUUUUGUCUGA
UCUACGUCGGAUAACUGGCU
rne 5'UTR
cfa 5'UTR
B
AUAAAGCGCUUAACUAACU
GUAACAACCCUGUUUUCCUU
gltI-SroC
cpxP-CpxQ
C
WT
Δhfq
28
44
°C
TS
9S
5S
blot1
blot2
blot3
blot4
D
Bits
-3
-2
-1
+1
+2
+3
E
ladder
G
A
T
C
WT
Mut
:cfa
GGAU
GGAG
UUCUACGUCGGAUAACU

Figure S3

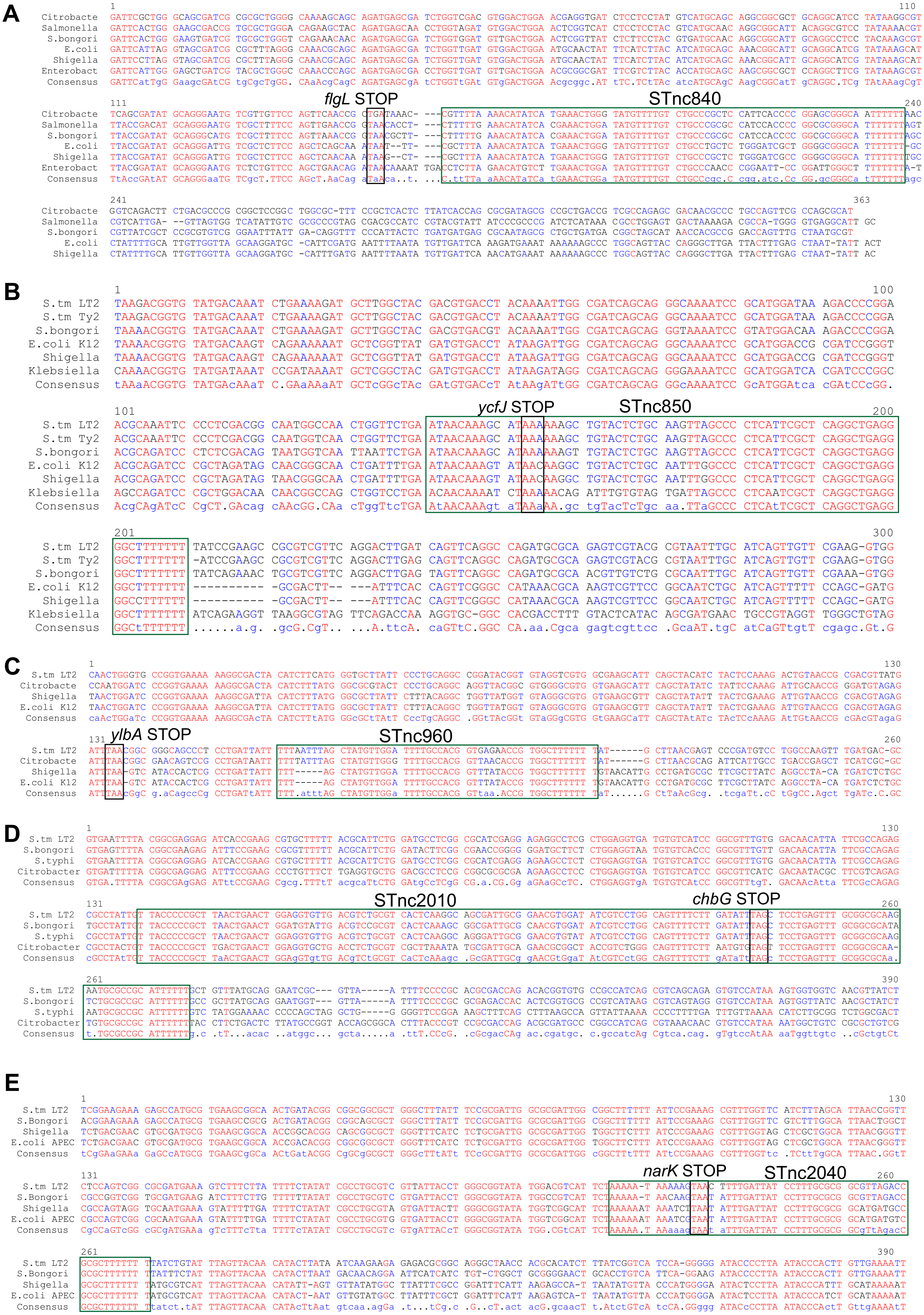
A
Citrobacte
Salmonella
S.bongori
E.coli
Shigella
Enterobact
Consensus
flgL STOP
STnc840
B
S.tm LT2
S.tm Ty2
S.bongori
E.coli K12
Shigella
Klebsiella
Consensus
ycfJ STOP
STnc850
C
S.tm LT2
Citrobacte
Shigella
E.coli K12
Consensus
ylbA STOP
STnc960
D
S.tm LT2
S.bongori
S.typhi
Citrobacter
Consensus
STnc2010
chbG STOP
E
S.tm LT2
S.Bongori
Shigella
E.coli APEC
Consensus
narK STOP
STnc2040

Figure S4

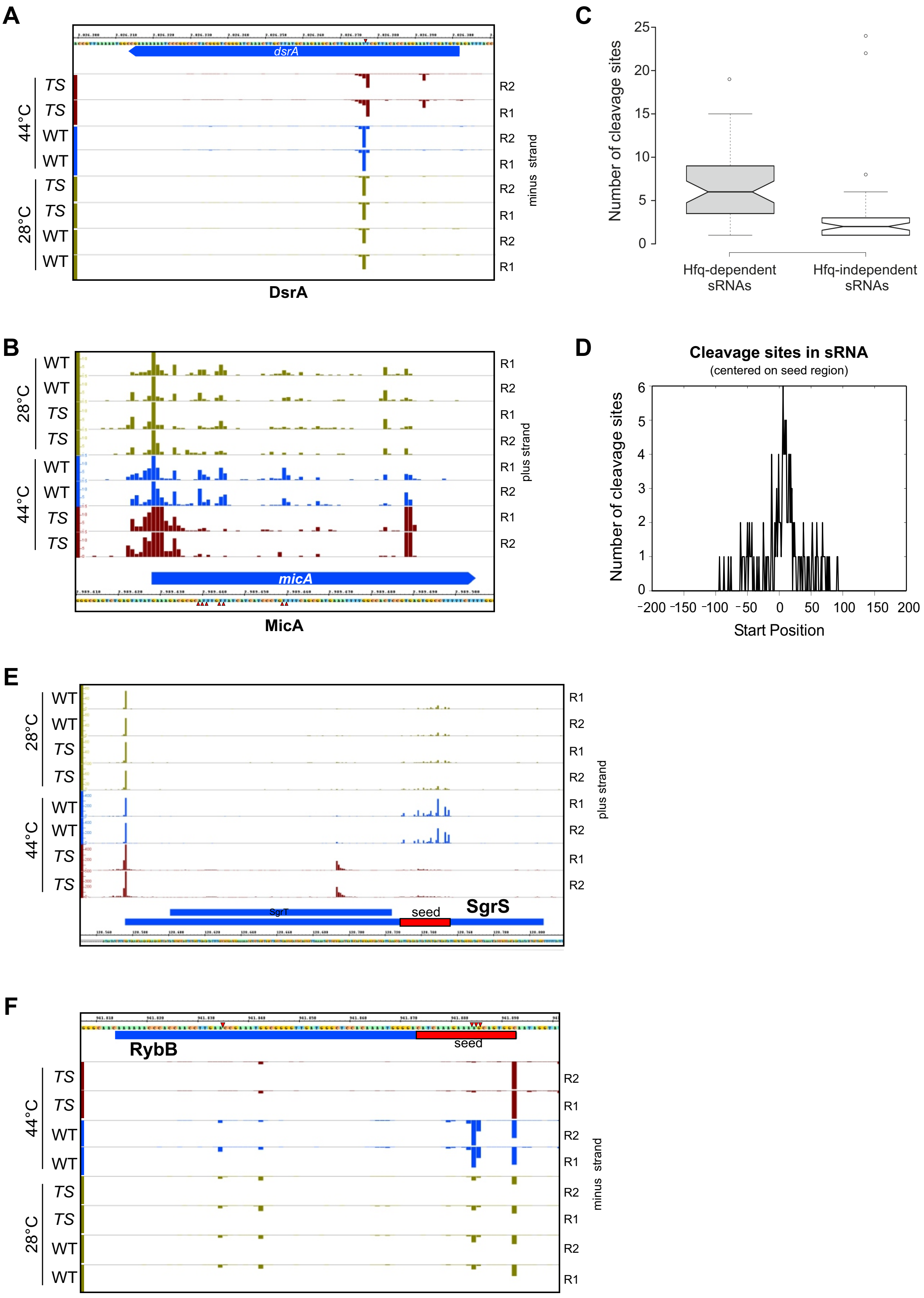
A
dsrA
TS
TS
WT
WT
TS
TS
WT
WT
44°C
28°C
R2
R1
R2
R1
R2
R1
R2
R1
minus strand
DsrA
B
WT
WT
TS
TS
WT
WT
TS
TS
28°C
44°C
R1
R2
R1
R2
R1
R2
R1
R2
plus strand
micA
MicA
C
Number of cleavage sites
25
20
15
10
5
0
Hfq-dependent sRNAs
Hfq-independent sRNAs
D
Cleavage sites in sRNA
(centered on seed region)
Number of cleavage sites
6
5
4
3
2
1
0
-200 -150 -100 -50 0 50 100 150 200
Start Position
E
WT
WT
TS
TS
WT
WT
TS
TS
28°C
44°C
R1
R2
R1
R2
R1
R2
R1
R2
plus strand
SgrT
seed
SgrS
F
RybB
seed
TS
TS
WT
WT
TS
TS
WT
WT
44°C
28°C
R2
R1
R2
R1
R2
R1
R2
R1
minus strand

Figure S5

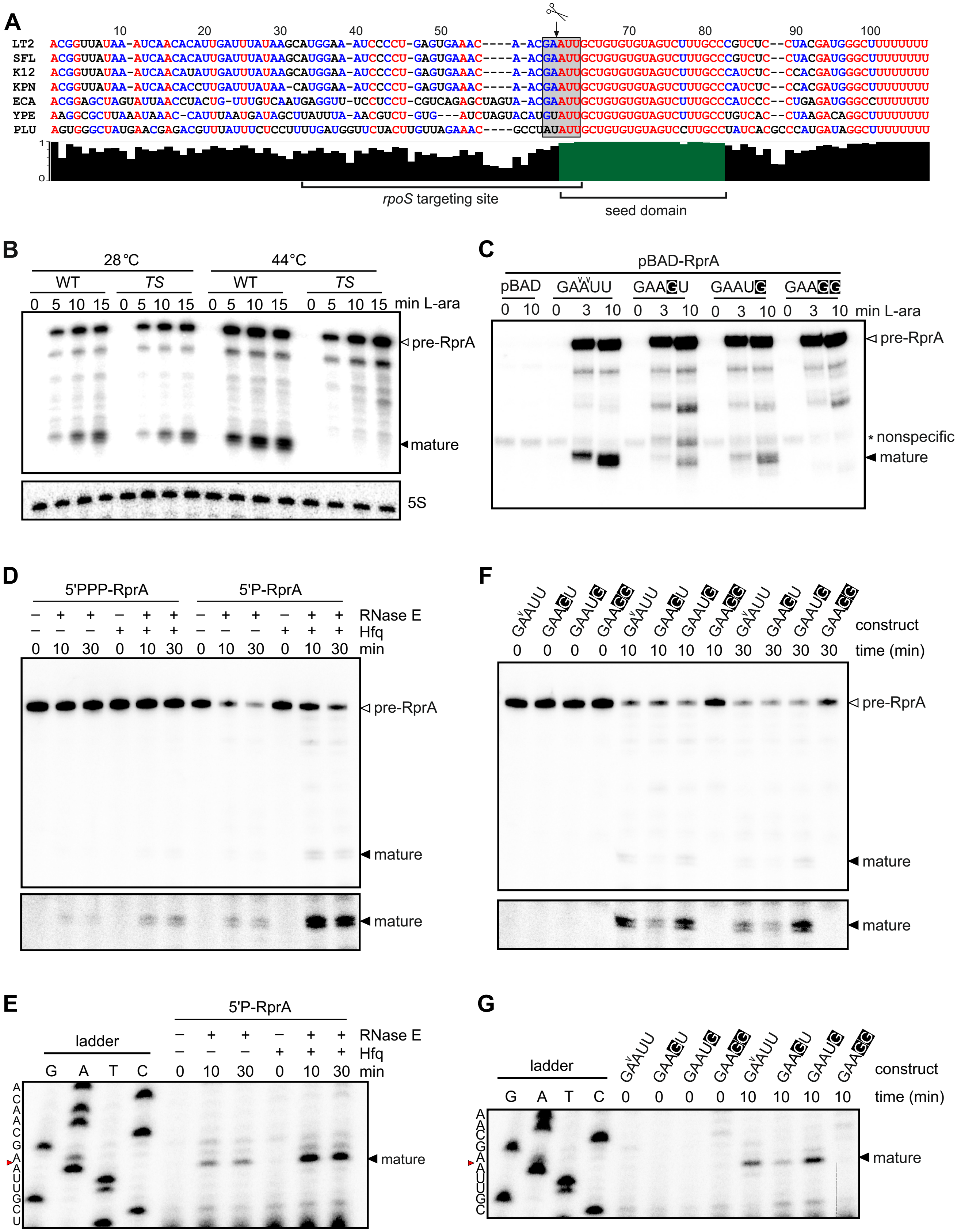
A
rpoS targeting site
seed domain
B
28 °C
44°C
WT
TS
min L-ara
pre-RprA
mature
5S
C
pBAD-RprA
pBAD
min L-ara
pre-RprA
nonspecific
mature
D
5'PPP-RprA
5'P-RprA
RNase E
Hfq
min
pre-RprA
mature
F
construct
time (min)
pre-RprA
mature
E
ladder
5'P-RprA
RNase E
Hfq
min
mature
G
ladder
construct
time (min)
mature

Figure S6

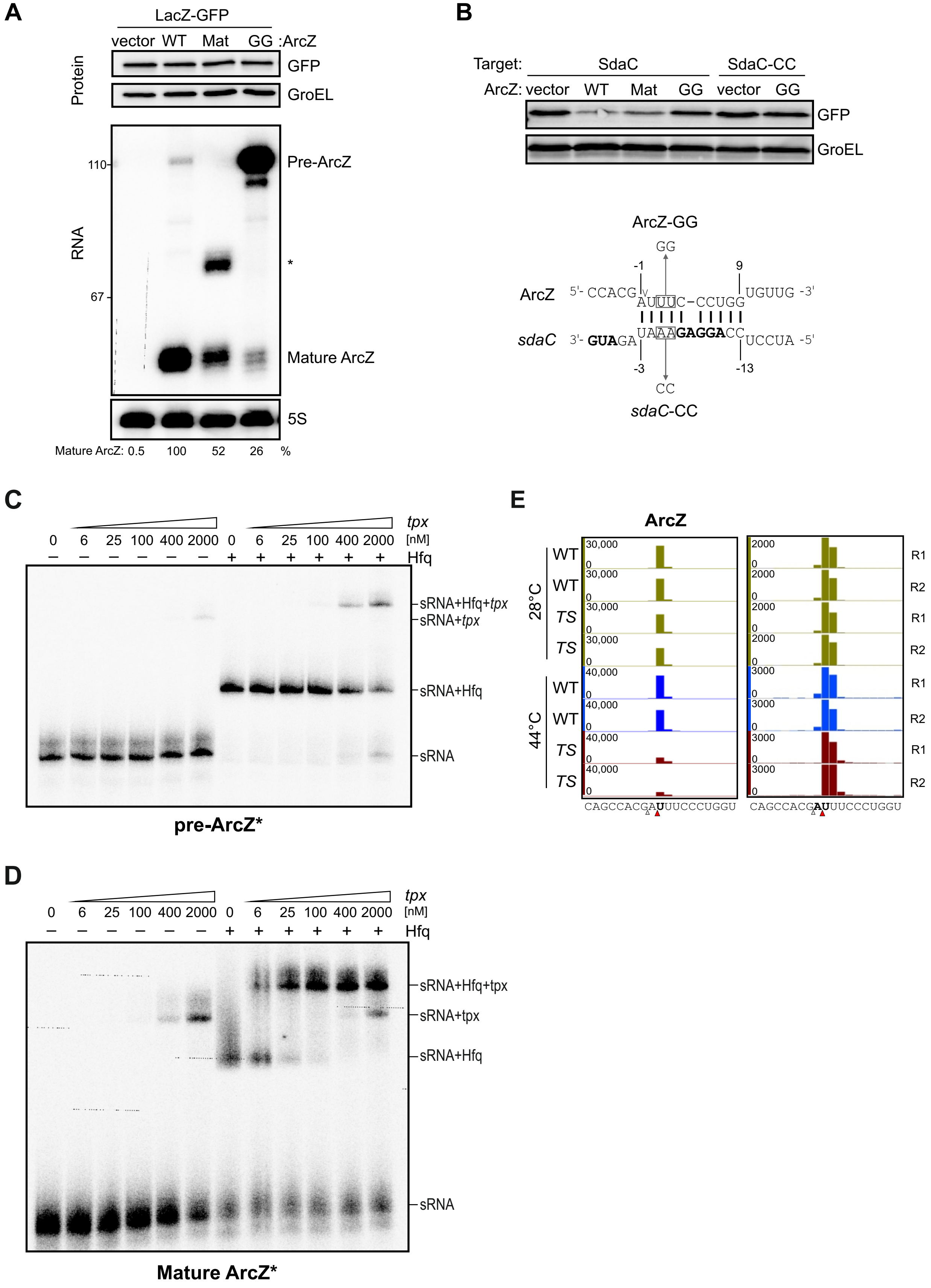

A
LacZ-GFP
vector WT Mat GG :ArcZ
Protein
GFP
GroEL
RNA
110
67
Pre-ArcZ
*
Mature ArcZ
5S
Mature ArcZ: 0.5 100 52 26 %
B
Target: SdaC SdaC-CC
ArcZ: vector WT Mat GG vector GG
GFP
GroEL
ArcZ-GG
GG
-1
9
ArcZ 5'- CCACG AUUUC-CCUGG UGUUG -3'
sdaC 3'- GUAGA UAAAGAGGACC UCCUA -5'
-3
-13
CC
sdaC-CC
C
tpx
0 6 25 100 400 2000 0 6 25 100 400 2000 [nM]
Hfq
sRNA+Hfq+tpx
sRNA+tpx
sRNA+Hfq
sRNA
pre-ArcZ*
D
tpx
0 6 25 100 400 2000 0 6 25 100 400 2000 [nM]
Hfq
sRNA+Hfq+tpx
sRNA+tpx
sRNA+Hfq
sRNA
Mature ArcZ*
E
ArcZ
28°C
44°C
WT
WT
TS
TS
WT
WT
TS
TS
30,000
40,000
2000
3000
R1
R2
CAGCCACGAUUUCCCUGGU

Figure S7

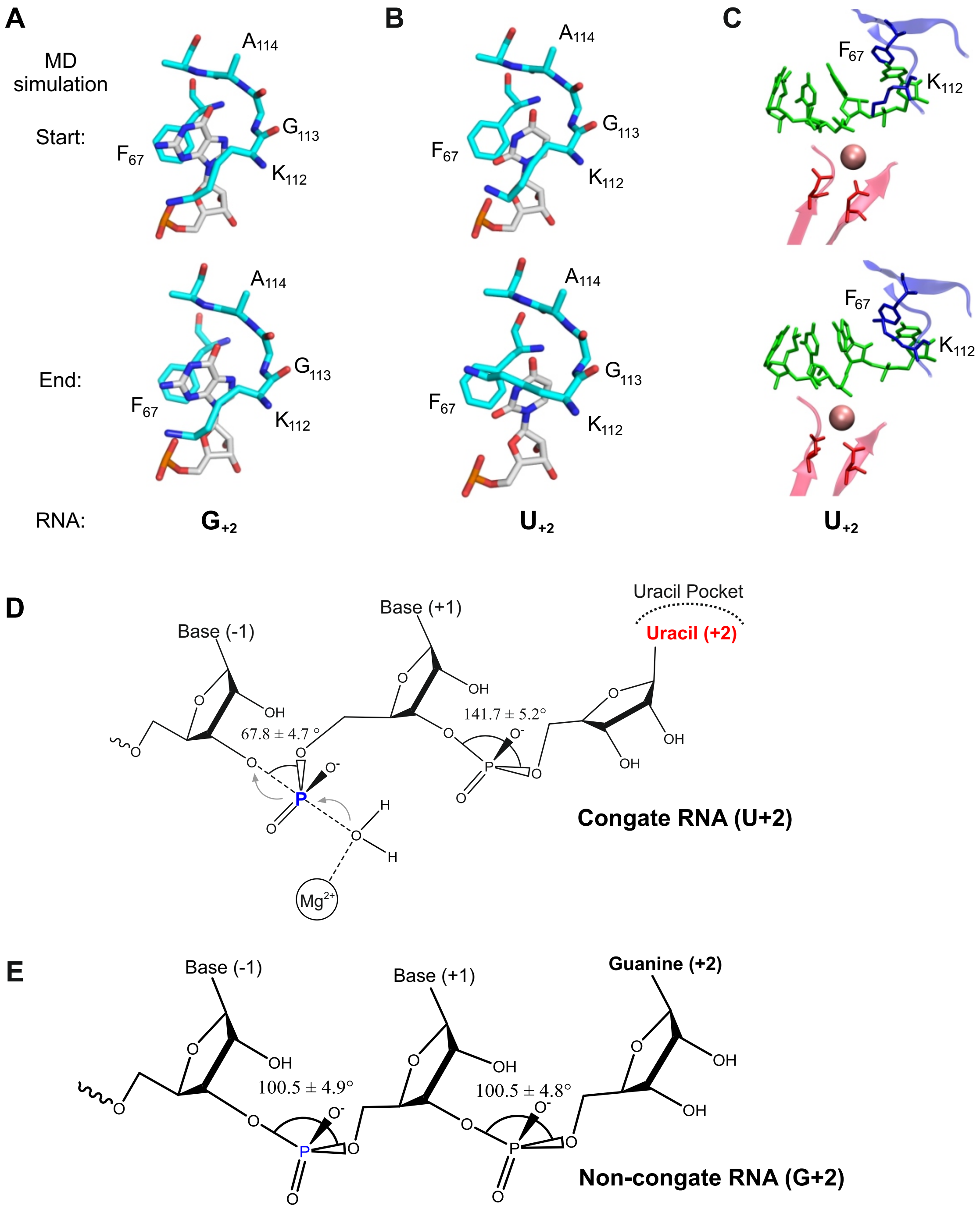

F

	Cognate U_{+2}	Non-Cognate G_{+2}	Cognate U_{+2} (RNA Alone)
O5' – P – O3'	67.8 ± 4.7°	100.5 ± 4.9°	100.6 ± 4.8°
O5' – P – OP1	110.1 ± 5.4°	109.4 ± 5.2°	109.5 ± 5.2°
O5' – P – OP2	109.6 ± 5.5°	111.4 ± 5.2°	109.4°± 5.3
O3' – P – OP1	120.7 ± 4.4°	109.1 ± 5.3°	110.1 ± 4.9°
O3' – P – OP2	120.0 ± 4.9°	110.4 ± 4.9°	110.0 ± 5.2°
OP1 – P – OP2	115.1 ± 4.6°	114.6 ± 5.1°	115.4 ± 5.1°

Supplemental Figure Legends:

Figure S1. Overview of TIER-seq results. Related to Figure 1 and Figure 2.

A) Bar plot showing the total number of raw cDNA reads (million) obtained for all the samples. R1 and R2 are two biological replicates. The unaligned reads are shown in grey; the rest reads have been aligned to the *Salmonella* genome. Among these aligned sequences, uniquely aligned reads (mostly non-rRNA/tRNA reads) are indicated in light orange.

B) The inactivation of RNase E leads to a reduction of cDNA reads with 5' A/T base. Bars indicate the relative amount (%) of reads that have A/T or G/C bases at the 5' end in a cDNA library. The error bars indicate standard deviation. * indicates $p<0.05$, two-tailed Student's *t*-test.

C) Samples are clustered based on the similarity of 5' ends profile. The reads counts of all the detected 5' end positions in a cDNA library are compared with that of another cDNA library. The Pearson correlation coefficient is plotted, and the scale bar is shown.

D) The number of cleavage sites per transcript was plotted against the respective gene (transcript) length.

E) The reads distribution in the polycistronic *sdh-suc* operons showing numerous RNase E cleavage-derived 5' ends. IGB screenshot is shown; R1 and R2 indicate two biological replicates.

Figure S2. Examples of mapped RNase E cleavage sites. Related to Figure 1, Figure 2 and Figure 3.

A) The mapped RNase E cleavage sites in the *rne* 5'UTR and the *cfa* 5'UTR, which match the previously determined sites. We note that the RNase E cleavage in *cfa* may be affected by temperature, as more cleavage products were detected at the elevated temperature (44°C).

B) The mapped RNase E cleavage sites in the 3'UTR of *cpxP* and *gltI*, which are responsible for the biogenesis of 3'UTR-derived CpxQ and SroC sRNA, respectively. The shaded box indicates the open reading frame of the respective host gene (*cpxP, gltI*). The arrowheads show the mapped cleavage site, or the 5' end of the resulting sRNAs.

C) 5S rRNA loading controls for the northern blots shown in the Fig. 3 and Fig. 4. Note that the 9S rRNA signals confirms the transient inactivation of RNase E at 44˚C (rne-TS).

D) RNase E sequence logo derived from all the cleavage sites in 3'UTR-derived sRNAs.

E) Mutation of U_{+2} abolishes RNase E cleavage in mRNA. The RNase E cleavage sites in the 5' UTR of *cfa* mRNA was determined using primer extension. WT *cfa* or mutant *cfa* was constitutively expressed from pKF31 or pYC311. Total RNA was isolated and subjected to primer extension analysis using 5' end-labeled oligo pZE-XbaI. The determined RNase E cleavage site is indicated by arrow.

Figure S3. Sequence alignment of new 3'UTR-derived sRNAs identified in this study. Related to Figure 3.

A) Sequence alignment of STnc840 in the *flgL* 3'UTR. The green box indicates sRNA sequence and the black box indicates the stop codon of its host mRNA.

B) Sequence alignment of STnc850 in the *ycfJ* 3'UTR.

C) Sequence alignment of STnc960 in the *ylbA* 3'UTR.

D) Sequence alignment of STnc2010 in the *chbG*/*celG* 3'UTR.

E) Sequence alignment of STnc2040 in the *narK* 3'UTR.

Figure S4. Analysis of RNase E cleavage sites in Hfq-dependent sRNAs. Related to Figure 3.

A) Screenshot showing all the 5' ends of reads within *dsrA* locus. The arrowhead indicates the mapped RNase E site. Data from both replicates are shown.

B) Screenshot showing all the 5'ends within *micA* locus. The arrowheads indicate the mapped RNase E sites. Data from both replicates are shown.

C) The number of identified cleavage sites in Hfq-dependent sRNAs are higher than that in Hfq-independent sRNAs. Center lines show the medians; box limits indicate the 25th and 75th percentiles; whiskers extend 1.5 times the interquartile range from the 25th and 75th percentiles, outliers are represented by dots.

D) Meat-gene analysis showing the distribution of cleavage sites within sRNAs. The position on the x-aixs is relative to the seed of sRNAs.

E) Screenshot showing all the 5' ends within *sgrS* locus. The defined seed region of SgrS is highlighted. Data from both replicates are shown.

F) Screenshot showing all the 5' ends within *rybB* locus. The defined seed region in the 5' end of RybB is highlighted, and the mapped RNase E cleavage sites are indicated by arrow heads. Data from both replicates are shown.

Figure S5. RNase E mediates the maturation of RprA small RNA. Related to Figure 4 and Figure 5.

A) Sequence alignment of the RprA small RNA. The RNase E cleavage site is indicated by a scissor. The sequence conservation score is shown below the alignment. LT2: S*almonella* Typhimurium LT2. SFL: *Shigella flexneri*. K12: *Escherichia coli* K-12. KPN: *Klebsiella pneumoniae*. ECA: *Erwinia carotovora*. YPE, *Yersinia pestis*. PLU: *Photorhabdus luminescens*.

B) The processing of RprA is dependent on RNase E. In both rne^{TS} (TS) and WT control strains, the RprA sRNA was pulse-expressed from pKP15-13 by the addition of 0.2% L-arabinose for 0, 5, 10, or 15 min. RprA was detected by northern blotting using 5'end-labeled oligo JVO-4049. 5S served as a loading control.

C) Mutations at U_{+2} abolishes RprA maturation in vivo. WT RprA and its variants were induced from pBAD plasmids by the addition of 0.2% L-arabinose for 0, 3, or 10 min. RprA was detected by northern blotting using 5'end-labeled oligo JVO-4049.

D) RprA was processed by RNase E in the presence of Hfq in vitro. The RprA sRNA with either 5'PPP or 5'P (TAP treated) was incubated with equimolar ratio of RNase E in the presence or absence of Hfq in vitro. The reactions were stopped at the indicated time point and separated by a denaturing 6% PAA/7M urea gel. RprA was detected by northern blotting using 5'end-labeled oligo JVO-4049.

E) Mapping the *in vitro* cleavage sites by primer extension using RNA from (D). The arrowhead indicates the RNase E cleavage sites in RprA determined by primer extension using 5'end-labeled oligo JVO-5902.

F) The processing of RprA is dependent on the critical uridine at +2 position. The RprA variants (with 5'P) were incubated with Hfq and RNase E for the indicated time period. The reactions were separated by a denatured 6% PAA/7M urea gel, and RprA was detected using 5'end-labeled oligo JVO-4049. The mature RprA fragment was shown in the lower panel with increased exposure.

G) Mapping the in vitro cleavage sites using primer extension. RNA from (F) was subjected to primer extension analysis using 5'end-labeled oligo JVO-5902. The arrowhead indicates the determined RNase E cleavage sites in RprA.

Figure S6. The maturation of ArcZ is required for target binding and regulation. Related to Figure 6.

A) Expression of ArcZ variants *in vivo*. *Salmonella* Δ*arcZ* strain (JVS-0082) containing LacZ-*gfp* control plasmid (pXG-1) and the sRNA plasmid vector (pKP8-35), full-ArcZ (pKP4-13), short-ArcZ (pKP31-5), or ArcZ* (pYC250) were grown to OD_{600} of 2.0 in LB medium (~3h) in the presence of 0.2% L-arabinose. The expression of GFP was analyzed by Western blotting and the expression of ArcZ sRNA was analyzed by northern blotting using 5'-end labeled oligo JVO-4208. The quantification of the mature ArcZ level is shown below (%).

B) Regulation of SdaC translational fusions. The expression of SdaC-GFP reporter (pKP-102) and SdaC*-GFP reporter (pYC348) was analyzed as in (A). The quantification of GFP levels is shown (%). The introduced mutations and the base pairing interactions are shown to the right (Papenfort et al., 2009).

C) EMSA analysis of the interactions between mature ArcZ sRNA and *tpx* mRNA. ~5 nM 5'-end-labeled mature wt ArcZ sRNA was incubated with increasing concentrations of the unlabeled *tpx* mRNA fragment (0, 6, 25, 100, 400, 2000 nM) at 37°C for 10 min, in the absence (-) or presence (+) of 40 nM Hfq.

D) EMSA analysis of the interactions between pre-ArcZ sRNA and *tpx* mRNA. ~5 nM 5'-end-labeled pre-ArcZ sRNA was incubated with increasing concentration of the unlabeled *tpx* mRNA fragment (0, 6, 25, 100, 400, 2000 nM) at 37°C for 10 min, in the absence (-) or presence (+) of 40 nM Hfq.

E) IGB screenshot showing the major (red arrowhead) and minor (open arrowhead) RNase E cleavage sites mapped in ArcZ.

Figure 7. Molecular Dynamics simulation reveals conformation changes during the RNase E-cognate RNA recognition facilitating cleavage of substrate RNA. Related to Figure 7.

A) Non-cognate RNA sequence bound to RNase E. At the start of the simulation, Lys_{112} coordinates both the +1 and scissile phosphates, and this conformation is maintained over the course of the simulation. RNA is colored in white, and the RNase E protein residues are colored in cyan. (top) RNA backbone at the beginning of the simulation, (bottom) RNA backbone at the end of the simulation.

B, C) Cognate RNA sequence bound to RNase E. Lys_{112} coordinates the +1 and scissile phosphates at the start of the simulation similar to (A). Over the course of the simulation, Lys_{112} adopts a conformation in which it has additional stacking potential with Phe_{67}. This conformation allows stacking of the Lys_{112} side chain with Phe_{67} and the cognate U_{+2} base present in the uracil pocket (C), closing the pocket around the base. Pink sphere, Mg^{2+} ion. (top) RNA backbone at the beginning of the simulation, (bottom) RNA backbone at the end of the simulation.

D) The conformation of RNA with cognate RNA with U_{+2}, revealed by molecular dynamics simulations. Upon recognition of the U_{+2} in the substrate RNA, the conformation of RNA-backbone 2 nt upstream of uridine is predicted to become distorted in a manner that may favor approach of the scissile phosphate to a pseudo-trigonal bipyramidal transition state geometry (90º) for in-line attack of a water molecule, consistent with the U_{+2} ruler mechanism.

E) The conformation of RNA with non-cognate G_{+2}. Different from the cognate RNA with U_{+2}, this geometry is maintained throughout the course of simulation, representing a stable bound state of RNA in the RNase E crystal (Callaghan et al., 2005).

F) Geometry of the Scissile Phosphate. Only the cognate RNA bound to RNase E adopts the distorted O5'-P-O3' angle that facilitates the in-line attack of a water molecule. The results are derived from three independent replicates, and the respective standard deviation is shown.

Supplemental Tables:

Table S1. List of the identified RNase E cleavage sites and sequences. Related to Figure 1 and Figure 2.

Table S2. The RPKM values of genes expressed at 44°C. Related to Figure 1.

Table S3. Essential genes and virulence-associated genes containing RNase E cleavage sites. Related to Figure 2.

Table S4. Genes with cleavage sites in the 5'UTR. Related to Figure 2.

Table S5. List of 3'UTR-sRNA candidates. Related to Figure 3.

Table S6. Small RNAs containing RNase E cleavage sites. Related to Figure 2 and Figure 3.

Table S7. Temperature upshift induced changes in WT samples. Related to Figure 1.

See the EXCEL files.

Supplemental Experimental Procedures

Bacterial strains and growth

The bacterial strains, plasmids, oligonucleotides used in this study are included in the supplemental lists. *Salmonella enterica* strains JVS-7000 ((*rluC-rne*)IG:*cat*/*rne*-3071, MA9816) and JVS-6999 (*rluC-rne*)IG:*cat*, MA9292) from the Bossi lab (Figueroa-Bossi et al., 2009) were used as the rne^{TS} and its isogenic WT strains. Bacteria were grown in LB-Lennox medium to an OD_{6oo} of 2.0 at 28°C with 220 rpm agitation, and were shifted to 44°C in a water bath and continue to grow for 30 min prior to RNA extraction. Experiments with other bacterial strains were performed at 37°C with 220 rpm agitation. A final concentration of 0.02% of L-arabinose was used to induce sRNA expression from pBAD vectors.

Total RNA extraction

Bacterial cultures corresponding to ~4 OD_{600} were collected and mixed with 0.2 vol. ice-cold STOP solution (95 % Ethanol, 5 % Phenol). Total RNA was isolated following the hot phenol protocol in order to preserve the RNA integrity. In brief, bacterial cells were resuspended in 600 µl of lysozyme solution (0.5 mg/ml, pH 8.0) and 60 µl of 10% SDS, and then incubated at 64°C for 1-2 minutes to lyse cells. After adding 66 µl of 3 M NaOAc (pH 5.2), the cell lysis was extracted by 750 µl phenol (Carl-Roth, #A980.3) and followed by another extraction using 750 µl chloroform. The supernatant was collected and mixed with 3 vol. ice-cold ethanol to precipitate total RNA at -20°C for 2 hours or overnight. RNA was treated with 5 units of DNase I (Fermentas) at 37°C for 45 min to remove residue DNA contamination. To isolate RNA from in vitro assays, samples were extracted by phenol:chloroform:isoamylaclcohol (P:C:I, 25:24:1, Carl-Roth, #A156.3), and RNA was precipitated using 3 vol. ice-cold ethanol at -20°C for >2h or at -80°C for 30 min.

Northern blotting

5 or 10 µg total RNA was denatured in 1× loading buffer II at 95°C for 3 min, and separated by 6% polyacrylamide (PAA) gel with 7 M urea for 2 h at 300 V. RNA was transferred to Hybond-XL membranes (GE Healthcare) by electro-blotting (1 h, 50 V, 4°C) in 1× TBE buffer. After crosslinking by 0.12 J/cm^2 UV light, membranes were hybridized with 5'-end radiolabeled DNA oligos at 42°C overnight. To radiolabel the DNA oligos, 10 pmol oligo was incubated with 25 µCi of [γ-32P]-ATP and 1 U T4 polynucleotide kinase (PNK) at 37°C for 1 h. The unincorporated ATP was removed using MicroSpin G-25 columns (GE healthcare) according to the manufacturer's instruction. The oligonucleotides sequences can be found in the Supplemental list of DNA oligonucleotides. 5S rRNA were probed using JVO-0322 as the loading control. Signal was visualized on a phosphorimager (Typhoon FLA 7000, GE Healthcare) and quantified using the AIDA image analyzer (Raytest).

In vitro RNA transcription and RNase E assays

RNA fragments were generated from DNA templates by T7 *in vitro* transcription using MEGAscript T7 kit (Life technologies) following the manufacturer's instructions. The DNA templates were prepared by PCR using gene-specific oligos containing T7 promoter sequences (see Supplemental list of DNA oligonucleotides). *In vitro*

transcription was carried out with ~200 ng DNA template at 37°C for ~6 h or at room temperature overnight. 5'P-RNAs are generated by incubating 5 µg *in vitro* transcribed RNA with 5 units tobacco alkaline phosphatase (TAP, Epicenter) at 37°C for 1 h.

RNase E assays were performed using an equimolar concentration (300 nM) of precursor RNAs, Hfq and the catalytic domain of RNase E (amino acids 1-529, NTD) in a 10 µl volume reaction, according to the established protocol (Bandyra et al., 2012; Chao and Vogel, 2016). Purified RNase E-NTD protein was provided by KJ Bandyra and BF Luisi at University of Cambridge. Prior to assays, *in vitro* transcribed RNA fragment was denatured for 1 min at 95°C, chilled on ice for 5 min. RNA was then mixed with the reaction buffer (25 mM Tris pH 7.5, 50 mM NaCl, 50 mM KCl, 10 mM $MgCl_2$, 1 mM DTT) in a 10 µl reaction volume. If necessary, the Hfq protein was added to the reaction, and incubated at 30°C for 10 min to allow Hfq-RNA complex formation. RNase E protein was then added and incubated at 30°C. Reactions were either directly loaded to denaturing PAA/7M urea gels for northern blotting analysis, or stopped by adding 100 µl of stop buffer (50 mM Tris-HCl, 0.1 % SDS and 10 mM EDTA) on ice. Following a P:C :I (25:24:1) extraction, purified RNA was used for primer extension analysis or was analyzed by northern blotting using the 5'-end-radiolabeled oligos.

Primer extension

Primer extension experiments were performed with half of the RNA extracted from *in vitro* assays. After a brief denaturation of RNA at 95°C and 5 min chilling on ice, 5 µl of the reaction mix (3 µl 5× First strand buffer, 0.5 mM each dATP, dGTP, dCTP and dTTP, and 5 mM DTT) was supplemented with 1 µl SuperScript III reverse transcriptase (Invitrogen, Thermo Fisher Scientific). Reverse transcription was carried out at 50°C for 60 min, and stopped by an incubation at 70°C for 15 min. After removal of DNA template by a digestion with 2.5 U RNase H at 37 °C for 15 min, samples were mixed with 1× loading buffer II and loaded to 6% PAA gel with a sequencing apparatus. The reference sequence ladder was prepared using the DNA fragment amplified by PCR using gene-specific oligos (Supplemental list of DNA oligonucleotides), and subjected to sequencing reaction using the DNA cycle sequencing kit (Jena Bioscience) following the manufacturer's instructions.

Electrophoretic Mobility Shift Assay (EMSA)

To prepare 5'-end-labeled RNAs for EMSA, 20 pmol in vitro synthesized RNA was first dephosphorylated at 37°C for 1 h using 10 U Calf Intestinal Alkaline Phosphates (CIP, New England Biolabs) and purified by P:C:I extraction and ethanol precipitation. The purified RNA was then 5' phosphorylated at 37°C for 1 h using 1 U T4 polynucleotide kinase (PNK, Fermentas) and 20 µCi [γ-32P]-ATP. Following a purification with MicroSpin G-25 columns to remove unincorporated ATP, labelled RNA was separated by denaturing PAGE, excised from gels and eluted by soaking gel pieces in RNA elution buffer at 4 to 8°C overnight.

EMSA assays were then performed with a 5'-end-labeled RNA and an unlabeled RNA in the presence or absence of the Hfq protein. One RNA fragment of interest was 5'-end-labeled, and denatured at 95°C for 2 min and chilled on ice for 5 min. For each 10 µl volume reaction, ~5 nM RNA was supplemented with 1× structure buffer, 1 µg yeast RNA, and various amount of unlabeled partner RNA. The reactions were incubated at 37°C for 10 min, then mixed with 3 µl native loading buffer (50 % glycerol, 0.5× TBE and 0.2 % bromphenol blue) and directly loaded to a

6% native PAGE gel running with cold 0.5× TBE buffer (4°C). Radiography signal was determined using a Typhoon FLA7000 phosphorimager and the AIDA software.

Western blotting

Bacterial samples were resuspended in 1× protein loading buffer and boilded at 95°C for 5 min. Equal amount of total protein samples (equivalent to ~0.05 OD cells) were loaded to each lane and separated by SDS-PAGE. Protein samples were transferred to a polyvinylidene fluoride (PVDF) membrane, and incubated with antibodies against GFP epitope or GroEL (Sigma) for 1 hour at room temperate or overnight at 4°C. After incubation with appropriate secondary antibodies conjugated with HRP, chemiluminescence signals were developed and analyzed with an ImageQuant LAS 4000 CCD imager (GE Healthcare) and the AIDA software (Raytest).

RNA Sequencing and data processing

For deep sequencing analysis, total RNAs were converted to cDNA libraries at Vertis Biotechnologie AG (Munich, Germany) following a standard protocol described previously (Chao et al., 2012; Westermann et al., 2016). In brief, total RNA was poly(A)-tailed using poly(A) polymerase, and the 5'PPP structure was removed using tobacco acid pyrophosphatase (TAP, epicenter). An RNA adaptor was ligated to the 5'P of the RNA. First-strand cDNA synthesis was performed using an oligo(dT)-adapter primer and the M-MLV reverse transcriptase. The resulting cDNAs were PCR-amplified to about 10-20 ng/μl using a high fidelity DNA polymerase, and purified using the Agencourt AMPure XP kit (Beckman Coulter Genomics). The cDNAs were multiplexed and sequenced using the TruSeq chemistry and Illumina HiSeq platform.

After an initial run with all samples, the samples from 44°C were re-sequenced to gain greater depth. The sequencing data from two runs were combined prior to downstream analysis. Reads with a Phred score lower than 20 were trimmed by the program fastq_quality_trimmer (FASTX toolkit), and reads shorter than 12 nt were removed using a size filter. The remaining reads were mapped to *Salmonella* Typhimurium SL1344 reference genome (NC_016810.1) and three associated plasmids (NC_017718.1, NC_017719.1, NC_017720.1) using segemehl (version 0.2.0) (Hoffmann et al., 2009) and READemption (version 0.3.5) with a mapping accuracy cutoff of 95% (Forstner et al., 2014). The 5' end of unique aligned reads was extracted from mapped BAM files using samtools (Li et al., 2009). The sequence composition of 5' nucleotide was calculated by the program fastacomposition (version 2.2.0). Pair-wise Pearson correlations were performed on 5' read coverage of all cDNA libraries. The coverage plots for all mapped 5' ends were generated by READemption with the 'first_base_only' option and stored in the wiggle format. IGB genome browser (http://bioviz.org/igb) was used to visualize the coverage plots and to generate the screenshot figures (Nicol et al., 2009). The raw reads as well as normalized coverage files have been deposited in NCBI GEO database with accession number GSE81869. Note that data from the initial run were used to analyze temperature effect in wild-type samples (Supplemental Table S7), because of the similar library sizes between 28°C and 44°C samples.

Identification of RNase E cleavage sites

The 5' end profile (number of reads per nucleotide) was compared between *rne*^{TS} and WT samples using DESeq2 with default options (v1.8.1) (Love et al., 2014); and the RNase E cleavage sites are identified by comparing the 5' end profile of WT at 44°C to that of the *rne*^{TS} samples at 44°C. The 5' ends that show >3-fold decrease (depletion) in the

rne^{TS} samples and false discovery rate (<0.05) corrected *P*-value lower than 0.05, were considered significantly different. To facilitate differential expression analyses, a pseucount of 0.0001 was added for the nucleotide with zero read count. MA plot was created to display the distribution between log fold change and mean expression, and the significant 5’ ends were shown using python. To avoid putative false-positives, candidate cleavage sites are filtered by TEX-resistant positions inferred from dRNA-Seq data (unpublished), which led to a removal of 357 positions. The final set of 22,033 RNase E cleavage sites were the 5’ ends that display >3-fold decrease (depletion) in the *rne*^{TS} samples at 44°C and statistically significant (FDR-adjusted *P* value<0.05).

Analysis of sequence features of cleavage sites

For the analyses of RNase E sites distribution, the genomic annotation of SL1344 was used and extended by custom annotations of sRNAs and UTRs. The annotations of CDS, tRNA and rRNA was collected from NCBI *Salmonella* Typhimurium SL1344 genome database (NC_016810.gff). The list of *Salmonella* essential genes is derived from a previously determined dataset (Barquist et al., 2013). The annotation of 5’UTRs was based on the primary transcription start sites (pTSS) determined previously (Kröger et al., 2013; Kröger et al., 2012). And the annotation of 3’UTRs was based on the position of downstream Rho-independent terminators, which were predicted by RNIE (Gardner et al., 2011). The cleavage sites located within these annotated features were identified using bedtools (Quinlan, 2014; Quinlan and Hall, 2010). In the absence of an upstream TSS, an arbitrary 100 nt 5'UTR was added upstream of the CDS, and similarly an arbitrary 100 nt 3'UTR was added in the absence of a terminator. The transcript length considers the distance from the pTSS to the predicated terminator. The sRNA annotations are based on previously published dataset (Chao et al., 2012; Chinni et al., 2010; Kröger et al., 2013; Kröger et al., 2012; Perkins et al., 2009) as well as unpublished annotations.

To identify the sequence and structural features of RNase E cleavage sites, sequences upstream and downstream of the identified cleavage sites were extracted. The consensus motif was identified using the MEME suite (v 4.9.1) with default parameters (Bailey et al., 2009; Bailey et al., 2015). The RNase E consensus motif logo was generated using all the sequences derived from 22,033 cleavage sites by Weblogo3 webserver (http://weblogo.threeplusone.com) (Crooks et al., 2004).

To evaluate the structural properties around RNase E cleavage sites, the secondary structure of RNA fragment in a sliding window (size: 25 nt, 5 nt/step) was predicted and the minimum free energy (MFE) was calculated using the RNAfold algorithm from the ViennaRNA Package (Lorenz et al., 2011). The MEF of the sequence in a window surrounding the cleavage sites was compared to the distribution of 1000 random shuffled sequences with the same dinucleotide composition, and a *Z*-score was calculated and used for illustration.

For meta-gene analysis, the density of cleavage sites was calculated by counting the number of sites along the specified annotation features described above, e.g. the start codon and the stop codon of respective genes. The number of cleavage sites were summarized based on the relative distance to either start or stop codon for genes, or based on the start of the established ‘Seed’ domains for 21 known Hfq-binding sRNAs. The theoretical distribution of RNase E cleavage sites was analyzed by counting the number of predicted cleavage motifs positioned in *Salmonella* genome; and the rate of occurrence at each position relative to the start or stop codon was calculated.

3'UTR-derieved sRNA candidates

To identify candidate 3'UTR-derived sRNAs, the genes that have at least one identified cleavage sites near the last nucleotide of stop codon (50 bp upstream or downstream) were extracted. The Hfq-association profile of these genes at 3'UTR was analyzed as well based on the Hfq-coIP data under the same growth condition (OD_{600} of 2.0) published previously (Chao et al, 2012). The 3'UTRs that possess a predicted Rho-independent terminator and show >3 fold enrichment in Hfq-coIP compared to WT-coIP were selected as sRNA candidates, and listed in the Supplemental Table S5.

Molecular Dynamics

Structure-based heavy atom (non-hydrogen) Gō models were designed based on the crystal structure of *E. coli* RNase E-RNA complex (PDB ID: 2C0B) (Callaghan et al., 2005) and the respective topology and parameter files were generated using the SMOG server (Noel et al., 2010). To reduce the size of the system and in turn the computational cost of the simulations we used the functional dimer of RNase E (Callaghan et al., 2005). Two separate models were constructed using this method. The first was RNase E containing a non-cognate 10-mer RNA sequence derived directly from the crystal structure (5'-ACAGUAUUUG-3'), the second RNase E model contained an RNA 10-mer with the cognate sequence of (5'-ACAGUAGUAU-3') derived from our analysis. To this end we altered the RNA sequence in the respective model using the psfgen package in the Visual Molecular Dynamics (VMD) suite (Humphrey et al., 1996). The models were simulated using native contact parameters previously described in (Whitford et al., 2009).All simulations were performed using reduced units in the GROMACS v 5.0.4 software package (Hess et al., 2008; Lindahl et al., 2001; Van Der Spoel et al., 2005) utilizing force fields available from the SMOG server (Whitford et al., 2009). Each simulation was performed using a time step of 0.0005 for a total of 20 million time-steps. Phosphate angles and bond distances were measured using the 'g_angle' and 'g_dist' packages as implemented in GROMACS v 5.0.4 (Hess et al. 2008). To assess the conformations accessible to the Lys_{112} side chain in the cognate and non-cognate complex, two additional simulations with the native contacts constrains for Lys_{112} removed were performed using the method described above.

T7-RNA sequences

>WT ArcZ:

GUGCGGCCUGAAAACAGGACUGCGCCUUUGACAUCAUCAUAAUAAGCACGGCGCAGCCACGAUUUCCCUGGUGUUGGC
GCAGUAUUCGCGCACCCCGGUCAAACCGGGGUCAUUUUU

>ArcZ G+1:

GUGCGGCCUGAAAACAGGACUGCGCCUUUGACAUCAUCAUAAUAAGCACGGCGCAGCCACGAGUUCCCUGGUGUUGGC
GCAGUAUUCGCGCACCCCGGUCAAACCGGGGUCAUUUUU

> ArcZ G+2:

GUGCGGCCUGAAAACAGGACUGCGCCUUUGACAUCAUCAUAAUAAGCACGGCGCAGCCACGAUGUCCCUGGUGUUGGC
GCAGUAUUCGCGCACCCCGGUCAAACCGGGGUCAUUUUU

>ArcZ G+2G+3:

GUGCGGCCUGAAAACAGGACUGCGCCUUUGACAUCAUCAUAAUAAGCACGGCGCAGCCACGAUGGCCCUGGUGUUGGC
GCAGUAUUCGCGCACCCCGGUCAAACCGGGGUCAUUUUU

>Mature ArcZ:

UUUCCCUGGUGUUGGCGCAGUAUUCGCGCACCCCGGUCAAACCGGGGUCAUUUUU

>WT-RprA:

GACGGUUAUAAAUCAACACAUUGAUUUAUAAGCAUGGAAAUCCCCUGAGUGAAACAACGAAUUGCUGUGUGUAGUCUU
UGCCCGUCUCCUACGAUGGGCUUUUU

>RprA G+2:

GACGGUUAUAAAUCAACACAUUGAUUUAUAAGCAUGGAAAUCCCCUGAGUGAAACAACGAAGUGCUGUGUGUAGUCUU
UGCCCGUCUCCUACGAUGGGCUUUUU

>RprA G+3:

GACGGUUAUAAAUCAACACAUUGAUUUAUAAGCAUGGAAAUCCCCUGAGUGAAACAACGAAUGGCUGUGUGUAGUCUU
UGCCCGUCUCCUACGAUGGGCUUUUU

>RprA G+2G+3:

GACGGUUAUAAAUCAACACAUUGAUUUAUAAGCAUGGAAAUCCCCUGAGUGAAACAACGAAGGGCUGUGUGUAGUCUU
UGCCCGUCUCCUACGAUGGGCUUUUU

>Mature RprA:

AUUGCUGUGUGUAGUCUUUGCCCGUCUCCUACGAUGGGCUUUUU

>*tpx* 5’ fragment

GACGUUAACUAUAAGUAAAUAGGAACAUAAUUAUGUCACAGACUGUACAUUUCCAGGGUAACCCGGUCACCGUUGCCA
ACGUUAUUCCGCAGGCUGGUAGCAAAGCACAGGCUUUUACUCUUGUCGCAAAAGAUUUGUCUGACGUUUCCCUCAGCC
AAUAUGCAGGCAAACGCAAAGUGCUGAAUAUUUUCCCAAGCAUUGAUACUGGCGUAUGCG

Supplemental list of bacterial strains used in this study.

**Trivial name**	**Strain**	**Relevant genotype/ markers**	**Reference**
Salmonella enterica serovar Typhimurium			
Wild-type	SL1344 (JVS-1574)	*Str*^{R} *hisG rpsL xyl*	(Hoiseth and Stocker, 1981), provided by D. Bumann, MPI-IB Berlin
Δ*hfq*	JVS-0584	Δ*hfq*::FRT	(Sittka et al., 2007)
WT	JVS-6999	(*rluC-rne*) IG::*cat*	(Figueroa-Bossi et al., 2009)
rne^{TS}	JVS-7000	(*rluC-rne*) IG::*cat*/ *rne*-3071 (ts)	(Figueroa-Bossi et al., 2009)
Δ*arcZ*	JVS-0082	Δ*arcZ*::Kan^{R}	(Papenfort et al., 2008)
Δ*rprA*	JVS-0088	Δ*rprA*::Kan^{R}	(Papenfort et al., 2008)
Δ*arcZ*/ WT	JVS-11411	(*rluC-rne*) IG::*cat*/ Δ*arcZ*::Kan^{R}	This study
Δ*arcZ*/ *rne*^{TS}	JVS-11412	(*rluC-rne*) IG::*cat*/ *rne*-3071 (ts)/ Δ*arcZ*::Kan^{R}	This study
Δ*rprA*/ WT	JVS-11413	(*rluC-rne*) IG::*cat*/ Δ*rprA*::Kan^{R}	This study
Δ*rprA*/ *rne*^{TS}	JVS-11414	(*rluC-rne*) IG::*cat*/ *rne*-3071 (ts)/ Δ*rprA*::Kan^{R}	This study
Escherichia coli			
	TOP10 F′	F´{l*acI*q Tn10 (Tet^{R}) *mcrA* Δ(*mrr-hsd*RMS-*mcrBC*) Φ80*lacZ*ΔM15 Δ*lacX*74 *recA*1 *araD*139 Δ(*ara-leu*)7697 *galU galK rpsL endA*1 *nupG*	Invitrogen

Supplemental list of plasmids used in this study.

Stock name	Relevant fragment	Construction and Comment	Origin /marker	Reference
pKP-8-35		pBAD control plasmid, expresses the same ~50 nt nonsense RNA.	pBR322, Amp^R	(Papenfort et al., 2006)
pKP4-13	pBAD-ArcZ	WT full-length ArcZ under the control of the inducible pBAD promoter.	pBR322, Amp^R	(Papenfort et al., 2009)
pKP15-13	pBAD-RprA	WT full-length RprA under the control of the inducible pBAD promoter.	pBR322, Amp^R	(Papenfort et al., 2015)
pKP31-5		WT mature-ArcZ under the control of the inducible pBAD promoter.	pBR322, Amp^R	(Papenfort et al., 2009)
pYC247-2		pKP4-13 derived plasmid with *arcZ* T_{+1}/G mutation. pKP4-13 was re-amplified by PCR with JVO-12432/ -12433 and self-ligated.	pBR322, Amp^R	This study
pYC248		pKP4-13 derived plasmid with *arcZ* T_{+2}/G mutation. pKP4-13 was re-amplified with JVO-12432/-12434 and self-ligated.	pBR322, Amp^R	This study
pYC249-2		pKP4-13 derived plasmid with *arcZ* $T_{+1}T_{+2}$/GG mutation. pKP4-13 was re-amplified with JVO-12432/-12435 and self-ligated.	pBR322, Amp^R	This study
pYC250-2		pKP4-13 derived plasmid with *arcZ* $T_{+2}T_{+3}$/GG mutation. pKP4-13 was re-amplified with JVO-12432/-12436 and self-ligated.	pBR322, Amp^R	This study
pYC306		pKP15-13 derived plasmid with *rprA* T_{+2}/G mutation. pKP15-13 was re-amplified by JVO-13641/-9457 and self-ligated.	pBR322, Amp^R	This study
pYC307		pKP15-13 derived plasmid with *rprA* T_{+3}/G mutation. pKP15-13 was re-amplified by JVO-13642/-9457 and self-ligated.	pBR322, Amp^R	This study
pYC308		pKP15-13 derived plasmid with *rprA* $T_{+2}T_{+3}$/GG mutation. pKP15-13 was re-amplified by JVO-13643/-9457 and self-ligated.	pBR322, Amp^R	This study
pKP-102-1	*sdaC*-10aa-*gfp*	GFP reporter plasmid carries the SdaC-10aa-gfp translational fusion.	pSC101*, Cm^R	(Papenfort et al., 2009)
pKP-125-1	*tpx*-70aa-*gfp*	GFP reporter plasmid carriers the Tpx-70aa-gfp translational fusion.	pSC101*, Cm^R	(Papenfort et al., 2009)
pYC348	*sdaC*-10aa-M'-*gfp*	pKP102-1 was re-amplified by overlapping PCR with JVO-14140/-14141.	pSC101*, Cm^R	This study
pYC349	*tpx*-70aa-M'-*gfp*	pKP125-1 was re-amplified by overlapping PCR with JVO-14243/-14244.	pSC101*, Cm^R	This study
pKF31-1	*cfa-gfp*	GFP reporter plasmid carries the *cfa*-10aa-gfp translational fusion.	pSC101*, Cm^R	(Fröhlich et al., 2013)
pYC311		pKF31-1 derivative. A T_{+2}/G mutation was introduced in pKF31-1 using overlapping PCR with JVO-13648/-13649 and Phusion polymerase.	pSC101*, Cm^R	This study
pXG-1	LacZ-GFP	Control vector containing wild type *gfp* under the control of pLtetO promoter.	pSC101*, Cm^R	(Urban and Vogel, 2007)
pXG-10		Backbone vector for constructing *gfp* translational fusions.	pSC101*, Cm^R	(Urban and Vogel, 2007)
pBAD/*Myc*-His A		pBAD backbone vector used for sRNA cloning.	pBR322, Amp^R	Invitrogen

Supplemental list of DNA oligonucleotides used in this study.

Name	Sequence	Comment
pZE-XbaI	TCGTTTTATTTGATGCCTCTAGA	Antisense oligo for *cfa* primer extension
JVO-0944	GTTTTTTTTAATACGACTCACTATAGGACGGTTAT AAATCAACACAT	Sense oligo for *rprA* T7 template, carries T7 promoter
JVO-3144	TCATGTTACCGGTAAAATACCACC	Antisense oligo to probe for STnc200
JVO-4045	TTTTTTGAATTCTAATACGACTCACTATAGGGTGC GGCCTGAAAACAG	Sense oligo for cloning of ArcZ, carries EcoRI site and T7 promoter
JVO-4049	GGGCAAAGACTACACACAGC	Antisense oligo to probe RprA
JVO-4055	GTTTTATGCATGGTTGTTTATATTACGATAATTAT TAG	Sense oligo to amplify *cfa* from +1
JVO-4208	GAATACTGCGCCAACACCAG	Antisense oligo to probe ArcZ
JVO-4652	GTTTTTTTTAATACGACTCACTATAGGGACGTTAA CTATAAGTAAATAGGAA	Sense oligo for *tpx* T7 template, carries T7 promoter
JVO-4663	TCCAGTTTCGTGATATGTTTCA	Oligo to probe STnc840
JVO-4664	GGCTAACTTGCAGAGTACAGCTT	Oligo to probe STnc850
JVO-4666	GAAGACAGGGATGGTGTCTATG	Oligo to probe STnc870/CpxQ
JVO-5902	AAAAAGCCCATCGTAGGA	Antisense oligo for RprA T7 template
JVO-5954	TACAACGGGAAAAGATTAGCG	Oligo to probe STnc890
JVO-5957	GCGCAGACTATATCACCGAAG	Oligo to probe STnc920
JVO-5961	AAAATCCAACATAGCTAAATTAAAAATAAT	Oligo to probe STnc960
JVO-5963	AGAACTTGTTGTGCCGGATG	Oligo to probe STnc980
JVO-5968	GGACATGACACACGGATTTTAC	Oligo to probe STnc2030
JVO-5969	AAAGGATAATCAAAAGTTACTTTTTATTTTTAG	Oligo to probe STnc2040
JVO-5974	GGCGATACACTCAATGTAAGGG	Oligo to probe STnc2090
JVO-5978	CTGCAAAATCAGCACTGTGG	Oligo to probe STnc2130
JVO-5980	AATACTCAGGGAATACATGCCATC	Oligo to probe STnc2150
JVO-5981	AAAGTACCACTCAACGAAGTGTTATC	Oligo to probe STnc2160
JVO-5992	TGGCCAACCATGTCGAAA	Oligo to probe RybD, partially overlap with terminator
JVO-7106	TGACCCCGGTTTGACC	Antisense oligo for ArcZ primer extension
JVO-8755	AAAAATGACCCCGGTTTG	Antisense to ArcZ terminator. Use with JVO-4045 for T7 template
JVO-8798	CGCATACGCCAGTATCAATG	Oligo to probe *tpx* mRNA
JVO-9457	5'P~TCGTTGTTTCACTCAGGG	Antisense oligo to reamplify rprA 5' part in pKP15.
JVO-9590	GAAACGTATTAGCCGTGGCG	Oligo to probe *serC* 3'UTR
JVO-9591	ACTGCCAGGACGATATCCAC	Oligo to probe *celG* 3'UTR (STnc2010)
JVO-9594	GTGGGACTAAAGCTCTTGTTCAA	Oligo to probe *argR* 3'UTR
JVO-12432	5'P~GTGGCTGCGCCGTGCTTAT	Antisense oligo to reamplify *arcZ* 5' part in pKP4.
JVO-12433	GAGTTCCCTGGTGTTGGCGCAGTA	Sense oligo to reamplify *arcZ* 3' part in pKP4, to mutate U+1.
JVO-12434	GATGTCCCTGGTGTTGGCGCAGTA	Sense oligo to reamplify *arcZ* 3' part in pKP4, to mutate U+2.
JVO-12435	GAGGTCCCTGGTGTTGGCGCAGTA	Sense oligo to reamplify *arcZ* 3' part in pKP4, to mutate U+1U+2.
JVO-12436	GATGGCCCTGGTGTTGGCGCAGTA	Sense oligo to reamplify *arcZ* 3' part in pKP4, to mutate U+2U+3.
JVO-13641	AGTGCTGTGTGTAGTCTTTGCC	Sense oligo to mutate +2U in *rprA*, use with JVO-9457
JVO-13642	ATGGCTGTGTGTAGTCTTTGCC	Sense oligo to mutate +3U in *rprA*, use with JVO-9457
JVO-13643	AGGGCTGTGTGTAGTCTTTGCC	Sense oligo to mutate U+2U+3 in *rprA*, use with JVO-9457
JVO-13648	TCGGAGAACTGGCTCACGGAATTGT	Sense oligo to mutate U+2 in *cfa*
JVO-13649	CAGTTCTCCGACGTAGAACAGAGGAA	Antisense oligo to mutate U+2 in *cfa*.

JVO-13995	ACGACACTGCTTATTGCTTTG	Antisense oligo to probe leuZ-3’ETS
JVO-14140	CAGGAGCCATAGATGGAAACCACTCAGA	Sense oligo to introduce compensatory mutations in *sdaC*
JVO-14141	ATCTATGGCTCCTGGAGGATGCTAAAA	Antisense oligo to introduce compensatory mutations in *sdaC*
JVO-14243	CCAGGGCCACCCGGTCACCGTTGCCAAC	Sense oligo to introduce compensatory mutations in *tpx*
JVO-14244	CCGGGTGGCCCTGGAAATGTACAGTCTGTG	Antisense oligo to introduce compensatory mutations in *tpx*

5’P denotes 5’-phosphorylation.

Supplemental references

Bailey, T.L., Boden, M., Buske, F.A., Frith, M., Grant, C.E., Clementi, L., Ren, J., Li, W.W., and Noble, W.S. (2009). MEME SUITE: tools for motif discovery and searching. Nucleic Acids Res *37*, W202-208.

Bailey, T.L., Johnson, J., Grant, C.E., and Noble, W.S. (2015). The MEME Suite. Nucleic Acids Res *43*, W39-49.

Bandyra, K.J., Said, N., Pfeiffer, V., Gorna, M.W., Vogel, J., and Luisi, B.F. (2012). The seed region of a small RNA drives the controlled destruction of the target mRNA by the endoribonuclease RNase E. Mol Cell *47*, 943-953.

Barquist, L., Langridge, G.C., Turner, D.J., Phan, M.D., Turner, A.K., Bateman, A., Parkhill, J., Wain, J., and Gardner, P.P. (2013). A comparison of dense transposon insertion libraries in the Salmonella serovars Typhi and Typhimurium. Nucleic Acids Res *41*, 4549-4564.

Callaghan, A.J., Marcaida, M.J., Stead, J.A., McDowall, K.J., Scott, W.G., and Luisi, B.F. (2005). Structure of Escherichia coli RNase E catalytic domain and implications for RNA turnover. Nature *437*, 1187-1191.

Chao, Y., Papenfort, K., Reinhardt, R., Sharma, C.M., and Vogel, J. (2012). An atlas of Hfq-bound transcripts reveals 3' UTRs as a genomic reservoir of regulatory small RNAs. EMBO J *31*, 4005-4019.

Chao, Y., and Vogel, J. (2016). A 3' UTR-Derived Small RNA Provides the Regulatory Noncoding Arm of the Inner Membrane Stress Response. Mol Cell *61*, 352-363.

Chinni, S.V., Raabe, C.A., Zakaria, R., Randau, G., Hoe, C.H., Zemann, A., Brosius, J., Tang, T.H., and Rozhdestvensky, T.S. (2010). Experimental identification and characterization of 97 novel npcRNA candidates in Salmonella enterica serovar Typhi. Nucleic Acids Res *38*, 5893-5908.

Crooks, G.E., Hon, G., Chandonia, J.M., and Brenner, S.E. (2004). WebLogo: a sequence logo generator. Genome Res *14*, 1188-1190.

Figueroa-Bossi, N., Valentini, M., Malleret, L., Fiorini, F., and Bossi, L. (2009). Caught at its own game: regulatory small RNA inactivated by an inducible transcript mimicking its target. Genes Dev *23*, 2004-2015.

Forstner, K.U., Vogel, J., and Sharma, C.M. (2014). READemption-a tool for the computational analysis of deep-sequencing-based transcriptome data. Bioinformatics *30*, 3421-3423.

Fröhlich, K.S., Papenfort, K., Fekete, A., and Vogel, J. (2013). A small RNA activates CFA synthase by isoform-specific mRNA stabilization. EMBO J *32*, 2963-2979.

Gardner, P.P., Barquist, L., Bateman, A., Nawrocki, E.P., and Weinberg, Z. (2011). RNIE: genome-wide prediction of bacterial intrinsic terminators. Nucleic Acids Res *39*, 5845-5852.

Hess, B., Kutzner, C., Van Der Spoel, D., and Lindahl, E. (2008). GROMACS 4: algorithms for highly efficient, load-balanced, and scalable molecular simulation. Journal of chemical theory and computation *4*, 435-447.

Hoffmann, S., Otto, C., Kurtz, S., Sharma, C.M., Khaitovich, P., Vogel, J., Stadler, P.F., and Hackermuller, J. (2009). Fast mapping of short sequences with mismatches, insertions and deletions using index structures. PLoS Comput Biol *5*, e1000502.

Hoiseth, S.K., and Stocker, B.A. (1981). Aromatic-dependent Salmonella typhimurium are non-virulent and effective as live vaccines. Nature *291*, 238-239.

Humphrey, W., Dalke, A., and Schulten, K. (1996). VMD: visual molecular dynamics. J Mol Graph *14*, 33-38, 27-38.

Kaberdin, V.R. (2003). Probing the substrate specificity of Escherichia coli RNase E using a novel oligonucleotide-based assay. Nucleic Acids Res *31*, 4710-4716.

Kröger, C., Colgan, A., Srikumar, S., Handler, K., Sivasankaran, S.K., Hammarlof, D.L., Canals, R., Grissom, J.E., Conway, T., Hokamp, K., *et al.* (2013). An infection-relevant transcriptomic compendium for Salmonella enterica Serovar Typhimurium. Cell Host Microbe *14*, 683-695.

Kröger, C., Dillon, S.C., Cameron, A.D., Papenfort, K., Sivasankaran, S.K., Hokamp, K., Chao, Y., Sittka, A., Hebrard, M., Handler, K., *et al.* (2012). The transcriptional landscape and small RNAs of Salmonella enterica serovar Typhimurium. Proc Natl Acad Sci U S A *109*, E1277-1286.

Li, H., Handsaker, B., Wysoker, A., Fennell, T., Ruan, J., Homer, N., Marth, G., Abecasis, G., Durbin, R., and Genome Project Data Processing, S. (2009). The Sequence Alignment/Map format and SAMtools. Bioinformatics *25*, 2078-2079.

Lindahl, E., Hess, B., and Van Der Spoel, D. (2001). GROMACS 3.0: a package for molecular simulation and trajectory analysis. Molecular modeling annual *7*, 306-317.

Lorenz, R., Bernhart, S.H., Honer Zu Siederdissen, C., Tafer, H., Flamm, C., Stadler, P.F., and Hofacker, I.L. (2011). ViennaRNA Package 2.0. Algorithms Mol Biol *6*, 26.

Love, M.I., Huber, W., and Anders, S. (2014). Moderated estimation of fold change and dispersion for RNA-seq data with DESeq2. Genome Biol *15*, 550.

Mackie, G.A. (2013). RNase E: at the interface of bacterial RNA processing and decay. Nat Rev Microbiol *11*, 45-57.

Martick, M., and Scott, W.G. (2006). Tertiary contacts distant from the active site prime a ribozyme for catalysis. Cell *126*, 309-320.

Misra, T.K., and Apirion, D. (1979). RNase E, an RNA processing enzyme from Escherichia coli. J Biol Chem *254*, 11154-11159.

Nicol, J.W., Helt, G.A., Blanchard, S.G., Jr., Raja, A., and Loraine, A.E. (2009). The Integrated Genome Browser: free software for distribution and exploration of genome-scale datasets. Bioinformatics *25*, 2730-2731.

Noel, J.K., Whitford, P.C., Sanbonmatsu, K.Y., and Onuchic, J.N. (2010). SMOG@ctbp: simplified deployment of structure-based models in GROMACS. Nucleic Acids Res *38*, W657-661.

Oivanen, M., Kuusela, S., and Lonnberg, H. (1998). Kinetics and Mechanisms for the Cleavage and Isomerization of the Phosphodiester Bonds of RNA by Bronsted Acids and Bases. Chem Rev *98*, 961-990.

Papenfort, K., Espinosa, E., Casadesus, J., and Vogel, J. (2015). Small RNA-based feedforward loop with AND-gate logic regulates extrachromosomal DNA transfer in Salmonella. Proc Natl Acad Sci U S A *112*, E4772-4781.

Papenfort, K., Pfeiffer, V., Lucchini, S., Sonawane, A., Hinton, J.C., and Vogel, J. (2008). Systematic deletion of Salmonella small RNA genes identifies CyaR, a conserved CRP-dependent riboregulator of OmpX synthesis. Mol Microbiol *68*, 890-906.

Papenfort, K., Pfeiffer, V., Mika, F., Lucchini, S., Hinton, J.C., and Vogel, J. (2006). SigmaE-dependent small RNAs of Salmonella respond to membrane stress by accelerating global omp mRNA decay. Mol Microbiol *62*, 1674-1688.

Papenfort, K., Said, N., Welsink, T., Lucchini, S., Hinton, J.C., and Vogel, J. (2009). Specific and pleiotropic patterns of mRNA regulation by ArcZ, a conserved, Hfq-dependent small RNA. Mol Microbiol *74*, 139-158.

Perkins, T.T., Kingsley, R.A., Fookes, M.C., Gardner, P.P., James, K.D., Yu, L., Assefa, S.A., He, M., Croucher, N.J., Pickard, D.J., *et al.* (2009). A strand-specific RNA-Seq analysis of the transcriptome of the typhoid bacillus Salmonella typhi. PLoS Genet *5*, e1000569.

Quinlan, A.R. (2014). BEDTools: The Swiss-Army Tool for Genome Feature Analysis. Curr Protoc Bioinformatics *47*, 11 12 11-11 12 34.

Quinlan, A.R., and Hall, I.M. (2010). BEDTools: a flexible suite of utilities for comparing genomic features. Bioinformatics *26*, 841-842.

Sittka, A., Pfeiffer, V., Tedin, K., and Vogel, J. (2007). The RNA chaperone Hfq is essential for the virulence of Salmonella typhimurium. Mol Microbiol *63*, 193-217.

Torres, R.A., and Bruice, T.C. (1998). Molecular dynamics study displays near in-line attack conformations in the hammerhead ribozyme self-cleavage reaction. Proc Natl Acad Sci U S A *95*, 11077-11082.

Urban, J.H., and Vogel, J. (2007). Translational control and target recognition by Escherichia coli small RNAs in vivo. Nucleic Acids Res *35*, 1018-1037.

Van Der Spoel, D., Lindahl, E., Hess, B., Groenhof, G., Mark, A.E., and Berendsen, H.J. (2005). GROMACS: fast, flexible, and free. Journal of computational chemistry *26*, 1701-1718.

Westermann, A.J., Forstner, K.U., Amman, F., Barquist, L., Chao, Y., Schulte, L.N., Muller, L., Reinhardt, R., Stadler, P.F., and Vogel, J. (2016). Dual RNA-seq unveils noncoding RNA functions in host-pathogen interactions. Nature *529*, 496-501.

Whitford, P.C., Noel, J.K., Gosavi, S., Schug, A., Sanbonmatsu, K.Y., and Onuchic, J.N. (2009). An all-atom structure-based potential for proteins: bridging minimal models with all-atom empirical forcefields. Proteins *75*, 430-441.
